# Exposome‐wide association study and predictive risk score for incident Alzheimer's disease and related dementias

**DOI:** 10.1002/alz.71842

**Published:** 2026-09-29

**Authors:** Chenshuang Li, Chenxu Zhao, Yiqun Lan, Randall J Ellis, Shakson Isaac, Sai Zhang, Gary W. Miller, Yi Sun, Shalini Jain, Hariom Yadav, Peng Gao, Chirag J Patel

**Affiliations:** ^1^ Department of Environmental Health Harvard T.H. Chan School of Public Health Boston Massachusetts USA; ^2^ Department of Biomedical Informatics Harvard Medical School Boston Massachusetts USA; ^3^ Center for Smart and Healthy Buildings Huazhong University of Science and Technology Wuhan Hubei China; ^4^ Department of Biomedical Informatics and Data Science Yale University School of Medicine New Haven Connecticut USA; ^5^ Department of Environmental Health Sciences, Mailman School of Public Health Columbia University New York New York USA; ^6^ USF Center for Microbiome Research Microbiomes Institute University of South Florida Tampa Florida USA

**Keywords:** Alzheimer's disease, dementia, exposome, ExWAS, machine learning, modifiable risk factors, risk prediction, UK Biobank

## Abstract

**INTRODUCTION:**

Environmental exposures contribute substantially to Alzheimer's disease and related dementias (ADRD), yet comprehensive exposome‐wide investigations systematically evaluating multi‐domain environmental factors remain limited and lacking utility for precision medicine.

**METHODS:**

We conducted an exposome‐wide association study (ExWAS) among 499,992 UK Biobank participants, examining 193 exposures across five domains (social, physical, chemical, lifestyle, and ecosystems). Cox proportional hazard models with Bonferroni correction identified ADRD‐associated exposures. We used the XGBoost classifier to build a predictive model and further developed an exposomic risk score (ERS) for risk stratification.

**RESULTS:**

During a 15.3‐year median follow‐up, 8881 participants (1.77%) developed ADRD, including 4000 with Alzheimer's disease. ExWAS identified 76 exposures after Bonferroni correction. The prediction model achieved area under the curve (AUC) = 0.718, outperforming demographics+apolipoprotein E (*APOE*) status alone (AUC = 0.678). The ERS stratified ADRD risk (highest vs lowest quartile hazard ratio [HR] = 2.97, 95% confidence interval [CI]: 2.59–3.41).

**DISCUSSION:**

The ERS has the potential to contribute to clinically relevant prevention strategies for ADRD.

## Highlights

- The machine learning model achieves an area under the curve (AUC) of 0.718 for Alzheimer's disease and related dementias (ADRD) prediction.
- The exposomic risk score enables precision risk stratification for ADRD prevention, but with much room for improvement.
- Key exposures of the exposome implicated in AD and ADRD include medications, lifestyle, and air pollution.
- Subgroup analyses reveal heterogeneity of predictions by age, sex, and genetics.

## 1 INTRODUCTION

Dementia represents a growing global health crisis, with prevalence projected to exceed 150 million cases worldwide by 2050, putatively influenced by the environment.^1^ Alzheimer's disease (AD), vascular dementia (VaD), Lewy body disease (LBD), and frontotemporal degeneration (FTD) are the most common forms of dementia and are collectively termed Alzheimer's disease and related dementias (ADRD).^2^, ^3^, ^4^, ^5^, ^6^ Despite advances in understanding genetic risk factors, the majority of ADRD cases cannot be attributed to genetics alone, suggesting contributions from environmental and modifiable factors.^7^, ^8^ Research suggests that nearly 10%–50% of individual differences in AD risk may be attributed to environmental influences, including urbanicity and lower education levels.^9^, ^10^

The *Lancet Commission* has identified modifiable risk factors across the age spectrum that are important for prevention.^11^ Some of these factors have been tested in important clinical trials.^12^ However, it is unclear how these modifiable factors can be operationalized to stratify the population, similar to tools in other disease domains.^13^

The exposome concept encompasses the comprehensive set of environmental exposures across the lifespan,^14^, ^15^ and provides a way to develop precision public health risk‐stratification strategies.^16^ Recent applications in neurodegenerative disease research have highlighted the potential of this framework for understanding ADRD.^17^, ^18^ The exposome‐associated contributions to ADRD include diverse domains: chemical exposures such as air pollutants; physical factors including noise; lifestyle behaviors encompassing diet and physical activity; social determinants such as education and socioeconomic status; and ecosystem factors including green space access.^19^, ^20^, ^21^, ^22^, ^23^, ^24^, ^25^, ^26^, ^27^, ^28^, ^29^, ^30^ Individual exposure studies have established associations between specific factors and dementia risk. Air pollution, particularly fine particulate matter and nitrogen dioxide, has been linked to accelerated cognitive decline.^19^, ^20^ Traffic‐related noise contributes to cognitive impairment through sleep disturbances and stress.^21^, ^22^ Greenspace could offer protective effects, with neighborhoods containing more greenspace associated with slower cognitive decline.^23^, ^24^, ^25^, ^26^ Chronic exposure to artificial light at night disrupts circadian rhythms essential for regulating amyloid beta.^29^, ^30^ Furthermore, socioeconomic factors, including neighborhood deprivation, occupational exposures, medication use, and healthcare access disparities, contribute to differential ADRD risk across populations.^31^, ^32^, ^33^

However, these exposures do not occur in isolation. The current paradigm represents only a few candidate examples from the large constellation of modifiable risk and may be biased and/or have false positives.^34^ Individuals experience complex, correlated exposure patterns.^35^ Traditional single‐exposure studies cannot capture these interdependencies. Moreover, the relative importance of different exposure domains for ADRD risk remains unclear, limiting the development of targeted prevention strategies. Demographic factors such as age, sex, and enthnicity also play critical roles in moderating environmental impacts on cognitive health.^36^, ^37^, ^38^, ^39^, ^40^, ^41^ Exposome‐wide association studies (ExWAS) offer a systematic, unbiased approach to simultaneously evaluate multiple exposures.^42^, ^43^ ExWAS can systematically screen hundreds of environmental factors while controlling for multiple comparisons, enabling unbiased discovery of novel associations that might be missed by hypothesis‐driven approaches.^44^ This is particularly valuable given the multifactorial nature of ADRD, where numerous exposures of modest individual effect may synergistically contribute to substantial risk. However, the high‐dimensional nature of exposome data necessitates advanced analytical approaches beyond traditional epidemiological methods.^45^ In addition, inspired by polygenic risk scores (PRSs), exposomic risk scores (ERSs) quantify cumulative environmental exposure effects to estimate individual‐level disease risk.^46^, ^47^, ^48^ Unlike genetic factors, environmental exposures are modifiable, enabling ERSs a broader potential for shaping public health policies and identifying high‐risk populations for targeted prevention or recruitment.^49^

Specifically, we conducted a comprehensive ExWAS examining 193 exposures across five domains (social, physical, chemical, lifestyle, and ecosystems) in 499,992 UK Biobank (UKB) participants with up to a 15‐year follow‐up (Figure 1). Our objectives were to: (1) systematically identify exposures associated with incident ADRD and AD; (2) develop and validate an exposome‐based predictive model; (3) characterize exposome variable interactions influencing ADRD risk; (4) create an ERS for population risk stratification; and (5) examine heterogeneity across demographic and genetic subgroups.

**FIGURE 1 alz71842-fig-0001:**
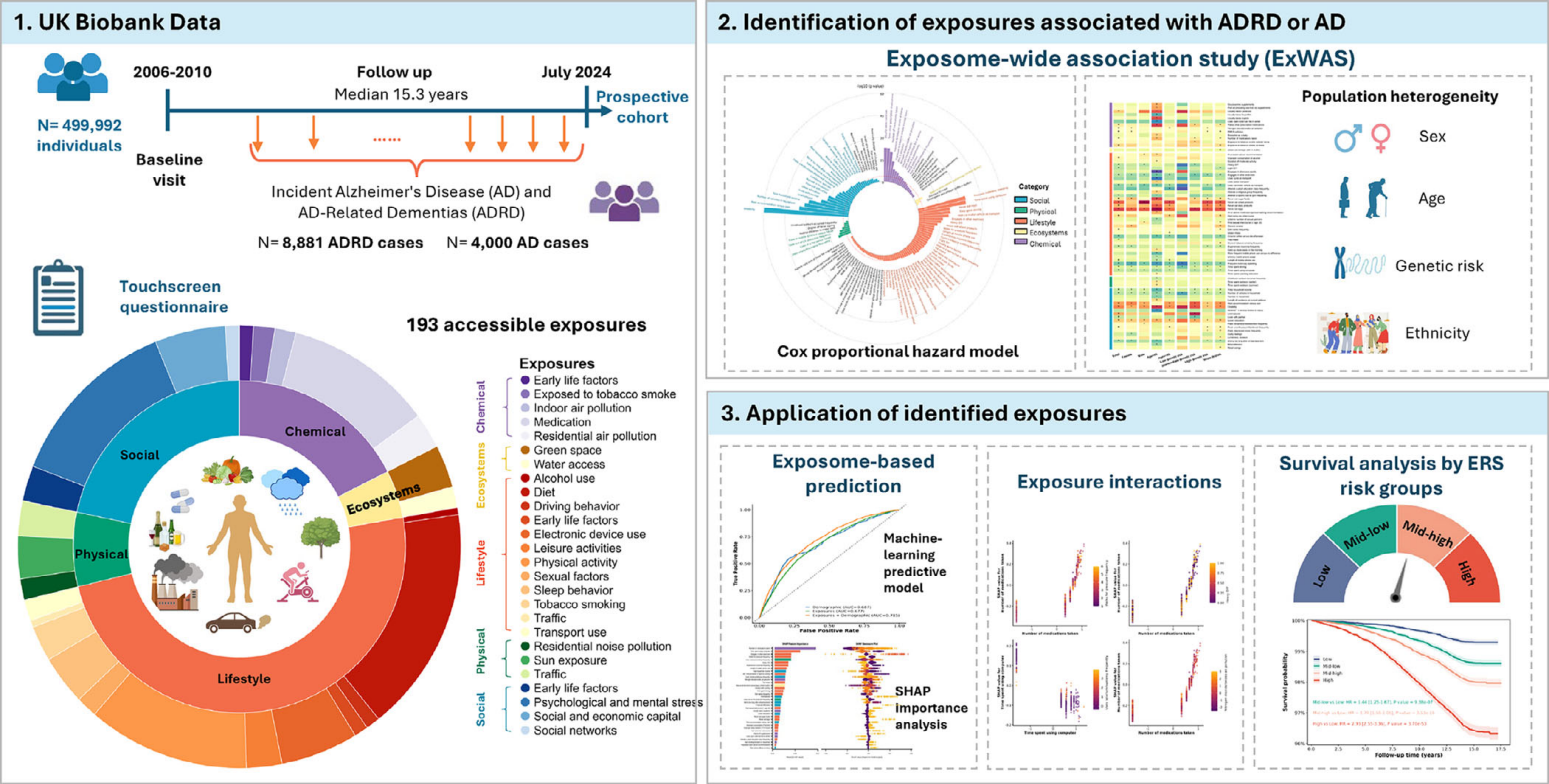
Analytic workflow of the study. In Step 1, we leveraged exposomic data (193 exposures) from 499,992 participants free of ADRD at baseline. Exposures were categorized into social, physical, lifestyle, ecosystems, and chemical domains. During a median follow‐up of 15.33 years, 8881 participants were diagnosed with ADRD, and 4000 participants were diagnosed with AD. In Step 2, unbiased exposome‐wide association analysis was conducted to identify ADRD (or AD)‐related exposures using Cox proportional hazard models. In Step 3, we explored the application of identified exposures predicting future ADRD (or AD) risk, investigating exposome variable interactions, and developing ERSs for population risk stratification. AD, Alzheimer's disease; ADRD, Alzheimer's disease and related dementias; ERS, exposomic risk score.

## 2 METHODS

### 2.1 Study population

Our study leveraged data from the UKB, a large‐scale longitudinal cohort study that recruited over 500,000 individuals 40–69 years of age across 22 assessment centers at baseline between March 1, 2006, and October 31, 2010.^50^ Participants underwent extensive baseline assessments capturing sociodemographic, lifestyle, environmental, early‐life, psychosocial, and health‐related information through comprehensive interviews and questionnaires. After excluding those with baseline ADRD or without a follow‐up record from 502,414 initial participants, our analytic cohort included 499,992 individuals with a minimum 10‐year follow‐up (median: 15.3 years) extending to July 2024 (Figure S1). The sample was randomly split into a derivation set (two‐thirds) for the main analyses and a replication set (one‐third) for internal validation. The UKB operated under ethical approval from the Northwest Multi‐centre Research Ethics Committee through the research tissue bank authorization, with written informed consent obtained from all participants.

**RESEARCH IN CONTEXT**

1. Systematic review: We reviewed the literature using PubMed examining environmental exposures and Alzheimer's disease and related dementias (ADRD) risk. Although individual studies have linked specific factors to dementia risk, comprehensive exposome‐wide investigations integrating multiple exposure domains remain scarce. Despite the emerging ADRD exposome framework, refined predictive models translating exposome‐wide associations into risk scores for evaluation of precision medicine approaches are needed.
2. Interpretation: Our study provided a comprehensive Exposome‐Wide Association Study (ExWAS) for ADRD and developed the first exposomic risk score (ERS) for precision medicine in ADRD using a large prospective cohort. We identified 76 exposures significantly associated with dementia risk across social, physical, chemical, lifestyle, and ecosystems domains. The machine learning predictive model and ERS translate these associations into easy‐to‐evaluate clinically applicable risk stratification tools.
3. Future directions: Future research should validate prospectively the ERS in diverse populations for key applications, including participant recruitment and stratification; analyze biobanked biospecimens using untargeted mass spectrometry to capture a broader range of exposures; and develop intervention trials targeting modifiable exposures.

### 2.2 Ascertainment of ADRD

We analyzed two outcomes. The first included ADRD, which comprised AD, vascular dementia, frontotemporal dementia, dementia in other diseases classified elsewhere, and unspecified dementia. The second outcome was AD alone. For AD‐specific analyses, individuals who developed non‐AD dementias were assigned as missing and excluded to ensure that the control group remained entirely free of any form of dementia. Outcomes were identified using International Classification of Diseases, Tenth Edition (ICD‐10) codes, with source‐specific code lists given in Table S1. Specifically, ADRD was defined by codes for Alzheimer's disease (F00 and G30), vascular dementia (F01), dementia in other diseases classified elsewhere (F02, which includes frontotemporal dementia coded as dementia in Pick's disease, F02.0), and unspecified dementia (F03), whereas AD was defined by F00 and G30. Dementia with Lewy bodies is not separately ascertainable in the UKB and is captured within unspecified dementia (F03). The outcome definitions were ascertained from UKB resources, including first occurrence data (Fields 131036–37, 130836–43), algorithm‐defined cases (Fields 42018–25), death certificates (Fields 40001–02), and inpatient records (Fields 41270–71, 41280–81); the first occurrence fields incorporate primary care Read terms mapped to three‐character ICD‐10. Follow‐up commenced at baseline assessment (Field 53) and terminated at outcome diagnosis, death, or last available health record date. Case identification relied on UK electronic health record linkage, with diagnoses documented predominantly by hospital specialists, primary care physicians, or vital statistics registrars.[Fig alz71842-fig-0001]

### 2.3 Exposome variables

For the exposure variables, we began with all initially available non‐genetic exposure variables from the UKB and excluded those with more than 20% missing data across the full sample. In addition, 12 key lifestyle and dietary variables with 20%–30% missingness were also retained due to their epidemiological importance. These variables were either collected at baseline or derived from baseline data (Figure S2). In total, 193 baseline‐collected or derived variables were included in the analysis (Table S2). Based on the exposome framework,^37^ these modifiable variables were categorized into five domains: (1) Ecosystems, which capture the natural and built environment, including green/blue spaces and urban land uses (e.g., natural environment percentage within a 300 m buffer, and greenspace, water, and domestic garden percentages within a 1000 m buffer); (2) Lifestyle, which encompasses behavioral factors and personal habits, such as physical activity, sleep patterns, diet, substance use, and digital media exposure (e.g., time spent using a computer, frequency of insomnia, and cheese intake); (3) Social, which reflects socioeconomic status, social networks, and psychological stress (e.g., total household income, frequency of friend/family visits, lower educational attainment, and frequent depressed mood); (4) Chemical, which includes environmental pollutants and pharmaceutical exposures (e.g., ambient particulate matter with aerodynamic diameters ≤2.5 µm (PM2.5) and ≤10 µm (PM10), as well as regular use of medications like laxatives or aspirin); and (5) Physical, which encompass environmental physical stressors such as odor and noise pollution (e.g., average daytime noise levels and traffic intensity on the nearest road).

The UKB dataset includes several types of variables: multiple‐choice categorical variables (e.g., Field 6160); single‐choice categorical variables—including binary (e.g., Field 22036), nominal multicategorical (e.g., Field 1687), and ordinal multicategorical (e.g., Field 1220); and continuous variables (e.g., Field 24500). Negative values (e.g., −1 for “Do not know,” −3 for “Prefer not to answer”) indicated non‐responses and were coded as missing across all variables. Multiple‐choice categorical variables (e.g., types of physical activities) were recoded as binary dummy variables. For single‐choice categorical variables (including binary and nominal multicategorical variables), we followed the coding schemes provided by UKB or reordered levels to reflect logical progression. Variables with only one observed level were excluded. Ordinal categorical variables were recoded to assess only linear associations; higher‐order contrasts (e.g., quadratic or cubic) were not evaluated. For continuous variables with a coding of −10 indicating values less than 1 (e.g., less than 1 h per day), responses were recoded to 0.5 to approximate a value between 0 and 1. All continuous exposure variables were standardized (mean = 0, SD = 1) after imputation, and linear associations were subsequently assessed.

For all final exposure variables included in our analysis, missing values were handled using a two‐step imputation strategy. Variables with <5% missing values were imputed using simple imputation (mode for categorical variables, mean for continuous variables). Variables with ≥5% missing values were imputed using the missRanger algorithm, a random forest–based multiple imputation method with predictive mean matching (*k* = 3 neighbors).

### 2.4 Statistical analyses

#### 2.4.1 Associations between exposures and ADRD risk

To identify ADRD‐associated exposures, we implemented an ExWAS framework. Each baseline exposure underwent Cox proportional hazards models to examine its association with incident ADRD, with significant hits defined using a conservative Bonferroni correction (*p* < 2.59 × 10^−^^4^; *α* = 0.05/193). These associations were adjusted for baseline age, sex, ethnicity, apolipoprotein E (*APOE*) status, overall health, and assessment center. An interaction with time of follow‐up was added if the hazard proportional assumption was violated (a test using Schoenfeld's residuals had a *p* < 0.0001). We report hazard ratios (HRs) as the association between exposures and outcomes.

#### 2.4.2 Exposome‐based ADRD prediction

Exposures demonstrating associations with ADRD in the ExWAS served as candidate predictive features. Spearman's rank correlation analysis was performed to mitigate multicollinearity (Figures S3 and S4). Model development and optimization utilized the derivation cohort (comprising two‐thirds of UKB participants), whereas the replication cohort (remaining one‐third) remained reserved exclusively for validation purposes. Given the imbalanced case–control ratio in the training data, we applied downsampling strategies to equalize case and control proportions while preserving age and sex distributions.

We employed the XGBoost algorithm, an optimized gradient boosting framework, for ADRD prediction.^51^ GridSearchCV facilitated hyperparameter optimization through systematic exploration of critical parameters such as the number of estimators, learning rate, and maximum tree depth. A nested cross‐validation approach guided model development, employing receiver operating characteristic—area under the curve (ROC AUC) as the optimization metric. Specifically, the framework consisted of outer fivefold cross‐validation, where each training partition underwent inner fivefold cross‐validation for parameter optimization. Feature selection was integrated into the hyperparameter tuning procedure using the least absolute shrinkage and selection operator (LASSO). Model performance was assessed using ROC AUC, with 95% confidence intervals (CIs) derived from the fivefold cross‐validation procedure. To evaluate the incremental predictive value of exposure variables beyond demographic factors (age, sex, ethnicity, overall health) and *APOE* status, we calculated the Net Reclassification Index (NRI) and Integrated Discrimination Improvement (IDI), comparing a baseline model containing only demographic factors and *APOE* status with an enhanced model incorporating both demographic factors and exposure variables.

To further elucidate the determinants of ADRD risk, we applied Shapley Additive Explanations (SHAP) analysis to interpret and visualize the contributions of predictive features in the optimal model.^52^ In addition, to identify synergistic effects among predictive exposomic features, we performed SHAP interaction analysis on the optimal model. Due to computational constraints, we sampled 500 observations for interaction computation and calculated pairwise SHAP interaction values for the top 10 features ranked by mean absolute SHAP values. The strongest feature interactions were identified and visualized using SHAP dependence plots.

#### 2.4.3 Survival analysis of exposomic risk score

To assess the combined power of the exposures, we further developed ERS from the optimized prediction model to translate complex multi‐factorial risk assessments into a clinically interpretable scoring system for population risk stratification and targeted intervention strategies. The ERS was derived through a two‐component approach encompassing feature selection and coefficient weighting. Features incorporated into the ERS comprised the important exposures retained in the final XGBoost prediction model. Each exposure was weighted by its corresponding beta coefficient obtained from Cox proportional hazards regression. The ERS was computed as a weighted linear combination of selected exposures, representing the sum of all exposure contributions. We divided the participants into quartile groups based on stratified ERS. Subsequently, we constructed Kaplan–Meier survival curves to visualize the clinical progression of ADRD events over time. Cox proportional hazard models adjusted for age, sex, *APOE* status, ethnicity, overall health, and assessment center were used to assess the association between ERS quartiles and ADRD incidence, with HRs calculated relative to the lowest risk quartile.

#### 2.4.4 Subgroup analysis

All the above analyses were further conducted within each subgroup according to age at baseline (<65 and ≥65 years), sex (female and male), genetic risks (low, intermediate, and high), and ethnicity (White British and others). Genetic risks were evaluated using the *APOE* genotype, with the presence of ε4 indicating a high genetic risk, ε3ε3 an intermediate risk, and others a low risk.

## 3 RESULTS

### 3.1 Participant characteristics

Of 502,414 participants who completed baseline assessments, 499,992 met the inclusion criteria after excluding those with prevalent ADRD at baseline. The cohort was randomly divided into a derivation set (two‐thirds, *n* = 333,328) for model development and a replication set (one‐third, *n* = 166,664) for validation. During a median follow‐up of 15.3 years (interquartile range [IQR]: 14.5–16.1 years), 8881 participants (1.77%) developed ADRD, including 4000 (0.80%) with AD specifically. Participants who developed ADRD were older at baseline and more likely to carry *APOE* ε4 alleles (Table S3). The derivation and replication sets showed comparable distributions across key demographic variables, supporting the validity of the validation approach (Tables S4 and S5). More details about the baseline characteristics of the studied population by subgroups were provided in Table S6.

### 3.2 Exposome‐wide association analysis

We conducted an ExWAS using Cox proportional hazards models, adjusting for baseline age, sex, ethnicity, *APOE* status, overall health, and assessment center, to identify exposures associated with ADRD risk (Figure 2, Tables S7, and S8). The ExWAS analysis identified 118 exposures nominal associated with ADRD risk at *p* < 0.05, of which 76 remained significant after Bonferroni correction (*p* < 2.59 × 10^−^^4^). Among them, 45 factors showed potentially detrimental effects, and 31 were potentially protective. For AD specifically, 77 exposures achieved nominal significance with 34 surviving multiple testing correction. Among them, 18 factors showed potentially detrimental effects, and 16 were potentially protective.

**FIGURE 2:**
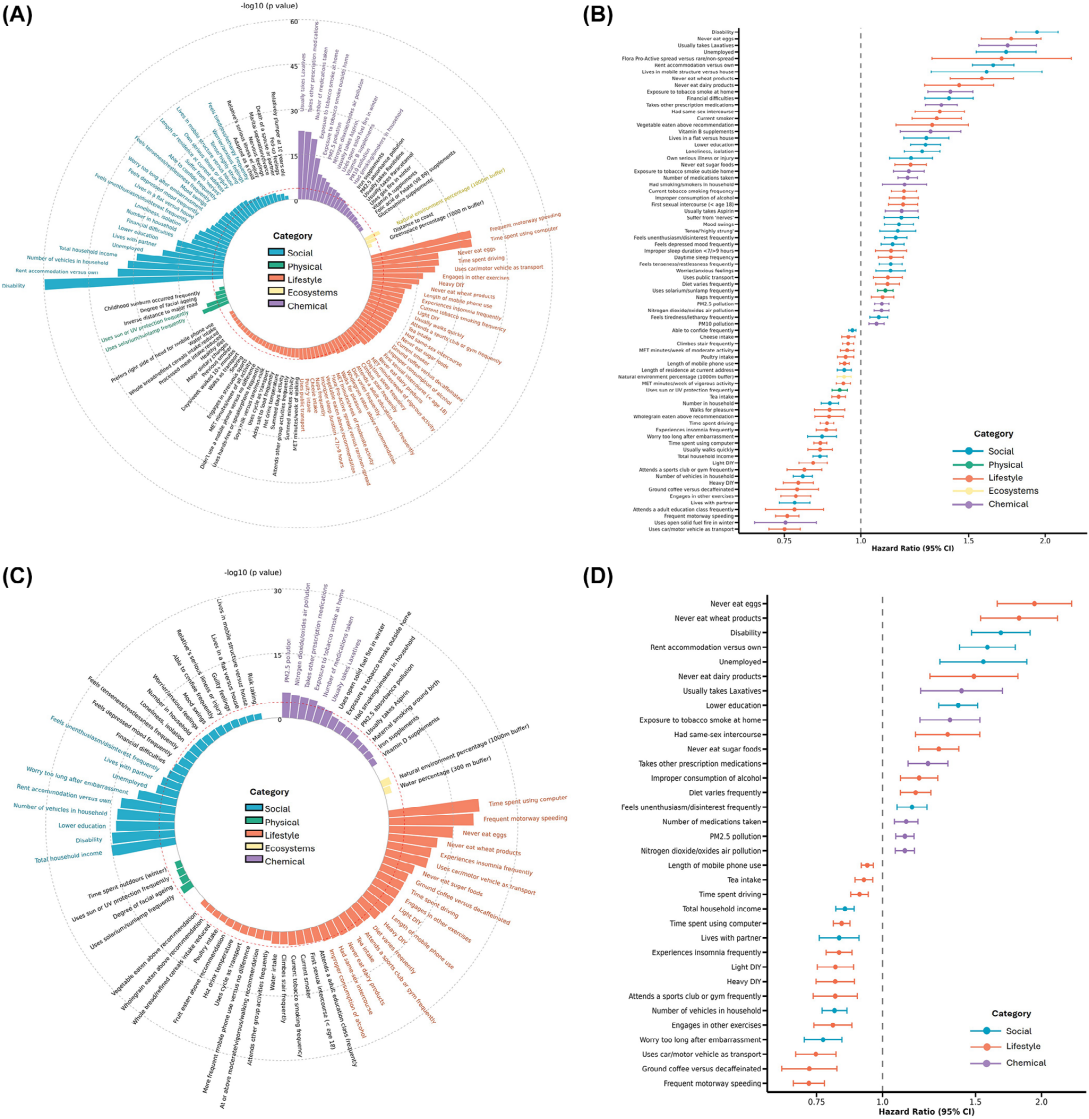
Associations between exposures and ADRD and AD risk. (A, C) Circular plots showing exposures in relation to incident ADRD (A) and AD (C). Each bar represents a significant exposure factor (*p* < 0.05) from Cox proportional hazards regression models. The radial distance indicates the strength of association as −log_10_(*p* value). The red dashed circle represents the Bonferroni‐corrected significance threshold (*p * <  2.59 × 10−4). Exposures are grouped by category and color‐coded: social, physical, lifestyle, ecosystems, and chemical. (B, D) The adjusted hazard ratios for Bonferroni‐corrected significant exposures for ADRD (B) and AD (D), in ascending order (from risk‐increasing to risk‐reducing). Dots represent hazard ratios, horizontal lines indicate corresponding 95% CIs. Hazard ratios were calculated using Cox proportional hazards regression analysis after adjustments for baseline age, sex, ethnicity, *APOE* status, overall health rating, and assessment center. AD, Alzheimer's disease; ADRD, Alzheimer's disease and related dementias; *APOE*, apolipoprotein E; CI, confidence interval.

Exposures associated with increased ADRD risk spanned all five domains (Figure 2 and Table S7). In the social domain, unemployment (vs employment: HR = 1.72, 95% CI: 1.54–1.93), financial difficulties (vs no financial difficulties: HR = 1.39, 95% CI: 1.27–1.53), and lower educational attainment (vs higher educational attainment: HR = 1.28, 95% CI: 1.21–1.35) emerged as significant risk factors. Poor housing conditions were significant risk factors for ADRD. Compared with homeownership, renting accommodation (HR = 1.64, 95% CI: 1.52–1.78), living in a mobile structure (HR = 1.60, 95% CI: 1.30–1.98), and living in a flat (HR = 1.17, 95% CI: 1.02–1.33) were associated with higher risk of ADRD, potentially reflecting financial difficulties, increased chronic stress, and less access to health‐promoting resources. Lifestyle factors encompassed never eating eggs (vs ever eating eggs: HR = 1.76, 95% CI: 1.57–1.97), current tobacco smoking (per one‑level increase from lower to higher frequency: HR = 1.18, 95% CI: 1.12–1.23), and improper consumption of alcohol (vs proper consumption: HR = 1.18, 95% CI: 1.11–1.24). Sleep‐related exposures, including frequent daytime dozing (per one‑level increase from lower to higher frequency: HR = 1.12, 95% CI: 1.07–1.17) and sleep duration deviations from optimal ranges (vs proper sleep duration: HR = 1.12, 95% CI: 1.06–1.19), demonstrated significant associations with ADRD, consistent with emerging evidence linking sleep disturbances to amyloid accumulation. Chemical exposures, particularly nitrogen dioxide air pollution (HR = 1.08 per standard deviation [SD] increase [9.70 µg/m^3^], 95% CI: 1.05–1.11), were associatied with higher ADRD risk. Multiple medication use showed particularly strong associations with incident ADRD (HR = 1.19 per SD increase [2.67 medications], 95% CI: 1.15–1.24), likely reflecting underlying health complexity rather than direct pharmacological effects.

Potential protective factors included engagement in physical activities such as walking for pleasure (per one‐level increase from lower to higher duration: HR = 0.89, 95% CI: 0.83–0.94), participation in other exercises (vs no exercises: HR = 0.78, 95% CI: 0.74–0.83), and engagement in heavy do‐it‐yourself (DIY) activities (vs no exercises: HR = 0.79, 95% CI: 0.75–0.84). Dietary factors, including cheese intake (per one‑level increase from lower to higher frequency: HR = 0.95, 95% CI: 0.93–0.98) and poultry intake (per one‑level increase from lower to higher frequency: HR = 0.94, 95% CI: 0.92–0.97), were associated with reduced risk. Factors related to electronic device use, including computer (HR = 0.86 per SD increase [1.37 hours/day], 95% CI: 0.84–0.88) and mobile phone use (per one‑level increase from lower to higher frequency: HR = 0.94, 95% CI: 0.92–0.96), were significantly associated with reduced risk of ADRD. Social connection, including living with a partner (vs living with others: HR = 0.78, 95% CI: 0.74–0.83), demonstrated a potential protective effect as well. Higher household income (per one‐level increase from lower to higher income: HR = 0.86, 95% CI: 0.84–0.88) and household vehicle ownership (from lower to higher ownership: HR = 0.80, 95% CI: 0.78–0.83) were significantly associated with lower ADRD risk.

The proportional hazards assumption was violated by seven exposures in the ADRD models, but none in the AD models (*P* < 0.0001). Time‐interaction models confirmed that these factors exhibited time‐dependent associations with ADRD: the risk effects of psychological and sleep‐related factors were highest at early follow‐up and attenuated over time, whereas the early protective benefits of physical activities also weakened throughout the follow‐up period (Table S9).

### 3.3 Subgroup heterogeneity in ExWAS

Stratified analyses revealed notable heterogeneity in exposure—ADRD associations across demographic and genetic subgroups (Figure 3, Tables S10 and S11). Several factors demonstrated consistent associations with ADRD risk across all examined subgroups. The strongest risk factors were living in a mobile structure, never eating eggs, exposure to tobacco smoke at home, mental health problems (depression, anxiety, loneliness), and air pollution (PM_2.5_, nitrogen dioxide), consistently increasing ADRD risk across most subgroups. Strong potential protective factors included physical activities (heavy DIY, engaging in other exercises), and socioeconomic advantages such as living with a partner, higher household income, and a greater number of household vehicles, with these associations persisting across sex, age, genetic risk, and ethnicity.

**FIGURE 3:**
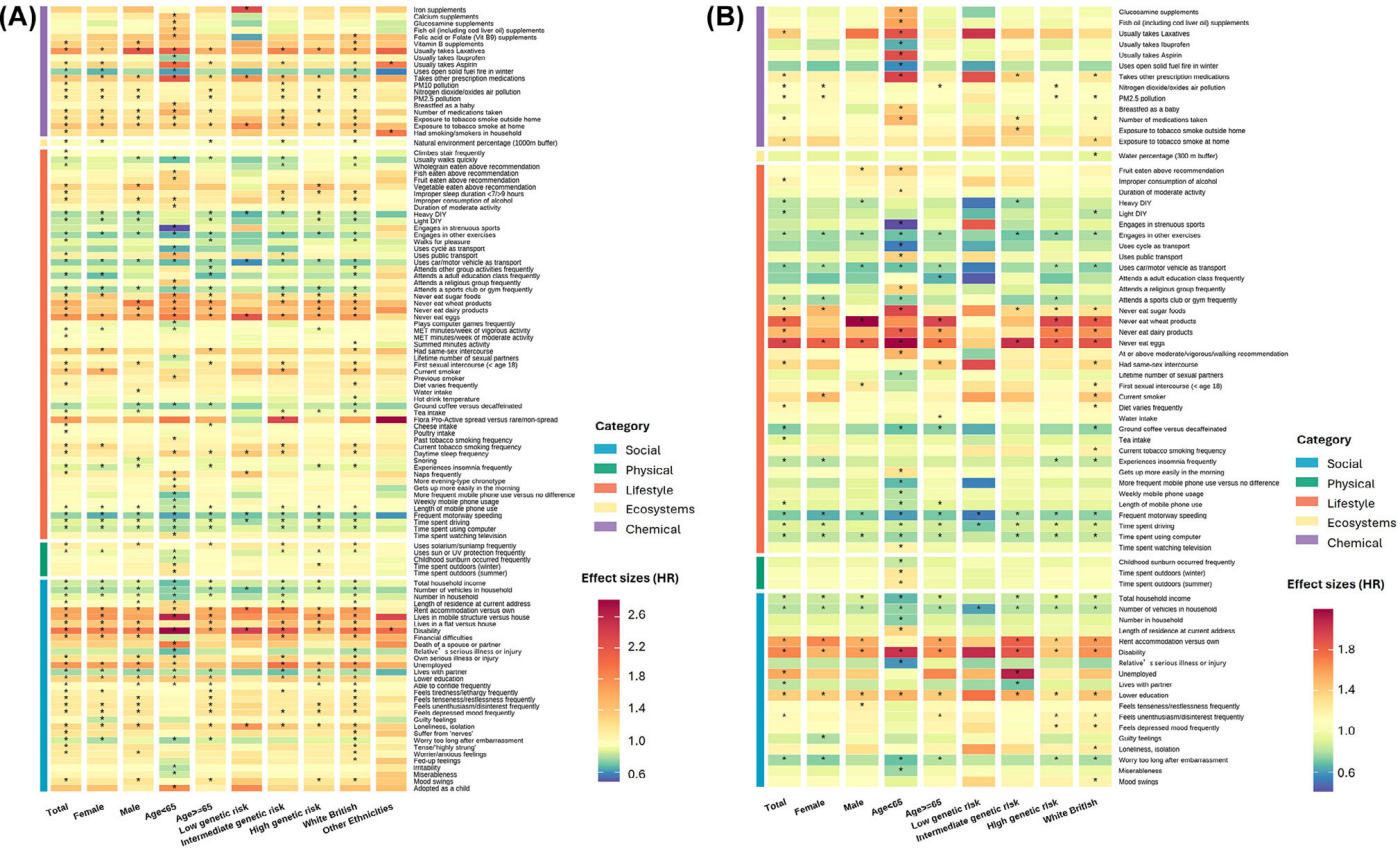
Summary heat map for significant factors in ExWAS analysis across the full sample and subgroups. (A) ADRD. (B) AD. The color of cells indicates the effect sizes (HR). HRs were derived from Cox proportional hazards regression models adjusted for baseline age, sex, ethnicity, *APOE* status, overall health rating, and assessment center. Asterisks represent significant associations after Bonferroni correction (*P*  <  2.59 × 10−4). For AD in other ethnicities, no exposures remained significant after Bonferroni correction and were therefore not displayed. AD, Alzheimer's disease; ADRD, Alzheimer's disease and related dementias; *APOE*, apolipoprotein E; ExWAS, exposome‐wide association study; HR, hazard ratio.

Regarding sex differences, males showed stronger risk associations with alcohol consumption and certain dietary patterns (never eating wheat products), whereas females demonstrated greater potential protective benefits from frequent participation in adult education classes and exposure to natural environments. Age‐related patterns revealed that younger individuals (<65 years) showed stronger risk associations with lifestyle factors including alcohol consumption, smoking (past tobacco smoking frequency, previous smoker status), unemployment, and serious illness or injury, while demonstrating greater potential protective benefits from vigorous physical activities such as strenuous sports, cycling for transport, and higher weekly metabolic equivalent minutes of vigorous activity. In contrast, older individuals (≥65 years) displayed more pronounced risk associations with chemical exposures (nitrogen dioxide/oxides and PM_2.5_) and mental health factors (frequent tiredness, tension, lack of enthusiasm, depressed mood, and guilty feelings), while showing stronger potential protective effects from moderate physical activities (heavy and light DIY activities, walking for pleasure) and social engagement (attending other group activities and adult education classes frequently). Genetic risk stratification by *APOE* status revealed that certain environmental exposures exhibited stronger associations among those with high genetic risk (ε4 carriers). Among individuals with high genetic risk, living in a mobile structure (vs a house), mood swings, and MET minutes per week of vigorous physical activity were particularly significant factors. In contrast, fewer significant exposures were identified among those with low genetic risk, with exposure to tobacco smoke at home, heavy DIY activities, never eating eggs, frequent napping, and loneliness/isolation showing stronger associations. Ethnicity comparisons showed that although White British participants displayed more statistically significant associations, this may be due to the larger sample size.

### 3.4 Exposome‐based predictive model

We applied the XGBoost algorithm for exposome‐based prediction. The XGBoost prediction model incorporating ExWAS‐identified exposures achieved an AUC of 0.718 (95% CI: 0.709–0.727) for ADRD prediction in the replication set, outperforming the demographics and *APOE* status only model (AUC = 0.678, 95% CI: 0.668–0.687) (Figure 4A). For AD specifically, the exposome enhanced model achieved an AUC of 0.724 (95% CI: 0.710–0.738), compared with 0.680 (95% CI 0.666–0.693) for the model including demographics and APOE status alone (Figure 4D). Training set performance evaluated by fivefold cross‐validation (Table S12) was comparable to test set results, indicating minimal overfitting. Beyond AUC, the exposome‐enhanced model showed well‐balanced performance metrics: for ADRD, sensitivity was 62.7% and specificity 70.1%, vs 53.8% and 78.9% for the model including demographics and *APOE* status alone. Similar improvements were observed for AD prediction, with the combined model achieving 60.7% sensitivity and 74.3% specificity (Table S13). The incremental value of exposome data was quantified using reclassification metrics. For ADRD, the continuous NRI demonstrated 28.4% improvement for events and 10.4% for non‐events, with an IDI of 3.7% for the full sample (Figure 4A). Similar improvements were observed for AD prediction (continuous NRI 35.6% for events, 30.2% for non‐events; IDI 8.7%) (Figure 4D).

**FIGURE 4:**
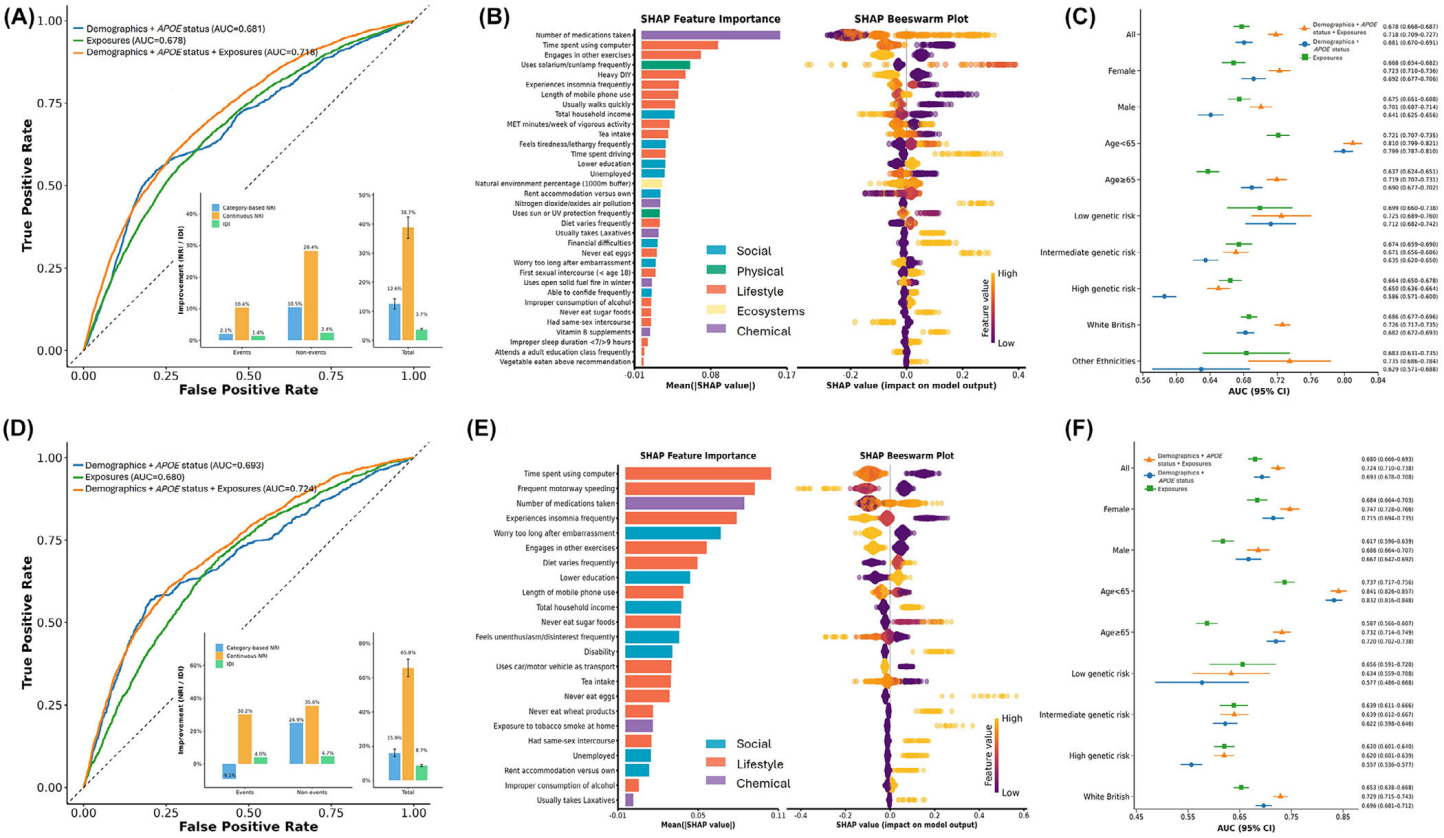
Exposome‐based predictive models for ADRD and AD risk. (A, D) ROC curves for ADRD and AD prediction models. NRI and IDI comparing models without and with exposures. (B, E) ADRD and AD prediction model interpretability through SHAP analysis. The left panel shows mean absolute SHAP values for each feature. The right panel (beeswarm plot) displays SHAP values for individual predictions. (C, F) Performance of exposome‐based predictive models in subgroups. AD, Alzheimer's disease; ADRD, Alzheimer's disease and related dementias; IDI, Integrated Discrimination Improvement; NRI, Net Reclassification Index; ROC, Receiver Operating Characteristic; SHAP, SHapley Additive exPlanations.

Subgroup analyses confirmed consistent predictive performance across age, sex, genetic risk, and ethnicity strata (Figure 4C, 4F). The combined model (Demographics + *APOE status* + Exposures) consistently outperformed the individual models across most strata, although predictive performance varied significantly by age and genetic risk. Regarding age groups, the combined model achieved its highest AUC of 0.810 (95% CI: 0.799–0.821) in the age <65 group, whereas its performance dropped to 0.719 (95% CI: 0.707–0.731) in those 65 years of age and over. The models performed best in the low genetic risk subgroup (combined model AUC = 0.725, 95% CI: 0.689–0.760) and showed the weakest performance in the high genetic risk subgroup (combined model AUC = 0.586, 95% CI: 0.571–0.600). Although the point estimate for the combined model was slightly higher in females (AUC = 0.723; 95% CI: 0.710–0.736) than in males (AUC = 0.701; 95% CI: 0.687–0.714), their 95% CIs exhibited overlap. Across ethnic groups, the combined model showed comparable point estimates between White British (AUC = 0.726, 95% CI: 0.717–0.735) and Other Ethnicities (AUC = 0.735, 95% CI: 0.686–0.784), although the latter exhibited wider CIs, likely reflecting a smaller sample size.

### 3.5 Feature importance and SHAP analysis

SHAP analysis identified the most influential predictors in the optimized model (Figure 4B). For ADRD prediction, the number of medications taken emerged as the strongest predictor (risk‐enhancing), followed by time spent using a computer (protective), engaging in other exercises such as swimming, cycling, keep fit, and bowling (protective), using solarium/sunlamp frequently (risk‐enhancing), and heavy DIY activities such as weeding, lawn mowing, and carpentry (protective). The SHAP beeswarm plots revealed the directional effects and magnitude of each predictor's contribution to individual risk predictions (Figure 4B). A higher number of medications and elevated nitrogen dioxide air pollution were positively associated with increased ADRD risk, whereas higher household income, a faster walking pace, and frequent vigorous activity served as significant potential protective factors. Social determinants, including unemployment and lower education, also consistently contributed to higher risk predictions. Overall, these results indicate that the exposomics model's predictive power is primarily driven by a multi‐dimensional interplay of polypharmacy, lifestyle habits, and socioeconomic status. For AD specifically, time spent using a computer, frequent motorway speeding, total household income, and other exercise engagement demonstrated high feature importance alongside medication use and insomnia frequency (Figure 4E).

### 3.6 Exposure interactions

SHAP interaction analysis identified significant non‐additive effects between exposure pairs (Figure 5). For ADRD, the most prominent interaction was observed between time spent using a computer and frequent use of solarium/sunlamps, followed by the interaction between engaging in other exercises (e.g., swimming, cycling, keep fit, bowling) and heavy DIY activities (e.g., weeding, lawn mowing, carpentry). Notably, the number of medications taken showed significant interactions with multiple lifestyle factors (computer use, driving time, tea intake, etc.), suggesting that multimorbidity status may affect the impact of lifestyle factors on ADRD risk.

**FIGURE 5:**
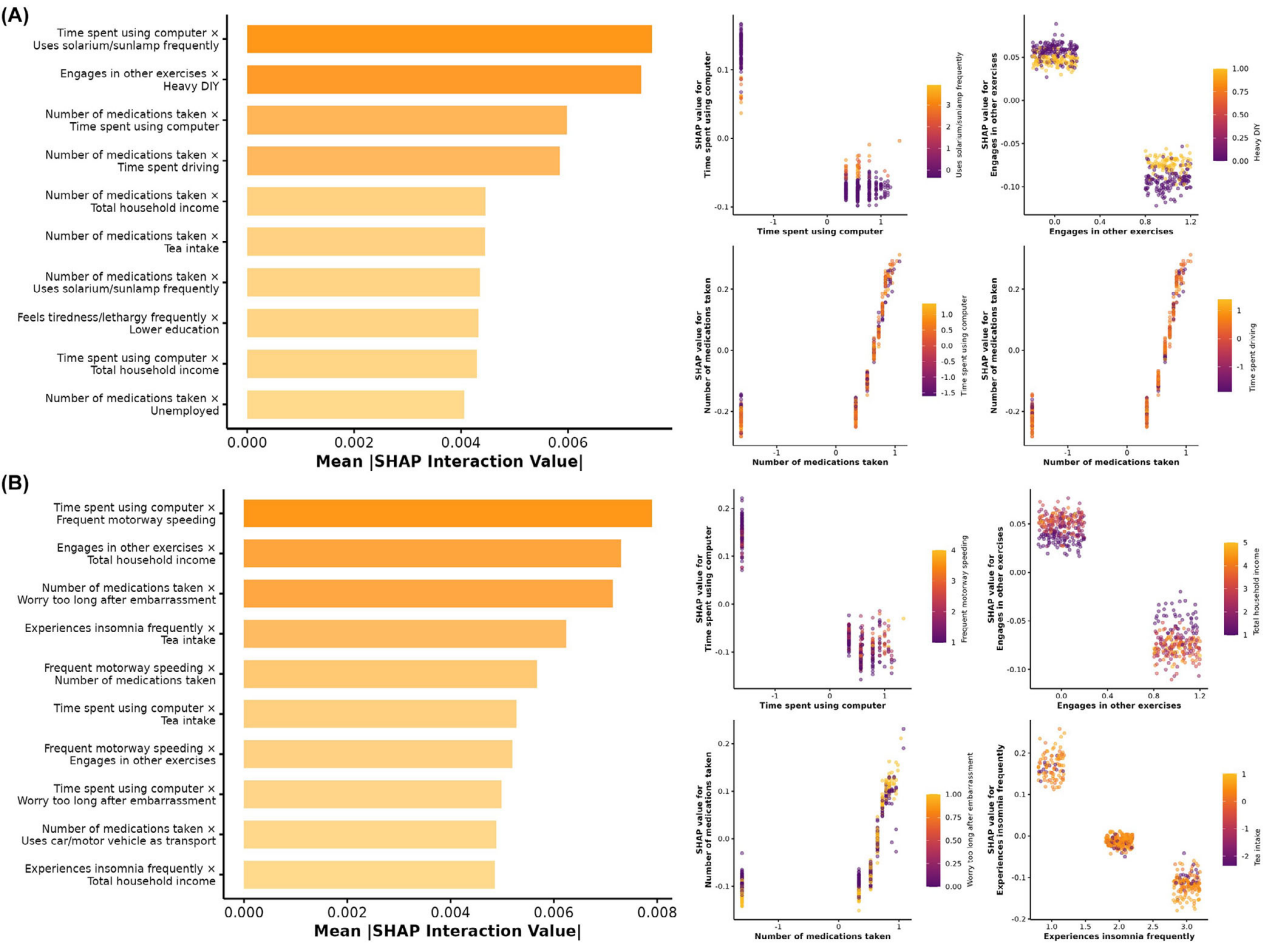
SHAP interaction effects for exposome‐based predictive models. (A) ADRD. (B) AD. Left panels show the top 10 exposome variable interaction pairs ranked by mean absolute SHAP interaction values. The right panels show SHAP dependence plots for top interaction pairs, revealing how feature combinations jointly influence risk predictions. AD, Alzheimer's disease; ADRD, Alzheimer's disease and related dementias; SHAP, SHapley Additive exPlanations.

### 3.7 Exposomic risk score and survival analysis

The ERS derived from the XGBoost model effectively stratified the population into distinct risk groups. ADRD and AD ERS were differently distributed between cases and control groups (Figure S5). Kaplan–Meier survival analysis demonstrated clear separation between ERS quartiles, with the highest risk quartile (Q4) showing substantially accelerated ADRD onset compared to the lowest risk quartile (Q1) (Figure 6A). Cox regression confirmed graded associations: compared to Q1, HRs were 1.43 (95% CI: 1.24–1.66) for Q2, 1.83 (95% CI: 1.59–2.10) for Q3, and 2.97 (95% CI: 2.59–3.41) for Q4 (*p* for trend < 0.001). For AD specifically, similar risk stratification was observed (Figure 6C), with the highest to lowest quartile HR of 2.80 (95% CI: 2.27–3.44).

**FIGURE 6:**
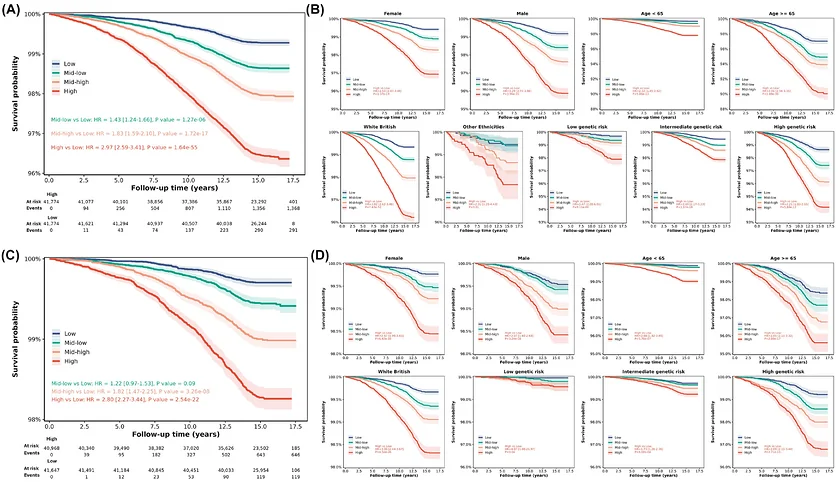
Kaplan–Meier survival curves stratified by ERS quartiles. (A, C) Kaplan–Meier curves for ADRD and AD in the total population. (B, D) Subgroup‐specific survival curves stratified by sex, age, *APOE* status, and ethnicity. Participants were divided into ERS quartiles: Q1 (low risk), Q2 (mid‐low), Q3 (mid‐high), and Q4 (high risk). Shaded areas represent 95% CIs. AD, Alzheimer's disease, ADRD, Alzheimer's disease and related dementias; *APOE*, apolipoprotein E; CI, confidence interval; ERS, exposomic risk score.

Subgroup stratified survival analyses demonstrated consistent ERS performance across sex, age groups, *APOE* status, and ethnicity (Figure 6B, 6D), although effect magnitudes varied. For ADRD (Figure 6B), the ERS demonstrated a consistent and significant ability to stratify risk across all subgroups. Notably, the discriminative power of the ERS was most pronounced in the Age ≥65 group, where the high‐ERS group showed a steeper decline in survival probability compared to the age <65 group. The score remained a potent predictor regardless of genetic predisposition, showing clear survival separation even within the high genetic risk subgroup. Similarly, for AD (Figure 6D), the ERS maintained strong predictive value, particularly in older participants and those with high genetic susceptibility. While the overall event rates were lower for AD compared to ADRD, the Age ≥65 subgroup showed a marked divergence in survival curves starting around 10 years of follow‐up for those in the high‐ERS quartile. Across both outcomes, the consistency of the HRs and the clear separation of Kaplan–Meier curves across diverse demographic and genetic backgrounds highlight the broad applicability of the ERS in identifying individuals at high risk for ADRD.

Although *APOE* high‐risk status conferred the strongest individual effect (for ADRD high vs low HR = 3.47, 95% CI: 3.00–4.01; for AD high vs low HR = 5.21, 95% CI: 4.12–6.59), the *APOE* variable primarily distinguished ε4 carriers (high‐risk) from non‐carriers (low‐ and intermediate‐risk) (Figure S6). Notably, the ERS demonstrated more granular risk stratification with four distinct categories showing dose–response relationships, providing more actionable stratification with graded hazard ratios across four categories (Figure 6). Of note, the survival curves for ERS diverged as early as 2.5 years into follow‐up–‐significantly earlier than the divergence observed for *APOE* status. This temporal lead time suggests that exposomic interventions may offer a critical window for modifying risk trajectories well before the deterministic effects of genetic susceptibility fully manifest, underscoring the vital role of modifiable factors in early ADRD prevention.

## 4 DISCUSSION

This comprehensive ExWAS of 499,992 UKB participants with over 15 years of follow‐up provides the most extensive systematic evaluation of multi‐domain environmental exposures and ADRD risk to date. Our analyses identified 76 exposures significantly associated with ADRD and 34 with AD after stringent multiple testing correction, spanning social, lifestyle, ecosystem, physical, and chemical domains. Of note, the machine learning predictive model incorporating these exposures significantly improved risk prediction beyond demographics and *APOE* status alone, and the derived Exposomic Risk Score demonstrated robust population risk stratification with a nearly threefold increased hazard for the highest versus lowest risk quartiles.

Our study provided comprehensive ExWAS for ADRD in a large prospective cohort, revealing multi‐domain exposures associated with ADRD. Medication count emerged as the strongest predictor, which likely reflects underlying comorbidity burden and polypharmacy effects rather than a direct causal relationship, highlighting the importance of comprehensive health status in dementia risk assessment.^53^, ^54^ The observed associations with sleep‐related exposures merit particular attention. Poor sleep quality and disrupted circadian rhythms impair glymphatic clearance of amyloid beta and tau proteins, potentially accelerating neurodegenerative processes.^55^, ^56^ Our findings that daytime sleep frequency and non‐optimal sleep duration predict ADRD risk were consistent with emerging evidence linking sleep disturbances to amyloid accumulation.^56^, ^57^ Similarly, the strong potential protective effects of physical activity align with mechanistic studies.^58^, ^59^ These biological pathways provide plausible mechanisms through which the exposures identified in our ExWAS may influence ADRD risk. Social determinants, including educational attainment and home ownership, showed potential protective associations with ADRD risk, consistent with previous evidence.^60^, ^61^ Air pollution exposures, particularly nitrogen dioxide, emerged as significant risk factors consistent with the growing literature on environmental neurotoxicants and neurodegeneration.^19^, ^20^ In addition, consistent with previous studies showing that digital technologies are associated with a reduced risk of cognitive impairment,^62^, ^63^ our findings further reveal that the use of electronic devices is linked to lower ADRD risk, highlighting new opportunities to leverage digital inclusion and services in public health strategies. Previous studies indicate that genetic susceptibility, such as AD polygenic risk scores (or PRSs), plays a determinant role in AD risk.^64^ Our study suggests that modifiable exposomic factors may also contribute meaningfully, but need to be evaluated to rule out reverse causation and confounding and to gauge clinical impact.

Our findings substantially extend prior work on associations between individual exposure and dementia. The *Lancet Commission* identified 14 modifiable risk factors accounting for ≈40% of dementia cases^11^; our ExWAS identified many of these factors while revealing additional associations across broader exposure domains. The multi‐domain approach enabled direct comparison of effect magnitudes across exposure types. In addition, the observed lifestyle–social interactions indicate that lifestyle‐related potential protective effects may differ across individuals with varying backgrounds. Recent environmental exposome studies in AD have begun adopting similar systematic approaches. Li et al. examined associations between neighborhood environmental factors and cognitive function among older adults with and without preclinical AD, finding significant effects of walkability, green space, and light pollution.^65^ Our population‐level findings complement this work by demonstrating that these exposures also predict ADRD over extended follow‐up, and by contextualizing environmental factors within the broader exposome framework.

The interactions between medication count and multiple lifestyle factors (computer use, tea intake, driving time, etc.) suggest that multimorbidity status may modify the effects of lifestyle factors on ADRD risk. The number of medications taken, as a proxy for health complexity,^66^ interacting with multiple lifestyles, indicates that individuals with greater health complexity may experience differential benefits from lifestyle modifications.

Notably, this study first developed predictive models translating exposome‐wide associations into actionable risk scores. Using a large longitudinal population cohort, the UKB, we developed a novel exposome‐based ADRD risk prediction model across multiple domains of the exposome. Our findings demonstrate that the ERS provides risk stratification through four graded categories with dose–response relationships, compared to the binary carrier/non‐carrier classification of *APOE*. However, the earlier divergence of ERS survival curves within the first 2.5 years may be partly attributable to confounding, while also potentially reflecting an early influence of environmental factors on ADRD risk trajectories. It also appears that the ERS captures individual‐level modifiable risk factors independent of genetic predisposition.^49^

The subgroup heterogeneity observed in our analyses aligns with evidence for differential vulnerability to environmental exposures across demographic groups, but needs to be further evaluated.^36^, ^37^, ^38^, ^39^, ^40^, ^41^ Sex‐specific patterns in exposure effects may reflect differences in exposure patterns, biological susceptibility, or health behaviors.^67^ ExWAS analyses identified distinct HR patterns across age groups for individual exposures, exposome‐based prediction models demonstrated higher discriminative performance in individuals <65 years, and survival analyses showed that high‐risk individuals aged ≥65 years experienced a steeper risk trajectory. Older adults may experience amplified risk consequences from decades of cumulative exposures, despite greater heterogeneity in aging processes, reducing discriminative ability.

These findings have several implications for ADRD prevention and research. First, the identification of multiple modifiable exposures across diverse domains supports the feasibility of multi‐component intervention strategies.^68^ Second, the ERS demonstrates potential for population risk stratification that could inform targeted prevention efforts.^13^ Finally, the predictive performance achieved using exposome data supports continued investment in comprehensive exposure assessment in longitudinal cohorts and clinical settings, particularly as prognostics for AD emerge.^69^

Several limitations warrant consideration. First, UKB participants are healthier and more socioeconomically advantaged than the general UK population, potentially limiting generalizability.^70^ Second, exposures were assessed at a single baseline time point; changes in exposure status during follow‐up could not be captured. Third, ADRD ascertainment relied on linked health records, which may underdiagnose cases, and the lack of biomarker measurements in the UKB may have led to misclassification of AD, given its importance in distinguishing AD from other forms of dementia at onset.^71^ Fourth, *APOE* ε4 is associated with dementia subtypes to differing degrees (strongest for AD, modest for vascular dementia, and not consistently established for frontotemporal degeneration), so adjustment for *APOE* status carries unequal information across the components of our composite ADRD outcome; because genotype precedes and is independent of adult exposures, however, this is unlikely to bias the observed exposure–outcome associations. Fifth, some exposure measurements were self‐reported, introducing potential recall bias. Sixth, despite adjustment for confounders, residual confounding cannot be excluded in this observational study. Seventh, the predominantly White British composition limits the assessment of racial and ethnic disparities.^37^, ^38^ Finally, although machine learning methods captured complex exposure patterns, external validation in independent cohorts is needed.

In conclusion, this comprehensive ExWAS identifies numerous modifiable exposures associated with ADRD risk across social, physical, lifestyle, ecosystem, and chemical domains. The multi‐domain predictive model and ERS demonstrate the clinical utility of comprehensive exposure assessment for ADRD risk stratification. These findings support the development of multi‐component intervention strategies targeting modifiable exposures for ADRD prevention, which may prove essential for reducing the growing burden of dementia.

## CONFLICT OF INTEREST STATEMENT

The authors declare no conflicts of interest related to this work. Author disclosures are available in the Supporting Information.

## Supporting information




**Supporting Information**: alz71842‐sup‐0001‐ICMJE

**Supporting Information**: alz71842‐sup‐0002‐SuppMat

## Data Availability

The data used in the present study are available from UK Biobank (UKB) with restrictions applied. Data were used under license and are thus not publicly available. Access to the UKB data can be requested through a standard protocol (https://www.ukbiobank.ac.uk/register‐apply/). No new software, package, or algorithm was developed. The code used in this study can be accessed at https://github.com/Chenshuang‐Li/ExWAS_ADRD.

## References

[1] Nichols E , Steinmetz JD , Vollset SE , et al. Estimation of the global prevalence of dementia in 2019 and forecasted prevalence in 2050: an analysis for the global burden of disease study 2019. Lancet Public Health. 2022;7:e105‐e125. doi:10.1016/S2468-2667(21)00249-8 PMC8810394 34998485

[2] U.S. Department of Health and Human Services . National plan to address Alzheimer's disease: 2024 update. U.S. Department of Health and Human Services; 2024. Accessed August 14, 2026. https://aspe.hhs.gov/sites/default/files/documents/dc2ff0be0e08df15971fce57cb8e5c7a/napa-national-plan-2024-update.pdf

[3] National Institute of Neurological Disorders and Stroke . Focus on Alzheimer's disease and related dementias. National Institutes of Health. Accessed August 14, 2026. https://www.ninds.nih.gov/current‐research/focus‐disorders/focus‐alzheimers‐disease‐and‐related‐dementias

[4] Pirraglia E , Osorio RS , Glodzik L , Ashebir Y , Shao Y . Subtypes of multiple‐etiology dementias and the heterogeneous impact of APOE variants. Alzheimers Dement. 2025;21(11):e70872. doi:10.1002/alz.70872 PMC12591992 41198610

[5] Albrecht JS , Hanna M , Randall RL , Kim D , Perfetto EM . An algorithm to characterize a dementia population by disease subtype. Alzheimer Dis Assoc Disord. 2019;33(2):118‐123. doi:10.1097/WAD.0000000000000295 PMC6941141 30681435

[6] Montine TJ , Koroshetz WJ , Babcock D , et al. Recommendations of the Alzheimer's disease‐related dementias conference. Neurology. 2014;83(9):851‐860. doi:10.1212/WNL.0000000000000733 PMC4155046 25080517

[7] McKhann GM , Knopman DS , Chertkow H , et al. The diagnosis of dementia due to Alzheimer's disease: recommendations from the National Institute on Aging‐Alzheimer's Association Workgroups. Alzheimers Dement. 2011;7(3):263‐269. doi:10.1016/j.jalz.2011.03.005 PMC3312024 21514250

[8] Prince M , Wimo A , Guerchet M , et al. World Alzheimer report 2015: The global impact of dementia. Alzheimer's Disease International; 2015. Accessed August 14, 2026. https://www.alzint.org/u/WorldAlzheimerReport2015.pdf

[9] Russ TC , Batty GD , Hearnshaw GF , Fenton C , Starr JM . Geographical variation in dementia: systematic review with meta‐analysis. Int J Epidemiol. 2012;41(4):1012‐1032. doi:10.1093/ije/dys103 PMC3429875 22798662

[10] Cook CJ , Fletcher JM . Can education rescue genetic liability for cognitive decline?. Soc Sci Med. 2015;127:159‐170. doi:10.1016/j.socscimed.2014.06.049 PMC4286526 25074513

[11] Livingston G , Huntley J , Liu KY , et al. Dementia prevention, intervention, and care: 2024 report of the Lancet standing Commission. Lancet. 2024;404(10452):572‐628. doi:10.1016/S0140-6736(24)01296-0 39096926

[12] Baker LD , Espeland MA , Whitmer RA , et al. Structured vs self‐guided multidomain lifestyle interventions for global cognitive function: the US POINTER randomized clinical trial. JAMA. 2025;334(8):681‐691. doi:10.1001/jama.2025.12923 PMC12305445 40720610

[13] Goff DC Jr , Lloyd‐Jones DM , Bennett G , et al. 2013 ACC/AHA guideline on the assessment of cardiovascular risk: a report of the American College of Cardiology/American Heart Association Task Force on Practice Guidelines. Circulation. 2014;129(25):49‐73. doi:10.1161/01.cir.0000437741.48606.98 24222018

[14] Wild CP . Complementing the genome with an “exposome”: the outstanding challenge of environmental exposure measurement in molecular epidemiology. Cancer Epidemiol Biomarkers Prev. 2005;14(8):1847‐1850. doi:10.1158/1055-9965.EPI-05-0456 16103423

[15] Wild CP . The exposome: from concept to utility. Int J Epidemiol. 2012;41(1):24‐32. doi:10.1093/ije/dyr236 22296988

[16] Maitre L , Bustamante M , Hernández‐Ferrer C , et al. Multi‐omics signatures of the human early life exposome. Nat Commun. 2022;13(1):7024. doi:10.1038/s41467-022-34422-2 PMC9678903 36411288

[17] Finch CE , Kulminski AM . The Alzheimer's disease exposome. Alzheimers Dement. 2019;15(9):1123‐1132. doi:10.1016/j.jalz.2019.06.3914 PMC6788638 31519494

[18] Babulal GM . Neighborhoods, networks, and neurodegeneration: a call for population‐level policy and advancing the exposome. J Am Geriatr Soc. 2025;73(2):340‐342. doi:10.1111/jgs.18704 PMC11828669 39760328

[19] Power MC , Adar SD , Yanosky JD , Weuve J . Exposure to air pollution as a potential contributor to cognitive function, cognitive decline, brain imaging, and dementia: a systematic review of epidemiologic research. Neurotoxicology. 2016;56:235‐253. doi:10.1016/j.neuro.2016.06.004 PMC5048530 27328897

[20] Paul KC , Haan M , Mayeda ER , Ritz BR . Ambient air pollution, noise, and late‐life cognitive decline and dementia risk. Annu Rev Public Health. 2019;40:203‐220. doi:10.1146/annurev-publhealth-040218-044058 PMC6544148 30935305

[21] Cui B , Li K . Chronic noise exposure and Alzheimer disease: is there an etiological association?. Med Hypotheses. 2013;81(4):623‐626. doi:10.1016/j.mehy.2013.06.007 23899629

[22] Meng L , Zhang Y , Zhang S , et al. Chronic noise exposure and risk of dementia: a systematic review and dose‐response meta‐analysis. Front Aging Neurosci. 2022;14:925120. doi:10.3389/fnagi.2022.925120 PMC9251202 35795699

[23] Besser LM , Chang L‐C , Evenson KR , et al. Associations between neighborhood park access and longitudinal change in cognition in older adults: the multi‐ethnic study of atherosclerosis. J Alzheimers Dis. 2021;82(1):221‐233. doi:10.3233/JAD-210370 PMC8579707 34024841

[24] Zagnoli F , Filippini T , Jimenez MP , Wise LA , Hatch EE , Vinceti M . Is greenness associated with dementia? A systematic review and dose‐response meta‐analysis. Curr Environ Health Rep. 2022;9(4):574‐590. doi:10.1007/s40572-022-00365-5 PMC9729322 35857243

[25] Cherrie MPC , Shortt NK , Mitchell RJ , et al. Green space and cognitive ageing: a retrospective life course analysis in the Lothian Birth Cohort 1936. Soc Sci Med. 2018;196:56‐65. doi:10.1016/j.socscimed.2017.10.038 29128786

[26] Slawsky ED , Hajat A , Rhew IC , et al. Neighborhood greenspace exposure as a protective factor in dementia risk among US adults 75 years or older: a cohort study. Environ Health. 2022;21(1):14. doi:10.1186/s12940-022-00830-6 PMC8760791 35033073

[27] Boone CG , Buckley GL , Grove JM , Sister C . Parks and people: an environmental justice inquiry in Baltimore, Maryland. Ann Assoc Am Geogr. 2009;99(4):767‐787. doi:10.1080/00045600903102949

[28] Duncan DT , Kawachi I , White K , Williams DR . The geography of recreational open space: influence of neighborhood racial composition and neighborhood poverty. J Urban Health. 2013;90(4):618‐631. doi:10.1007/s11524-012-9770-y PMC3732687 23099625

[29] Karska J , Kowalski S , Gładka A , et al. Artificial light and neurodegeneration: does light pollution impact the development of Alzheimer's disease?. GeroScience. 2024;46(1):87‐97. doi:10.1007/s11357-023-00932-0 PMC10828315 37733222

[30] Irwin MR , Vitiello MV . Implications of sleep disturbance and inflammation for Alzheimer's disease dementia. Lancet Neurol. 2019;18(3):296‐306. doi:10.1016/S1474-4422(18)30450-2 30661858

[31] Dintica CS , Bahorik A , Xia F , Kind AJH , Yaffe K . Dementia risk and disadvantaged neighborhoods. JAMA Neurol. 2023;80(9):903‐909. doi:10.1001/jamaneurol.2023.2120 PMC10357362 37464954

[32] Santibez M , Bolumar F , Garca AM . Occupational risk factors in Alzheimer's disease: a review assessing the quality of published epidemiological studies. Occup Environ Med. 2007;64(11):723‐732. doi:10.1136/oem.2006.028209 PMC2078415 17525096

[33] Hinton L , Tran D , Peak K , Meyer OL , Quiñones AR . Mapping racial and ethnic healthcare disparities for persons living with dementia: a scoping review. Alzheimers Dement. 2024;20(4):3000‐3020. doi:10.1002/alz.13612 PMC11032576 38265164

[34] Ioannidis JP . Why most published research findings are false. PLoS Med. 2005;2(8):e124. doi:10.1371/journal.pmed.0020124 PMC1182327 16060722

[35] Patel CJ , Ioannidis JPA . Studying the elusive environment in large scale. JAMA. 2014;311(21):2173‐2174. doi:10.1001/jama.2014.4129 PMC4110965 24893084

[36] Weuve J , Barnes LL , Mendes de Leon CF , et al. Cognitive aging in Black and White Americans: cognition, cognitive decline, and incidence of Alzheimer disease dementia. Epidemiology. 2018;29(1):151‐159. doi:10.1097/EDE.0000000000000747 PMC5718953 28863046

[37] Froehlich TE , Bogardus ST Jr , Inouye SK . Dementia and race: are there differences between African Americans and Caucasians?. J Am Geriatr Soc. 2001;49(4):477‐484. doi:10.1046/j.1532-5415.2001.49096.x 11347796

[38] Matthews KA , Xu W , Gaglioti AH , et al. Racial and ethnic estimates of Alzheimer's disease and related dementias in the United States (2015‐2060) in adults aged ≥65 years. Alzheimers Dement. 2019;15(1):17‐24. doi:10.1016/j.jalz.2018.06.3063 PMC6333531 30243772

[39] Rotstein A . Sex differences in late‐life cognition: a Psychometric network analysis of well‐functioning older adults. Am J Geriatr Psychiatry. 2025;33(7):781‐792. doi:10.1016/j.jagp.2024.12.002 39765444

[40] Babulal GM , Quiroz YT , Albensi BC , et al. Perspectives on ethnic and racial disparities in Alzheimer's disease and related dementias: update and areas of immediate need. Alzheimers Dement. 2019;15(2):292‐312. doi:10.1016/j.jalz.2018.09.009 PMC6368893 30555031

[41] Norton S , Matthews FE , Barnes DE , Yaffe K , Brayne C . Potential for primary prevention of Alzheimer's disease: an analysis of population‐based data. Lancet Neurol. 2014;13(8):788‐794. doi:10.1016/S1474-4422(14)70136-X 25030513

[42] Vermeulen R , Schymanski EL , Barabási A‐L , Miller GW . The exposome and health: where chemistry meets biology. Science. 2020;367:392‐396. doi:10.1126/science.aay3164 PMC7227413 31974245

[43] Patel CJ , Bhattacharya J , Butte AJ . An Environment‐Wide Association Study (EWAS) on type 2 diabetes mellitus. PLoS One. 2010;5(5):e10746. doi:10.1371/journal.pone.0010746 PMC2873978 20505766

[44] Chung MK , House JS , Akhtari FS , et al. Decoding the exposome: data science methodologies and implications in exposome‐wide association studies (ExWASs). 2024;4:osae001. doi:10.1093/exposome/osae001 PMC10857773 38344436

[45] Wan M , Simonin EM , Johnson MM , et al. Exposomics: a review of methodologies, applications, and future directions in molecular medicine. EMBO Mol Med. 2025;17(4):599‐608. doi:10.1038/s44321-025-00191-w PMC11982546 39870881

[46] Park SK , Tao Y , Meeker JD , Harlow SD , Mukherjee B . Environmental risk score as a new tool to examine multi‐pollutants in epidemiologic research: an example from the NHANES study using serum lipid levels. PLoS One. 2014;9(6):e98632. doi:10.1371/journal.pone.0098632 PMC4047033 24901996

[47] He Y , Lakhani CM , Rasooly D , Manrai AK , Tzoulaki I , Patel CJ . Comparisons of polyexposure, polygenic, and clinical risk scores in risk prediction of type 2 diabetes. Diabetes Care. 2021;44(4):935‐943. doi:10.2337/dc20-2049 PMC7985424 33563654

[48] He Y , Qian DC , Diao JA , et al. Prediction and stratification of longitudinal risk for chronic obstructive pulmonary disease across smoking behaviors. Nat Commun. 2023;14:8297. doi:10.1038/s41467-023-44047-8 PMC10721891 38097585

[49] Guimbaud J‐B , Siskos AP , Sakhi AK , et al. Machine learning‐based health environmental‐clinical risk scores in European children. Commun Med (Lond). 2024;4(1):98. doi:10.1038/s43856-024-00513-y PMC11116423 38783062

[50] Sudlow C , Gallacher J , Allen N , et al. UK Biobank: an open access resource for identifying the causes of a wide range of complex diseases of middle and old age. PLoS Med. 2015;12(3):e1001779. doi:10.1371/journal.pmed.1001779 PMC4380465 25826379

[51] Chen T , Guestrin C . XGBoost: A Scalable Tree Boosting System. Proceedings of the 22nd ACM SIGKDD International Conference on Knowledge Discovery and Data Mining. ACM; 2016:785‐794. doi:10.1145/2939672.2939785

[52] Lundberg SM , Lee SI . A unified approach to interpreting model predictions. In: Guyon I , von Luxburg U , Bengio S , et al., eds. Advances in Neural Information Processing Systems 30. Curran Associates; 2017:4765‐4774. doi:10.48550/arXiv.1702.08608

[53] Maher RL , Hanlon J , Hajjar ER . Clinical consequences of polypharmacy in elderly. Expert Opin Drug Saf. 2014;13(1):57‐65. doi:10.1517/14740338.2013.827660 PMC3864987 24073682

[54] Clague F , Mercer SW , McLean G , Reynish E , Guthrie B . Comorbidity and polypharmacy in people with dementia: insights from a large, population‐based cross‐sectional analysis of primary care data. Age Ageing. 2017;46(1):33‐39. doi:10.1093/ageing/afw176 28181629

[55] Liu K . Circadian rhythm disruption and sleep disorders in Alzheimer's disease: mechanistic insights and therapeutic potentials. Neuro Endocrinol Lett. 2025;46(2):59‐69. 40929704

[56] Nguyen Ho PT , Hoepel SJW , Rodriguez‐Ayllon M , et al. Sleep, 24‐hour activity rhythms, and subsequent amyloid‐β pathology. JAMA Neurol. 2024;81(8):824‐834. doi:10.1001/jamaneurol.2024.1755 PMC11197458 38913396

[57] Shokri‐Kojori E , Wang G‐J , Wiers CE , et al. β‐Amyloid accumulation in the human brain after one night of sleep deprivation. Proc Natl Acad Sci U S A. 2018;24(17):4483‐4488. doi:10.1073/pnas.1721694115 PMC5924922 29632177

[58] Erickson KI , Voss MW , Prakash RS , et al. Exercise training increases size of hippocampus and improves memory. Proc Natl Acad Sci U S A. 2011;108(7):3017‐3022. doi:10.1073/pnas.1015950108 PMC3041121 21282661

[59] Hamer M , Chida Y . Physical activity and risk of neurodegenerative disease: a systematic review of prospective evidence. Psychol Med. 2009;39(1):3‐11. doi:10.1017/S0033291708003681 18570697

[60] Stern Y . Cognitive reserve in ageing and Alzheimer's disease. Lancet Neurol. 2012;11(11):1006‐1012. doi:10.1016/S1474-4422(12)70191-6 PMC3507991 23079557

[61] Fratiglioni L , Paillard‐Borg S , Winblad B . An active and socially integrated lifestyle in late life might protect against dementia. Lancet Neurol. 2004;3(6):343‐353. doi:10.1016/S1474-4422(04)00767-7 15157849

[62] Yuan T , Liu K , Liang L , et al. Associations between internet use, social participation, and cognitive function among middle‐aged and older adults. Npj Mental Health Res. 2025;4:47. doi:10.1038/s44184-025-00162-6 PMC12485158 41028880

[63] Benge JF , Scullin MK . A meta‐analysis of technology use and cognitive aging. Nat Hum Behav. 2025;9:1405‐1419. doi:10.1038/s41562-025-02159-9 PMC12333551 40229575

[64] Beydoun MA , Hu Yi‐H , Li Z , et al. Alzheimer's disease polygenic risk's association with all‐cause dementia through the plasma metabolome in the UK Biobank study. Geroscience. 2025;47(6):7023‐7042. doi:10.1007/s11357-025-01724-4 PMC12638481 40588577

[65] Li K , Shacham E , Brown D , et al. Association of environmental exposome and cognitive function among older adults with and without preclinical Alzheimer's disease. Alzheimers Dement. 2025;21(1):e70373. doi:10.1002/alz.70373 PMC12170949 40524437

[66] Srivastava SB . Polypharmacy, unintended consequences, and impact of lifestyle medicine. Am J Lifestyle Med. 2024;18(1):54‐57. doi:10.1177/15598276231207302 PMC11339771 39184275

[67] Viña J , Lloret A . Why women have more Alzheimer's disease than men: gender and mitochondrial toxicity of amyloid‐β peptide. J Alzheimers Dis. 2010;20(S2):S527‐S533. doi:10.3233/JAD-2010-100501 20442496

[68] Ngandu T , Lehtisalo J , Solomon A , et al. A 2 year multidomain intervention of diet, exercise, cognitive training, and vascular risk monitoring versus control to prevent cognitive decline in at‐risk elderly people (FINGER): a randomised controlled trial. Lancet. 2015;385(9984):2255‐2263. doi:10.1016/S0140-6736(15)60461-5 25771249

[69] Jack CR Jr , DA B , Blennow K , et al. NIA‐AA research framework: toward a biological definition of Alzheimer's disease. Alzheimers Dement. 2018;14(4):535‐562. doi:10.1016/j.jalz.2018.02.018 PMC5958625 29653606

[70] Fry A , Littlejohns TJ , Sudlow C , et al. Comparison of sociodemographic and health‐related characteristics of UK Biobank participants with those of the general population. Am J Epidemiol. 2017;186(9):1026‐1034. doi:10.1093/aje/kwx246 PMC5860371 28641372

[71] Ellis RJ , Airaud A , Jasodanand VH , et al. Prognostic performance across Alzheimer's biomarkers, multi‐modal physiological measures, and clinical history in asymptomatic individuals. medRxiv [Preprint]. 2025. doi:10.1101/2025.11.19.25340533

