# Supplementary material for: Exposome‐wide association study and predictive risk score for incident Alzheimer's disease and related dementias
Source: Alzheimers Dement. 2026 Sep 29;22(10):e71842. doi: 10.1002/alz.71842 (PMC13624529; doi:10.1002/alz.71842)
Supplement: Supplementary file 2 [file ALZ-22-e71842-s002.docx]

**Supplementary Figure 1.** Flowchart of the participants selection

**Supplementary Figure 2.** Flowchart of the exposure variables selection

**Supplementary Figure 3.** Correlations heatmap and clustering dendrogram of significant exposures associated with ADRD

**Supplementary Figure 4.** Correlations heatmap and clustering dendrogram of significant exposures associated with AD

**Supplementary Figure 5.** Distribution of Exposomic Risk Score (ERS) in the full sample

**Supplementary Figure 6.** Kaplan-Meier survival curves stratified by genetic risk groups

**Supplementary Table 1.** Definitions for Alzheimer’s disease and related dementias

**Supplementary Table 2.** Exposome variables processing

**Supplementary Table 3.** Baseline characteristics of the studied population

**Supplementary Table 4.** Baseline characteristics of the studied population in derivation set

**Supplementary Table 5.** Baseline characteristics of the studied population in replication set

**Supplementary Table 6.** Baseline characteristics of the studied population by subgroups

**Supplementary Table 7.** Associations between 193 exposures and ADRD in ExWAS

**Supplementary Table 8.** Associations between 193 exposures and AD in ExWAS

**Supplementary Table 9.** Time-dependent effects of the exposures on incident ADRD

**Supplementary Table 10.** Associations between 193 exposures and ADRD in subgroups (by sex, age, genetics, and ethnicity)

**Supplementary Table 11.** Associations between 193 exposures and AD in subgroups (by sex, age, genetics, and ethnicity)

**Supplementary Table 12.** Cross-validation performance of XGBoost models across subgroups for ADRD and AD

**Supplementary Table 13.** Model performance metrics for ADRD and AD prediction

**Supplementary Figure 1. Flowchart of the participants selection**

(a) Individuals in the UK Biobank cohort were excluded if they had reported ADRD at baseline. The remaining participants were classified based on their first reported years of ADRD or AD after baseline. (b) Left: distribution of observation times for the derived study population. Middle: distribution of first reported years of ADRD after baseline. Right: distribution of first reported years of AD after baseline. Abbreviations: ADRD = Alzheimer’s Disease and Related Dementias, AD = Alzheimer’s Disease.

**Supplementary Figure 2. Flowchart of the exposure variables selection**

**Supplementary Figure 3. Correlations heatmap and clustering dendrogram of significant exposures associated with ADRD**

Top panel presents the Spearman rank-order correlations, and bottom panel shows the results of hierarchical clustering. The horizontal dash line was the cutoff of clusters, and only one predictor was chosen within each cluster. Abbreviations: ADRD = Alzheimer’s Disease and Related Dementias

**Supplementary Figure 4. Correlations heatmap and clustering dendrogram of significant exposures associated with AD**

Top panel presents the Spearman rank-order correlations, and bottom panel shows the results of hierarchical clustering. The horizontal dash line was the cutoff of clusters, and only one predictor was chosen within each cluster. Abbreviations: AD = Alzheimer’s Disease

**Supplementary Figure 5. Distribution of Exposomic Risk Score (ERS) in the full sample**

(a) ADRD. (b) AD. Left panel presents overall histogram, and right panel presents density plot stratified by case-control status. ADRD and AD Exposomic Risk Score (ERS) differently distributed between cases and control groups. Differences between the case and control groups were assessed using a two-sided Wilcoxon rank-sum test. Abbreviations: ADRD = Alzheimer’s Disease and Related Dementias, AD = Alzheimer’s Disease.

**Supplementary Figure 6.** **Kaplan-Meier survival curves stratified by genetic risk groups**

(a) ADRD. (b) AD. Genetic risks were evaluated using the *APOE* genotype, with the presence of ε4 indicating a high genetic risk, ε3ε3 an intermediate risk and others a low risk.

**Supplementary Table 1. Definitions for Alzheimer’s disease and related dementias**

| **ADRD** | | | |
| --- | --- | --- | --- |
|  | **Field ID** | **Description** | **Matching Code** |
| Algorithmically-defined outcomes | 42018 | Date of all cause dementia report |  |
|  | 42019 | Source of all cause dementia report | 1;2;11;12;21;22 |
|  | 42020 | Date of alzheimer's disease report |  |
|  | 42021 | Source of alzheimer's disease report | 1;2;11;12;21;22 |
|  | 42022 | Date of vascular dementia report |  |
|  | 42023 | Source of vascular dementia report | 1;2;11;12;21;22 |
|  | 42024 | Date of frontotemporal dementia report |  |
|  | 42025 | Source of frontotemporal dementia report | 1;2;11;12;21;22 |
| First occurrences | 131036 | Date G30 first reported (alzheimer's disease) |  |
|  | 131037 | Source of report of G30 (alzheimer's disease) | 20;21;30;31;40;41 |
|  | 130836 | Date F00 first reported (dementia in alzheimer's disease) |  |
|  | 130837 | Source of report of F00 (dementia in alzheimer's disease) | 20;21;30;31;40;41 |
|  | 130838 | Date F01 first reported (vascular dementia) |  |
|  | 130839 | Source of report of F01 (vascular dementia) | 20;21;30;31;40;41 |
|  | 130840 | Date F02 first reported (dementia in other diseases classified elsewhere) |  |
|  | 130841 | Source of report of F02 (dementia in other diseases classified elsewhere) | 20;21;30;31;40;41 |
|  | 130842 | Date F03 first reported (unspecified dementia) |  |
|  | 130843 | Source of report of F03 (unspecified dementia) | 20;21;30;31;40;41 |
| Death register | 40001 | Death register-Underlying (primary) cause of death: ICD10 | F01.1;F01.2;F01.9;F03;G30.0;G30.1;G30.9 |
|  | 40002 | Death register-Contributory (secondary) causes of death: ICD10 | F01.1;F01.9;F03;G30.0;G30.1;G30.9 |
| Hospital inpatient | 41280 | Date of first in-patient diagnosis - ICD10 |  |
|  | 41270 | Diagnoses - ICD10 | F00.0;F00.1;F00.2;F00.9;F01.0;F01.1;F01.2;F01.3;F01.8;F01.9;F02.0;F02.1;F02.2;F02.3;F02.4;F02.8;F03;G30.0;G30.1;G30.8;G30.9 |
| **AD** | | | |
|  | **Field ID** | **Description** | **Matching Code** |
| Algorithmically-defined outcomes | 42020 | Date of alzheimer's disease report |  |
|  | 42021 | Source of alzheimer's disease report | 1;2;11;12;21;22 |
| First occurrences | 131036 | Date G30 first reported (alzheimer's disease) |  |
|  | 131037 | Source of report of G30 (alzheimer's disease) | 20;21;30;31;40;41 |
|  | 130836 | Date F00 first reported (dementia in alzheimer's disease) |  |
|  | 130837 | Source of report of F00 (dementia in alzheimer's disease) | 20;21;30;31;40;41 |
| Death register | 40001 | Death register-Underlying (primary) cause of death: ICD10 | G30.0;G30.1;G30.9 |
|  | 40002 | Death register-Contributory (secondary) causes of death: ICD10 | G30.0;G30.1;G30.9 |
| Hospital inpatient | 41280 | Date of first in-patient diagnosis - ICD10 |  |
|  | 41270 | Diagnoses - ICD10 | F00;F00.0;F00.1;F00.2;F00.9;G30;G30.0;G30.1;G30.8;G30.9 |

**Notes:** Please refer to the webpage of UK-Biobank for detailed information (https://www.ukbiobank.ac.uk). Dementia with Lewy bodies is not separately ascertainable in the UK Biobank and is captured within unspecified dementia (F03). Codes for alcohol-related dementia and Wernicke-Korsakoff syndrome (F10.6, G31.2) were not included in the outcome definition. Abbreviations: ADRD= Alzheimer’s disease and related dementias, AD=Alzheimer’s disease, ICD= International Classification of Diseases

**Supplementary Table 2. Exposome variables processing**

| **Variable** | **Code** | **Description** | **Value type** | **Field ID** | **Category** | **Coding notes** | **Original N (Missing Rate %)** |
| --- | --- | --- | --- | --- | --- | --- | --- |
| Friend/family visits frequently | p1031 | Frequency of friend/family visits | Ordinal categorical | 1031 | Social | Coding: 1=No friends/family outside household/Never or almost never; 2=Once every few months; 3=About once a month; 4=About once a week; 5=2-4 times a week; 6=Almost daily | 494,476 (1.58) |
| Relatively thinner at 10 years old; | p1687_1 | Comparative body size at age 10 | Categorical | 1687 | Social | Dummy coding: 0=About average; 1=Thinner | 491,359 (2.2) |
| Relatively plumper at 10 years old | p1687_2 | Comparative body size at age 10 | Categorical | 1687 | Social | Dummy coding: 0=About average; 1=Plumper | 491,359 (2.2) |
| Relatively shorter at 10 years old | p1697_1 | Comparative height size at age 10 | Categorical | 1697 | Social | Dummy coding: 0=About average; 1=Shorter | 492,007 (2.07) |
| Relatively taller at 10 years old | p1697_2 | Comparative height size at age 10 | Categorical | 1697 | Social | Dummy coding: 0=About average; 1=Taller | 492,007 (2.07) |
| Adopted as a child | p1767 | Adopted as a child | Categorical | 1767 | Social | Coding: 0=No; 1=Yes | 499,927 (0.49) |
| Mood swings | p1920 | Mood swings | Categorical | 1920 | Social | Coding: 0=No; 1=Yes | 488,102 (2.85) |
| Miserableness | p1930 | Miserableness | Categorical | 1930 | Social | Coding: 0=No; 1=Yes | 492,017 (2.07) |
| Irritability | p1940 | Irritability | Categorical | 1940 | Social | Coding: 0=No; 1=Yes | 477,886 (4.88) |
| Sensitivity/hurt feelings | p1950 | Sensitivity / hurt feelings | Categorical | 1950 | Social | Coding: 0=No; 1=Yes | 486,083 (3.25) |
| Fed-up feelings | p1960 | Fed-up feelings | Categorical | 1960 | Social | Coding: 0=No; 1=Yes | 489,846 (2.5) |
| Nervous feelings | p1970 | Nervous feelings | Categorical | 1970 | Social | Coding: 0=No; 1=Yes | 487,480 (2.97) |
| Worrier/anxious feelings | p1980 | Worrier / anxious feelings | Categorical | 1980 | Social | Coding: 0=No; 1=Yes | 487,527 (2.96) |
| Tense/'highly strung' | p1990 | Tense / 'highly strung' | Categorical | 1990 | Social | Coding: 0=No; 1=Yes | 482,579 (3.95) |
| Worry too long after embarrassment | p2000 | Worry too long after embarrassment | Categorical | 2000 | Social | Coding: 0=No; 1=Yes | 480,074 (4.45) |
| Suffer from 'nerves' | p2010 | Suffer from 'nerves' | Categorical | 2010 | Social | Coding: 0=No; 1=Yes | 481,783 (4.11) |
| Neuroticism score | p20127 | Neuroticism score | Continuous | 1608 | Social | Standardized (mean = 0, SD = 1) | 401,496 (20.09) |
| Loneliness, isolation | p2020 | Loneliness, isolation | Categorical | 2020 | Social | Coding: 0=No; 1=Yes | 492,539 (1.96) |
| Guilty feelings | p2030 | Guilty feelings | Categorical | 2030 | Social | Coding: 0=No; 1=Yes | 487,014 (3.06) |
| Risk taking | p2040 | Risk taking | Categorical | 2040 | Social | Coding: 0=No; 1=Yes | 481,972 (4.07) |
| Feels depressed mood frequently | p2050 | Frequency of depressed mood in last 2 weeks | Ordinal categorical | 2050 | Social | Coding: 1=Not at all; 2=Several days; 3=More than half the days; 4=Nearly every day | 478,237 (4.81) |
| Feels unenthusiasm/disinterest frequently | p2060 | Frequency of unenthusiasm / disinterest in last 2 weeks | Ordinal categorical | 2060 | Social | Coding: 1=Not at all; 2=Several days; 3=More than half the days; 4=Nearly every day | 482,601 (3.94) |
| Feels tenseness/restlessness frequently | p2070 | Frequency of tenseness / restlessness in last 2 weeks | Ordinal categorical | 2070 | Social | Coding: 1=Not at all; 2=Several days; 3=More than half the days; 4=Nearly every day | 480,215 (4.42) |
| Feels tiredness/lethargy frequently | p2080 | Frequency of tiredness / lethargy in last 2 weeks | Ordinal categorical | 2080 | Social | Coding: 1=Not at all; 2=Several days; 3=More than half the days; 4=Nearly every day | 485,154 (3.43) |
| Able to confide frequently | p2110 | Able to confide | Ordinal categorical | 2110 | Social | Coding: 0=Never or almost never; 1=Once every few months; 2=About once a month; 3=About once a week; 4=2-4 times a week; 5=Almost daily | 483,535 (3.76) |
| Lower education | p6138 | Qualifications | Categorical | 6138 | Social | Turn data into binary classification. Dummy coding: 0=Other professional qualifications eg: nursing, teaching/College or University degree; 1= A levels/AS levels or equivalent/O levels/GCSEs or equivalent/CSEs or equivalent/NVQ or HND or HNC or equivalent/None of the above | 492,277 (2.02) |
| Lives with partner | p6141 | How are people in household related to participant | Categorical | 6141 | Social | Turn data into binary classification. Dummy coding: 0=Son and/or daughter (include step-children)/Brother and/or sister/Mother and/or father/Grandparent/Grandchild/Other related/Other unrelated; 1=Husband, wife or partner | 500,270 (0.43) |
| Unemployed | p6142 | Current employment status | Categorical | 6142 | Social | Turn data into binary classification. Dummy coding: 0=In paid employment or self-employed/Retired/Doing unpaid or voluntary work/Full or part-time student; 1=Looking after home and/or family/Unable to work because of sickness or disability/Unemployed | 496,658 (1.14) |
| Own serious illness or injury | p6145_1 | Serious illness, injury or assault to yourself | Categorical | 6145 | Social | Dummy coded from array variables. Dummy coding: 0=No; 1=Yes | 494,189 (1.64) |
| Relative’s serious illness or injury | p6145_2 | Serious illness, injury or assault of a close relative | Categorical | 6145 | Social | Dummy coded from array variables. Dummy coding: 0=No; 1=Yes | 494,189 (1.64) |
| Death of a close relative | p6145_3 | Death of a close relative | Categorical | 6145 | Social | Dummy coded from array variables. Dummy coding: 0=No; 1=Yes | 494,189 (1.64) |
| Death of a spouse or partner | p6145_4 | Death of a spouse or partner | Categorical | 6145 | Social | Dummy coded from array variables. Dummy coding: 0=No; 1=Yes | 494,189 (1.64) |
| Marital separation/divorce | p6145_5 | Marital separation/divorce | Categorical | 6145 | Social | Dummy coded from array variables. Dummy coding: 0=No; 1=Yes | 494,189 (1.64) |
| Financial difficulties | p6145_6 | Financial difficulties | Categorical | 6145 | Social | Dummy coded from array variables. Dummy coding: 0=No; 1=Yes | 494,189 (1.64) |
| Disability | p6146 | Attendance/disability/mobility allowance | Categorical | 6146 | Social | Turn data into binary classification. Dummy coding: 0=None of the above; 1=Attendance allowance/Disability living allowance/Blue badge | 496,877 (1.10) |
| Lives in rent accommodation versus own | p680 | Own or rent accommodation lived in | Categorical | 680 | Social | Dummy coding: 0=Own outright (by you or someone in your household)/Own with a mortgage; 1=Rent - from local authority, local council, housing association/Rent - from private landlord or letting agency/Pay part rent and part mortgage (shared ownership)/Live in accommodation rent free | 493,933 (1.69) |
| Length of residence at current address | p699 | Length of time at current address | Continuous | 699 | Social | Standardized (mean = 0, SD = 1) | 499,736 (0.53) |
| Number in household | p709 | Number in household | Continuous | 709 | Social | Standardized (mean = 0, SD = 1) | 497,860 (0.91) |
| Number of vehicles in household | p728 | Number of vehicles in household | Ordinal categorical | 728 | Social | Coding: 1=None; 2=One; 3=Two; 4=Three; 5=Four or more | 497,774 (0.92) |
| Total household income | p738 | Average total household income before tax | Ordinal categorical | 738 | Social | Coding: 1=Less than 18,000; 2=18,000 to 30,999; 3=31,000 to 51,999; 4=52,000 to 100,000; 5=Greater than 100,000 | 425,265 (15.35) |
| Time spent outdoors (summer) | p1050 | Time spend outdoors in summer | Continuous | 1050 | Physical | Standardized (mean = 0, SD = 1) | 468,767 (6.70) |
| Time spent outdoors (winter) | p1060 | Time spent outdoors in winter | Continuous | 1060 | Physical | Standardized (mean = 0, SD = 1) | 468559 (6.74) |
| Childhood sunburn occurred frequently | p1737 | Childhood sunburn occasions | Continuous | 1737 | Physical | Standardized (mean = 0, SD = 1) | 375,663 (25.23) |
| Degree of facial ageing | p1757 | Facial ageing | Ordinal categorical | 1757 | Physical | Coding: 0=Younger than you are; 1=About your age; 2=Older than you are | 457,129 (9.01) |
| Uses sun or UV protection frequently | p2267 | Use of sun/uv protection | Ordinal categorical | 2267 | Physical | Coding: 0=Do not go out in sunshine; 1=Never/rarely; 2=Sometimes; 3=Most of the time; 4=Always | 496,539 (1.17) |
| Uses solarium/sunlamp frequently | p2277 | Frequency of solarium/sunlamp use | Continuous | 2277 | Physical | Standardized (mean = 0, SD = 1) | 491,911 (2.09) |
| Traffic intensity on nearest road | p24009 | Traffic intensity on the nearest road | Continuous | 24009 | Physical | Standardized (mean = 0, SD = 1) | 495,031 (1.47) |
| Inverse distance to road | p24010 | Inverse distance to the nearest road | Continuous | 24010 | Physical | Standardized (mean = 0, SD = 1) | 495,031 (1.47) |
| Traffic intensity on nearest major road | p24011 | Traffic intensity on the nearest major road | Continuous | 24011 | Physical | Standardized (mean = 0, SD = 1) | 495,031 (1.47) |
| Inverse distance to major road | p24012 | Inverse distance to the nearest major road | Continuous | 24012 | Physical | Standardized (mean = 0, SD = 1) | 495,031 (1.47) |
| Close to major road | p24014 | Close to major road | Categorical | 24014 | Physical | Coding: 0=No; 1=Yes | 495,031 (1.47) |
| Average noise level (daytime) | p24020 | Average daytime sound level of noise pollution | Continuous | 24020 | Physical | Standardized (mean = 0, SD = 1) | 495,031 (1.47) |
| Average noise level (evening) | p24021 | Average evening sound level of noise pollution | Continuous | 24011 | Physical | Standardized (mean = 0, SD = 1) | 495,031 (1.47) |
| Average noise level (nighttime) | p24022 | Average night-time sound level of noise pollution | Continuous | 24022 | Physical | Standardized (mean = 0, SD = 1) | 495,031 (1.47) |
| Lives in a flat versus house | p670_1 | Type of accommodation lived in | Categorical | 670 | Physical | Dummy coding: 0=A house or bungalow; 1=A flat, maisonette or apartment | 501,035 (0.27) |
| Lives in mobile structure versus house | p670_2 | Type of accommodation lived in | Categorical | 670 | Physical | Dummy coding: 0=A house or bungalow; 1=Mobile or temporary structure (i.e. caravan)/Sheltered accommodation/Care home/None of the above | 501,035 (0.27) |
| Healthy diet | p100_1 | Healthy_diet_level | Categorical | 1289, 1299, 1309, 1319, 1329, 1339, 1438, 1448, 1458, 1468, 1369, 1379, 1389, 1349. | Lifestyle | Dummy coding: 0 = diet score < 4, 1 = diet score ≥ 4. For further details on the derivation and coding of this variable, see the *Notes* under the Table. | 501,355 (0.21) |
| Time spent watching television | p1070 | Time spent watching television (TV) | Continuous | 1070 | Lifestyle | Standardized (mean = 0, SD = 1) | 496,994 (1.08) |
| Time spent using computer | p1080 | Time spent using computer | Continuous | 1080 | Lifestyle | Standardized (mean = 0, SD = 1) | 493,045 (1.86) |
| Time spent driving | p1090 | Time spent driving | Continuous | 1090 | Lifestyle | Standardized (mean = 0, SD = 1) | 492,476 (1.98) |
| Drives above motorway speed limit frequently | p1100 | Drive faster than motorway speed limit | Ordinal categorical | 1100 | Lifestyle | Coding: 1=Never/rarely/Do not drive on the motorway; 2=Sometimes; 3=Often; 4=Most of the time | 485,911 (3.28) |
| Length of mobile phone use | p1110 | Length of mobile phone use | Ordinal categorical | 1110 | Lifestyle | Coding: 1=Never used mobile phone at least once per week; 2=One year or less; 3=Two to four years; 4=Five to eight years; 5=More than eight years | 494,361 (1.60) |
| Weekly mobile phone usage | p1120 | Weekly usage of mobile phone in last 3 months | Ordinal categorical | 1120 | Lifestyle | Coding: 0=Less than 5mins; 1=5-29 mins; 2=30-59 mins; 3=1-3 hours; 4=4-6 hours; 5=More than 6 hours | 416,603 (17.08) |
| Uses hands-free or speakerphone frequently | p1130 | Hands-free device/speakerphone use with mobile phone in last 3 month | Ordinal categorical | 1130 | Lifestyle | Coding: 0=Never or almost never; 1=Less than half the time; 2=About half the time; 3=More than half the time; 4=Always or almost always | 418,399 (16.72) |
| Less frequent mobile phone use versus no difference | p1140_1 | Difference in mobile phone use compared to two years previously | Categorical | 1140 | Lifestyle | Dummy coding: 0=No; 1=Yes, use is now less frequent | 418,401 (16.72) |
| More frequent mobile phone use versus no difference | p1140_2 | Difference in mobile phone use compared to two years previously | Categorical | 1140 | Lifestyle | Dummy coding: 0=No; 1=Yes, use is now more frequent | 418,401 (16.72) |
| Didn't use a mobile phone versus no difference | p1140_3 | Difference in mobile phone use compared to two years previously | Categorical | 1140 | Lifestyle | Dummy coding: 0=No; 1=I didn't use a mobile phone two years ago | 418,401 (16.72) |
| Prefers left side of head for mobile phone use | p1150_1 | Usual side of head for mobile phone use | Categorical | 1150 | Lifestyle | Dummy coding: 0=Equally left and right; 1=Left | 419,806 (16.44) |
| Prefers right side of head for mobile phone use | p1150_2 | Usual side of head for mobile phone use | Categorical | 1150 | Lifestyle | Dummy coding: 0=Equally left and right; 1=Right | 419,806 (16.44) |
| Gets up more easily in the morning | p1170 | Getting up in morning | Ordinal categorical | 1170 | Lifestyle | Coding: 1=Not at all easy; 2=Not very easy; 3=Fairly easy; 4=Very easy | 496,734 (1.13) |
| More evening-type chronotype | p1180 | Morning/evening person (chronotype) | Ordinal categorical | 1180 | Lifestyle | Coding: 1=Definitely a 'morning' person; 2=More a 'morning' than 'evening' person; 3=More an 'evening' than a 'morning' person; 4=Definitely an 'evening' person | 444,095 (11.61) |
| Naps frequently | p1190 | Nap during day | Ordinal categorical | 1190 | Lifestyle | Coding: 1=Never/rarely; 2=Sometimes; 3=Usually | 500,514 (0.38) |
| Experiences insomnia frequently | p1200 | Sleeplessness / insomnia | Ordinal categorical | 1200 | Lifestyle | Coding: 1=Never/rarely; 2=Sometimes; 3=Usually | 500,904 (0.30) |
| Snoring | p1210 | Snoring | Categorical | 1210 | Lifestyle | Coding: 0=No; 1=Yes | 465,366 (7.37) |
| Daytime sleep frequency | p1220 | Daytime dozing / sleeping | Ordinal categorical | 1220 | Lifestyle | Coding: 0=Never/rarely; 1=Sometimes; 2=Often; 3=All of the time | 498,672 (0.74) |
| Current tobacco smoking frequency | p1239 | Current tobacco smoking | Ordinal categorical | 1239 | Lifestyle | Coding: 1=No; 2=Only occasionally; 3=Yes, on most or all days | 501,088 (0.26) |
| Past tobacco smoking frequency | p1249 | Past tobacco smoking | Ordinal categorical | 1249 | Lifestyle | Coding: 1=I have never smoked; 2=Just tried once or twice; 3=Smoked occasionally; 4=Smoked on most or all days | 460,294 (8.38) |
| Poultry intake | p1359 | Poultry intake | Ordinal categorical | 1359 | Lifestyle | Coding: 1=Never; 2=Less than once a week; 3=Once a week; 4=2-4 times a week; 5=5-6 times a week; 6=Once or more daily | 457,947 (8.85) |
| Cheese intake | p1408 | Cheese intake | Ordinal categorical | 1408 | Lifestyle | Coding: 1=Never; 2=Less than once a week; 3=Once a week; 4=2-4 times a week; 5=5-6 times a week; 6=Once or more daily | 487,956 (2.88) |
| Full-cream milk | p1418_1 | Milk type used | Categorical | 1418 | Lifestyle | Coding: 0=Never/rarely have milk; 1=Full cream | 500,882 (0.30) |
| Semi-skimmed milk | p1418_2 | Milk type used | Categorical | 1418 | Lifestyle | Coding: 0=Never/rarely have milk; 1=Semi-skimmed | 500,882 (0.30) |
| Skimmed milk | p1418_3 | Milk type used | Categorical | 1418 | Lifestyle | Coding: 0=Never/rarely have milk; 1=Skimmed | 500,882 (0.30) |
| Soya milk | p1418_4 | Milk type used | Categorical | 1418 | Lifestyle | Coding: 0=Never/rarely have milk; 1=Soya | 500,882 (0.30) |
| Other milk types | p1418_5 | Milk type used | Categorical | 1418 | Lifestyle | Coding: 0=Never/rarely have milk; 1=Other type of milk | 500,882 (0.30) |
| Butter/spreadable butter | p1428_1 | Spread type | Categorical | 1428 | Lifestyle | Coding: 0=Never/rarely use spread; 1=Butter/spreadable butter | 496,605 (1.16) |
| Flora Pro-Active spread | p1428_2 | Spread type | Categorical | 1428 | Lifestyle | Coding: 0=Never/rarely use spread; 1=Flora Pro-Active/Benecol | 496,605 (1.16) |
| Other spreads/margarine | p1428_3 | Spread type | Categorical | 1428 | Lifestyle | Coding: 0=Never/rarely use spread; 1=Other type of spread/margarine | 496,605 (1.16) |
| Adds salt to food frequently | p1478 | Salt added to food | Ordinal categorical | 1478 | Lifestyle | Coding: 1=Never/rarely; 2=Sometimes; 3=Usually; 4=Always | 501,282 (0.22) |
| Tea intake | p1488 | Tea intake | Continuous | 1488 | Lifestyle | Standardized (mean = 0, SD = 1) | 500,215 (0.44) |
| Coffee intake | p1498 | Coffee intake | Continuous | 1498 | Lifestyle | Standardized (mean = 0, SD = 1) | 500,141 (0.45) |
| Instant coffee | p1508_1 | Coffee type | Categorical | 1508 | Lifestyle | Coding: 0=Decaffeinated coffee (any type); 1=Instant coffee | 384,339 (23.5) |
| Ground coffee | p1508_2 | Coffee type | Categorical | 1508 | Lifestyle | Coding: 0=Decaffeinated coffee (any type); 1=Ground coffee (include espresso, filter etc) | 384,339 (23.5) |
| Other coffee types | p1508_3 | Coffee type | Categorical | 1508 | Lifestyle | Coding: 0=Decaffeinated coffee (any type); 1=Other type of coffee | 384,339 (23.5) |
| Hot drink temperature | p1518 | Hot drink temperature | Ordinal categorical | 1518 | Lifestyle | Coding: 1=Do not drink hot drinks; 2=Warm; 3=Hot; 4=Very hot | 501,180 (0.24) |
| Water intake | p1528 | Water intake | Continuous | 1528 | Lifestyle | Standardized (mean = 0, SD = 1) | 497,627 (0.95) |
| Major dietary changes | p1538 | Major dietary changes in the last 5 years | Categorical | 1538 | Lifestyle | Dummy coding: 0=No; 1=Yes, because of illness/Yes, because of other reasons | 500,044 (0.47) |
| Diet varies frequently | p1548 | Variation in diet | Ordinal categorical | 1548 | Lifestyle | Coding: 1=Never/rarely; 2=Sometimes; 3=Often | 495,218 (1.43) |
| Right-handed | p1707_1 | Handedness (chirality/laterality) | Categorical | 1707 | Lifestyle | Dummy coding: 0=Use both right and left hands equally; 1=Right-handed | 501,227 (0.24) |
| Left-handed | p1707_2 | Handedness (chirality/laterality) | Categorical | 1707 | Lifestyle | Dummy coding: 0=Use both right and left hands equally; 1=Left-handed | 501,227 (0.24) |
| Previous smoker | p20116_1 | Smoking status | Categorical | 20116 | Lifestyle | Dummy coding: 0=Never; 1=Previous | 499,459 (0.59) |
| Current smoker | p20116_2 | Smoking status | Categorical | 20116 | Lifestyle | Dummy coding: 0=Never; 1=Current | 499,459 (0.59) |
| Ever smoked | p20160 | Ever smoked | Categorical | 20160 | Lifestyle | Coding: 0=No; 1=Yes | 499,523 (0.57) |
| First sexual intercourse (< age 18) | p2139 | Age first had sexual intercourse | Categorical | 2139 | Lifestyle | Dummy coding: 0=age>=18; 1= age<18 | 435,333 (13.35) |
| Lifetime number of sexual partners | p2149 | Lifetime number of sexual partners | Continuous | 2149 | Lifestyle | Standardized (mean = 0, SD = 1) | 405,024 (19.38) |
| Had same-sex intercourse | p2159 | Ever had same-sex intercourse | Categorical | 2159 | Lifestyle | Coding: 0=No; 1=Yes | 448,726 (10.69) |
| IPAQ activity level | p22032 | IPAQ activity group | Ordinal categorical | 22032 | Lifestyle | Coding: 1=low; 2=moderate; 3=high | 402,301 (19.93) |
| Summed days activity | p22033 | Summed days activity | Continuous | 22033 | Lifestyle | Standardized (mean = 0, SD = 1) | 413,957 (17.61) |
| Summed minutes activity | p22034 | Summed minutes activity | Continuous | 22034 | Lifestyle | Standardized (mean = 0, SD = 1) | 402,301 (19.93) |
| At or above moderate/vigorous recommendation | p22035 | At or above moderate/vigorous recommendation | Categorical | 22035 | Lifestyle | Coding: 0=No; 1=Yes | 402,301 (19.93) |
| At or above moderate/vigorous/walking recommendation | p22036 | At or above moderate/vigorous/walking recommendation | Categorical | 22036 | Lifestyle | Coding: 0=No; 1=Yes | 402,189 (19.95) |
| MET minutes/week of walking | p22037 | MET minutes per week for walking | Continuous | 22037 | Lifestyle | Standardized (mean = 0, SD = 1) | 402,301 (19.93) |
| MET minutes/week of moderate activity | p22038 | MET minutes per week for moderate activity | Continuous | 22038 | Lifestyle | Standardized (mean = 0, SD = 1) | 402,301 (19.93) |
| MET minutes/week of vigorous activity | p22039 | MET minutes per week for vigorous activity | Continuous | 22039 | Lifestyle | Standardized (mean = 0, SD = 1) | 402,301 (19.93) |
| MET minutes/week of all activity | p22040 | Summed MET minutes per week for all activity | Continuous | 22040 | Lifestyle | Standardized (mean = 0, SD = 1) | 402,301 (19.93) |
| Plays computer games frequently | p2237 | Plays computer games | Ordinal categorical | 2237 | Lifestyle | Coding: 0=Never/rarely; 1=Sometimes; 2=Often | 500,700 (0.34) |
| Never eat eggs | p6144_1 | Never eat eggs or foods containing eggs | Categorical | 6144 | Lifestyle | Dummy coded from array variables. Dummy coding: 0=No; 1=Yes | 495,971 (1.28) |
| Never eat dairy products | p6144_2 | Never eat dairy products | Categorical | 6144 | Lifestyle | Dummy coded from array variables. Dummy coding: 0=No; 1=Yes | 495,971 (1.28) |
| Never eat wheat products | p6144_3 | Never eat wheat products | Categorical | 6144 | Lifestyle | Dummy coded from array variables. Dummy coding: 0=No; 1=Yes | 495,971 (1.28) |
| Never eat sugar foods | p6144_4 | Never eat sugar or foods/drinks containing sugar | Categorical | 6144 | Lifestyle | Dummy coded from array variables. Dummy coding: 0=No; 1=Yes | 495,971 (1.28) |
| Attends a sports club or gym frequently | p6160_1 | Attending a sports club or gym once a week or more often | Categorical | 6160 | Lifestyle | Dummy coded from array variables. Dummy coding: 0=No; 1=Yes | 499,574 (0.56) |
| Attends a pub or social club frequently | p6160_2 | Attending a pub or social club once a week or more often | Categorical | 6160 | Lifestyle | Dummy coded from array variables. Dummy coding: 0=No; 1=Yes | 499,574 (0.56) |
| Attends a religious group frequently | p6160_3 | Attending a religious group once a week or more often | Categorical | 6160 | Lifestyle | Dummy coded from array variables. Dummy coding: 0=No; 1=Yes | 499,574 (0.56) |
| Attends a adult education class frequently | p6160_4 | Attending a adult education class once a week or more often | Categorical | 6160 | Lifestyle | Dummy coded from array variables. Dummy coding: 0=No; 1=Yes | 499,574 (0.56) |
| Attends other group activities frequently | p6160_5 | Attending a other group activity once a week or more often | Categorical | 6160 | Lifestyle | Dummy coded from array variables. Dummy coding: 0=No; 1=Yes | 499,574 (0.56) |
| Uses car/motor vehicle as transport | p6162_1 | Car/motor vehicle | Categorical | 6162 | Lifestyle | Dummy coded from array variables. Dummy coding: 0=No; 1=Yes | 495,545 (1.37) |
| Walks as transport | p6162_2 | Walk | Categorical | 6162 | Lifestyle | Dummy coded from array variables. Dummy coding: 0=No; 1=Yes | 495,545 (1.37) |
| Uses public transport | p6162_3 | Public transport | Categorical | 6162 | Lifestyle | Dummy coded from array variables. Dummy coding: 0=No; 1=Yes | 495,545 (1.37) |
| Uses cycle as transport | p6162_4 | Cycle | Categorical | 6162 | Lifestyle | Dummy coded from array variables. Dummy coding: 0=No; 1=Yes | 495,545 (1.37) |
| Walks for pleasure | p6164_1 | Walking for pleasure (not as a means of transport) | Categorical | 6164 | Lifestyle | Dummy coded from array variables. Dummy coding: 0=No; 1=Yes | 495,257 (1.42) |
| Engages in other exercises | p6164_2 | Other exercises (eg: swimming, cycling, keep fit, bowling) | Categorical | 6164 | Lifestyle | Dummy coded from array variables. Dummy coding: 0=No; 1=Yes | 495,257 (1.42) |
| Engages in strenuous sports | p6164_3 | Strenuous sports | Categorical | 6164 | Lifestyle | Dummy coded from array variables. Dummy coding: 0=No; 1=Yes | 495,257 (1.42) |
| Light DIY | p6164_4 | Light DIY (eg: pruning, watering the lawn) | Categorical | 6164 | Lifestyle | Dummy coded from array variables. Dummy coding: 0=No; 1=Yes | 495,257 (1.42) |
| Heavy DIY | p6164_5 | Heavy DIY (eg: weeding, lawn mowing, carpentry, digging) | Categorical | 6164 | Lifestyle | Dummy coded from array variables. Dummy coding: 0=No; 1=Yes | 495,257 (1.42) |
| Days/week walked 10+ minutes | p864 | Number of days/week walked 10+ minutes | Continuous | 864 | Lifestyle | Standardized (mean = 0, SD = 1) | 493,871 (1.70) |
| Duration of walks | p874 | Duration of walks | Continuous | 874 | Lifestyle | Standardized (mean = 0, SD = 1) | 425,495 (15.31) |
| Days/week moderate physical activity 10+ minutes | p884 | Number of days/week of moderate physical activity 10+ minutes | Continuous | 884 | Lifestyle | Standardized (mean = 0, SD = 1) | 475,143 (5.43) |
| Duration of moderate activity | p894 | Duration of moderate activity | Continuous | 894 | Lifestyle | Standardized (mean = 0, SD = 1) | 368,340 (26.69) |
| Improper consumption of alcohol | p900_1 | Non-moderate drinking | Categorical | 1568, 1578, 1588, 1598, 1608, 5364, 4407, 4418, 4429, 4440, 4451, 4462, 1558 | Lifestyle | Coding: 0=Proper consumption; 1=Improper consumption of alcohol. For further details on the derivation and coding of this variable, see the *Notes*. | 427,638 (14.88) |
| Improper sleep duration <7/>9 hours | p900_2 | Improper sleep duration | Categorical | 1160 | Lifestyle | Coding: 0=No; 1=Yes | 498,193 (0.84) |
| Vegetable eaten above recommendation | p900_3 | Vegetable eaten above recommendation | Categorical | 1289, 1299 | Lifestyle | Coding: 0=No; 1=Yes | 497,921 (0.89) |
| Fruit eaten above recommendation | p900_4 | Fruit eaten above recommendation | Categorical | 1309, 1319 | Lifestyle | Coding: 0=No; 1=Yes | 500,268 (0.43) |
| Fish eaten above recommendation | p900_5 | Fish eaten above recommendation | Categorical | 1329, 1339 | Lifestyle | Coding: 0=No; 1=Yes | 498,653 (0.75) |
| Whole bread/refined cereals intake reduced | p900_6 | Whole bread/refined cereals intake reduced | Categorical | 1448, 1468, 1438, 1458 | Lifestyle | Coding: 0=No; 1=Yes | 494,891 (1.50) |
| Wholegrain eaten above recommendation | p900_7 | Wholegrain eaten above recommendation | Categorical | 1448, 1468, 1438, 1458 | Lifestyle | Coding: 0=No; 1=Yes | 494,891 (1.50) |
| Red meat intake reduced | p900_8 | Red meat intake reduced | Categorical | 1369, 1379, 1389 | Lifestyle | Coding: 0=No; 1=Yes | 500,554 (0.37) |
| Processed meat intake reduced | p900_9 | Processed meat intake reduced | Categorical | 1349 | Lifestyle | Coding: 0=No; 1=Yes | 500,178 (0.44) |
| Days/week of vigorous physical activity | p904 | Number of days/week of vigorous physical activity 10+ minutes | Continuous | 904 | Lifestyle | Standardized (mean = 0, SD = 1) | 474,834 (5.49) |
| Usually walks quickly | p924 | Usual walking pace | Ordinal categorical | 924 | Lifestyle | Coding: 1=Slow pace; 2=Steady average pace; 3=Brisk pace | 497,731 (0.93) |
| Climbes stair frequently | p943 | Frequency of stair climbing in last 4 weeks | Ordinal categorical | 943 | Lifestyle | Coding: 0=None; 1=1-5 times a day; 2=6-10 times a day; 3=11-15 times a day; 4=16-20 times a day; 5=More than 20 times a day | 492,809 (1.91) |
| Duration of walking for pleasure | p981 | Duration walking for pleasure | Ordinal categorical | 981 | Lifestyle | Coding: 1=Less than 15 minutes; 2=Between 15 and 30 minutes; 3=Between 30 minutes and 1 hour; 4=Between 1 and 1.5 hours; 5=Between 1.5 and 2 hours; 6=Between 2 and 3 hours; 7=Over 3 hours | 349,061 (30.52) |
| Greenspace percentage (1000 m buffer) | p24500 | Greenspace percentage, buffer 1000m | Continuous | 24500 | Ecosystems | Standardized (mean = 0, SD = 1) | 440,783 (12.27) |
| Domestic garden percentage (1000 m buffer) | p24501 | Domestic garden percentage, buffer 1000m | Continuous | 24501 | Ecosystems | Standardized (mean = 0, SD = 1) | 440,783 (12.27) |
| Water percentage (1000 m buffer) | p24502 | Water percentage, buffer 1000m | Continuous | 24502 | Ecosystems | Standardized (mean = 0, SD = 1) | 440,783 (12.27) |
| Greenspace percentage (300 m buffer) | p24503 | Greenspace percentage, buffer 300m | Continuous | 24503 | Ecosystems | Standardized (mean = 0, SD = 1) | 440,783 (12.27) |
| Domestic garden percentage (300 m buffer) | p24504 | Domestic garden percentage, buffer 300m | Continuous | 24504 | Ecosystems | Standardized (mean = 0, SD = 1) | 440,783 (12.27) |
| Water percentage (300 m buffer) | p24505 | Water percentage, buffer 300m | Continuous | 24505 | Ecosystems | Standardized (mean = 0, SD = 1) | 440,783 (12.27) |
| Natural environment percentage (1000m buffer) | p24506 | Natural environment percentage, buffer 1000m | Continuous | 24506 | Ecosystems | Standardized (mean = 0, SD = 1) | 497,446 (0.99) |
| Natural environment percentage (300 m buffer) | p24507 | Natural environment percentage, buffer 300m | Continuous | 24507 | Ecosystems | Standardized (mean = 0, SD = 1) | 497,446 (0.99) |
| Distance to coast | p24508 | Distance (Euclidean) to coast | Continuous | 24508 | Ecosystems | Standardized (mean = 0, SD = 1) | 497,446 (0.99) |
| Had smoking/smokers in household | p1259 | Smoking/smokers in household | Categorical | 1259 | Chemical | Coding: 0=No; 1=Yes, one household member smokes/Yes, more than one household member smokes | 457,947 (8.85) |
| Exposure to tobacco smoke at home | p1269 | Exposure to tobacco smoke at home | Categorical | 1269 | Chemical | Turn data into binary classification. Dummy coding: 0=no exposure; 1=exposure to tobacco smoke at home | 451,162 (10.2) |
| Exposure to tobacco smoke outside home | p1279 | Exposure to tobacco smoke outside home | Categorical | 1279 | Chemical | Turn data into binary classification. Dummy coding: 0=no exposure; 1=exposure to tobacco smoke outside home | 421,581 (16.09) |
| Number of treatments/medications taken | p137 | Number of treatments/medications taken | Continuous | 137 | Chemical | Standardized (mean = 0, SD = 1) | 501,546 (0.17) |
| Breastfed as a baby | p1677 | Breastfed as a baby | Categorical | 1677 | Chemical | Coding: 0=No; 1=Yes | 383,634 (23.64) |
| Maternal smoking around birth | p1787 | Maternal smoking around birth | Categorical | 1787 | Chemical | Coding: 0=No; 1=Yes | 432,785 (13.86) |
| Nitrogen dioxide/oxides air pollution | p2400_1 | Nitrogen dioxide/oxides air pollution | Continuous | 24003, 24004, 24016, 24017, 24018. | Chemical | Mean values of fields 24003, 24004, 24016, 24017, and 24018. Standardized (mean = 0, SD = 1) | 495,031 (1.47) |
| PM10 pollution | p2400_2 | Particulate matter air pollution (pm10) | Continuous | 24005, 24019 | Chemical | Mean values of fields 24005 and 24019. Standardized (mean = 0, SD = 1) | 495,020 (1.47) |
| PM2.5 pollution | p24006 | Particulate matter air pollution (pm2.5); 2010 | Continuous | 24006 | Chemical | Standardized (mean = 0, SD = 1) | 461,110 (8.22) |
| PM2.5 absorbance pollution | p24007 | Particulate matter air pollution (pm2.5) absorbance; 2010 | Continuous | 24007 | Chemical | Standardized (mean = 0, SD = 1) | 461,110 (8.22) |
| PM2.5-10 pollution | p24008 | Particulate matter air pollution 2.5-10um; 2010 | Continuous | 24008 | Chemical | Standardized (mean = 0, SD = 1) | 461,110 (8.22) |
| Takes other prescription medications | p2492 | Taking other prescription medications | Categorical | 2492 | Chemical | Coding: 0=No; 1=Yes - you will be asked about this later by an interviewer | 499,340 (0.61) |
| Uses gas hob or cooker | p6139_1 | A gas hob or gas cooker | Categorical | 6139 | Chemical | Dummy coded from array variables. Dummy coding: 0=No; 1=Yes | 496,773 (1.12) |
| Uses gas fire in winter | p6139_2 | A gas fire that you use regularly in winter time | Categorical | 6139 | Chemical | Dummy coded from array variables. Dummy coding: 0=No; 1=Yes | 496,773 (1.12) |
| Uses open solid fuel fire in winter | p6139_3 | An open solid fuel fire that you use regularly in winter time | Categorical | 6139 | Chemical | Dummy coded from array variables. Dummy coding: 0=No; 1=Yes | 496,773 (1.12) |
| Usually takes Aspirin | p6154_1 | Usually take Aspirin | Categorical | 6154 | Chemical | Dummy coded from array variables. Dummy coding: 0=No; 1=Yes | 496,728 (1.13) |
| Usually takes Ibuprofen | p6154_2 | Usually take Ibuprofen (e.g. Nurofen) | Categorical | 6154 | Chemical | Dummy coded from array variables. Dummy coding: 0=No; 1=Yes | 496,728 (1.13) |
| Usually takes Paracetamol | p6154_3 | Usually take Paracetamol | Categorical | 6154 | Chemical | Dummy coded from array variables. Dummy coding: 0=No; 1=Yes | 496,728 (1.13) |
| Usually takes Ranitidine | p6154_4 | Usually take Ranitidine (e.g. Zantac) | Categorical | 6154 | Chemical | Dummy coded from array variables. Dummy coding: 0=No; 1=Yes | 496,728 (1.13) |
| Usually takes Omeprazole | p6154_5 | Usually take Omeprazole (e.g. Zanprol) | Categorical | 6154 | Chemical | Dummy coded from array variables. Dummy coding: 0=No; 1=Yes | 496,728 (1.13) |
| Usually takes Laxatives | p6154_6 | Usually take Laxatives (e.g. Dulcolax, Senokot) | Categorical | 6154 | Chemical | Dummy coded from array variables. Dummy coding: 0=No; 1=Yes | 496,728 (1.13) |
| Vitamin A supplements | p6155_1 | Vitamin A supplements | Categorical | 6155 | Chemical | Dummy coded from array variables. Dummy coding: 0=No; 1=Yes | 495,072 (1.46) |
| Vitamin B supplements | p6155_2 | Vitamin B supplements | Categorical | 6155 | Chemical | Dummy coded from array variables. Dummy coding: 0=No; 1=Yes | 495,072 (1.46) |
| Vitamin C supplements | p6155_3 | Vitamin C supplements | Categorical | 6155 | Chemical | Dummy coded from array variables. Dummy coding: 0=No; 1=Yes | 495,072 (1.46) |
| Vitamin D supplements | p6155_4 | Vitamin D supplements | Categorical | 6155 | Chemical | Dummy coded from array variables. Dummy coding: 0=No; 1=Yes | 495,072 (1.46) |
| Vitamin E supplements | p6155_5 | Vitamin E supplements | Categorical | 6155 | Chemical | Dummy coded from array variables. Dummy coding: 0=No; 1=Yes | 495,072 (1.46) |
| Folic acid or Folate (Vit B9) supplements | p6155_6 | Folic acid or Folate (Vit B9) supplements | Categorical | 6155 | Chemical | Dummy coded from array variables. Dummy coding: 0=No; 1=Yes | 495,072 (1.46) |
| Multivitamins +/- minerals supplements | p6155_7 | Multivitamins +/- minerals supplements | Categorical | 6155 | Chemical | Dummy coded from array variables. Dummy coding: 0=No; 1=Yes | 495,072 (1.46) |
| Fish oil (including cod liver oil) supplements | p6179_1 | Fish oil (including cod liver oil) supplements | Categorical | 6179 | Chemical | Dummy coded from array variables. Dummy coding: 0=No; 1=Yes | 496,213 (1.23) |
| Glucosamine supplements | p6179_2 | Glucosamine supplements | Categorical | 6179 | Chemical | Dummy coded from array variables. Dummy coding: 0=No; 1=Yes | 496,213 (1.23) |
| Calcium supplements | p6179_3 | Calcium supplements | Categorical | 6179 | Chemical | Dummy coded from array variables. Dummy coding: 0=No; 1=Yes | 496,213 (1.23) |
| Zinc supplements | p6179_4 | Zinc supplements | Categorical | 6179 | Chemical | Dummy coded from array variables. Dummy coding: 0=No; 1=Yes | 496,213 (1.23) |
| Iron supplements | p6179_5 | Iron supplements | Categorical | 6179 | Chemical | Dummy coded from array variables. Dummy coding: 0=No; 1=Yes | 496,213 (1.23) |
| Selenium supplements | p6179_6 | Selenium supplements | Categorical | 6179 | Chemical | Dummy coded from array variables. Dummy coding: 0=No; 1=Yes | 496,213 (1.23) |

**Notes:** Adherence to a healthy diet is characterised by increased consumption of fruits, nuts, vegetables, whole grains, fish, and dairy products, and reduced consumption of refined grains, processed meats, unprocessed red meats, and sugar-sweetened beverages. We determined the recommendation intake level for fruit, fish, wholegrain, and et al. based on previous research.^1,2^ Moderate alcohol intake was defined as 5–15 g/day for women and 5–30 g/day for men. Alcohol intake for participants who self-reported as non-drinkers was assumed to be 0 g/day.^1,2^

**References**

1. Bountziouka V, Musicha C, Allara E, Kaptoge S, Wang Q, Angelantonio ED, Butterworth AS, Thompson JR, Danesh JN, Wood AM, Nelson CP, Codd V, Samani NJ. Modifiable traits, healthy behaviours, and leukocyte telomere length: a population-based study in UK Biobank. *Lancet Healthy Longev.* 2022 May;3(5):e321-e331. doi: 10.1016/S2666-7568(22)00072-1.

2. Zhang, Y., Chen, SD., Deng, YT. *et al.* Identifying modifiable factors and their joint effect on dementia risk in the UK Biobank. *Nat Hum Behav* **7**, 1185–1195 (2023). <https://doi.org/10.1038/s41562-023-01585-x>

**Supplementary Table 3. Baseline characteristics of the studied population**

|  |  | **Overall** | **Healthy individuals** | **Incident ADRD** | **Incident AD** |
| --- | --- | --- | --- | --- | --- |
|  |  | (N=499,992) | (N=491,111) | (N=8,881) | (N=4,000) |
| **Age (median [IQR])** |  | 58.0 [50.0, 63.0] | 58.0 [50.0, 63.0] | 65.0 [62.0, 68.0] | 66.0 [63.0, 68.0] |
| **Sex (N, %)** | Female | 272,058 (54.4) | 267,852 (54.5) | 4,206 (47.4) | 2,086 (52.1) |
|  | Male | 227,934 (45.6) | 223,259 (45.5) | 4,675 (52.6) | 1,914 (47.9) |
| **Genetic risk (N, %)** | Low | 62,360 (12.9) | 61,746 (13.0) | 614 (7.2) | 202 (5.3) |
|  | Intermediate | 284,940 (58.8) | 133,086 (27.9) | 3,393 (39.9) | 1,256 (32.7) |
|  | High | 137,585 (28.4) | 133,454 (27.9) | 4,499 (52.9) | 2,381 (62.0) |
| **Overall health (N, %)** | Excellent | 81,499 (16.4) | 80,616 (16.5) | 883 (10.1) | 470 (11.8) |
|  | Good | 287,807 (58.0) | 283,305 (58.1) | 4,502 (51.3) | 2,173 (54.8) |
|  | Fair | 104,710 (21.1) | 102,192 (21.0) | 2,518 (28.7) | 1,062 (26.8) |
|  | Poor | 22,533 (4.5) | 21,664 (4.4) | 869 (9.9) | 262 (6.6) |
| **Ethnicity (N, %)** | White | 470,394 (94.6) | 461,928 (94.6) | 8,466 (96.0) | 3,839 (96.6) |
|  | Mixed | 2,933 (0.6) | 2,904 (0.6) | 29 (0.3) | 11 (0.3) |
|  | Asian | 9,830 (2.0) | 9,700 (2.0) | 130 (1.5) | 52 (1.3) |
|  | Black | 8,002 (1.6) | 7,874 (1.6) | 128 (1.5) | 50 (1.3) |
|  | Other | 6,087 (1.2) | 6,025 (1.2) | 62 (0.7) | 24 (0.6) |
| **Follow up years (median [IQR])** |  | 15.3 [14.5, 16.0] | 15.3 [14.6, 16.1] | 9.5 [6.5, 12.1] | 10.1 [7.0, 12.4] |

**Notes:** Genetic risks were evaluated using the *APOE* genotype, with the presence of ε4 indicating a high genetic risk, ε3ε3 an intermediate risk and others a low risk.

**Supplementary Table 4. Baseline characteristics of the studied population in derivation set**

|  |  | **Overall** | **Healthy individuals** | **Incident ADRD** | **Incident AD** |
| --- | --- | --- | --- | --- | --- |
|  |  | (N= 333,328) | (N= 327,402) | (N= 5,926) | (N= 2,674) |
| **Age (median [IQR])** |  | 58.0 [50.0, 63.0] | 58.0 [50.0, 63.0] | 65.0 [62.0, 68.0] | 66.0 [63.0, 68.0] |
| **Sex (N, %)** | Female | 181,413 (54.4) | 178,621 (54.6) | 2,792 (47.1) | 1,381 (51.6) |
|  | Male | 151,915 (45.6) | 148,781 (45.4) | 3,134 (52.9) | 1,293 (48.4) |
| **Genetic risk (N, %)** | Low | 41,554 (12.9) | 41,146 (13.0) | 408 (7.2) | 120 (4.7) |
|  | Intermediate | 189,888 (58.7) | 187,633 (59.1) | 2,255 (39.7) | 843 (32.8) |
|  | High | 91,842 (28.4) | 88,832 (28.0) | 3,010 (53.1) | 1,606 (62.5) |
| **Overall health (N, %)** | Excellent | 54,261 (16.4) | 53,662 (16.5) | 599 (10.2) | 314 (11.8) |
|  | Good | 191,758 (57.9) | 188,779 (58.0) | 2,979 (51.0) | 1,441 (54.4) |
|  | Fair | 69,961 (21.1) | 68,289 (21.0) | 1,672 (28.6) | 714 (26.9) |
|  | Poor | 15,125 (4.6) | 14,529 (4.5) | 596 (10.2) | 181 (6.8) |
| **Ethnicity (N, %)** | White | 313,651 (94.6) | 308,006 (94.6) | 5,645 (96.0) | 2,568 (96.6) |
|  | Mixed | 1,979 (0.6) | 1,957 (0.6) | 22 (0.4) | 8 (0.3) |
|  | Asian | 6,554 (2.0) | 6,472 (2.0) | 82 (1.4) | 29 (1.1) |
|  | Black | 5,335 (1.6) | 5,244 (1.6) | 91 (1.5) | 40 (1.5) |
|  | Other | 4,025 (1.2) | 3,984 (1.2) | 41 (0.7) | 13 (0.5) |
| **Follow up years (median [IQR])** |  | 15.3 [14.6, 16.0] | 15.3 [14.6, 16.1] | 9.4 [6.3, 12.1] | 10.1 [7.1, 12.4] |

**Notes:** Genetic risks were evaluated using the *APOE* genotype, with the presence of ε4 indicating a high genetic risk, ε3ε3 an intermediate risk and others a low risk.

**Supplementary Table 5. Baseline characteristics of the studied population in replication set**

|  | **Feature** | **Overall** | **Healthy controls** | **Incident ADRD** | **Incident AD** |
| --- | --- | --- | --- | --- | --- |
|  |  | (N= 166,664) | (N= 163,709) | (N= 2,955) | (N= 1,326) |
| **Age (median [IQR])** |  | 58.0 [50.0, 63.0] | 58.0 [50.0, 63.0] | 65.0 [62.0, 68.0] | 65.0 [62.0, 68.0] |
| **Sex (N, %)** | Female | 90,645 (54.4) | 89,231 (54.5) | 1,414 (47.9) | 705 (53.2) |
|  | Male | 76,019 (45.6) | 74,478 (45.5) | 1,541 (52.1) | 621 (46.8) |
| **Genetic risk (N, %)** | Low | 20,806 (12.9) | 20,600 (13.0) | 206 (7.3) | 82 (6.5) |
|  | Intermediate | 95,052 (58.8) | 93,917 (59.2) | 1,135 (40.1) | 413 (32.5) |
|  | High | 45,743 (28.3) | 44,254 (27.9) | 1,489 (52.6) | 775 (61.0) |
| **Overall health (N, %)** | Excellent | 27,238 (16.5) | 26,954 (16.6) | 284 (9.7) | 156 (11.8) |
|  | Good | 96,049 (58.1) | 94,526 (58.2) | 1,523 (52.1) | 732 (55.6) |
|  | Fair | 34,749 (21.0) | 33,903 (20.9) | 846 (28.9) | 348 (26.4) |
|  | Poor | 7,408 (4.5) | 7,135 (4.4) | 273 (9.3) | 81 (6.2) |
| **Ethnicity (N, %)** | White | 156,743 (94.6) | 153,922 (94.6) | 2,821 (96.1) | 1,271 (96.4) |
|  | Mixed | 954 (0.6) | 947 (0.6) | 7 (0.2) | 3 (0.2) |
|  | Asian | 3,276 (2.0) | 3,228 (2.0) | 48 (1.6) | 23 (1.7) |
|  | Black | 2,667 (1.6) | 2,630 (1.6) | 37 (1.3) | 10 (0.8) |
|  | Other | 2,062 (1.2) | 2,041 (1.3) | 21 (0.7) | 11 (0.8) |
| **Follow up years (median [IQR])** |  | 15.3 [14.6, 16.1] | 15.3 [14.6, 16.1] | 9.2 [6.1, 12.1] | 10.2 [6.8, 12.5] |

**Notes:** Genetic risks were evaluated using the *APOE* genotype, with the presence of ε4 indicating a high genetic risk, ε3ε3 an intermediate risk and others a low risk.

**Supplementary Table 6. Baseline characteristics of the studied population by subgroups**

|  |  | **Female** | **Male** | **Age<65** | **Age>=65** | **Low genetic risk** | **Intermediate genetic risk** | **High genetic risk** | **White**  **ethnicity** | **All other ethnicities** |
| --- | --- | --- | --- | --- | --- | --- | --- | --- | --- | --- |
|  |  | (N=272,058) | (N=227,934) | (N=404,810) | (N=95,182) | (N=62,360) | (N=284,940) | (N=137,585) | (N=470,394) | (N=26,852) |
| **Age (median [IQR])** |  | 57.0 [50.0, 63.0] | 58.0 [50.0, 64.0] | 55.0 [48.0, 60.0] | 67.0 [66.0, 68.0] | 58.0 [50.0, 63.0] | 58.0 [50.0, 63.0] | 58.0 [50.0, 63.0] | 58.0 [50.0, 63.0] | 52.0 [46.0, 59.0] |
| **Sex (N, %)** | Female | 272,058 (100.0%) | 0 (0.0%) | 223,894 (55.3%) | 48,164 (50.6%) | 33,822 (54.2%) | 154,499 (54.2%) | 74,619 (54.2%) | 256,219 (54.5%) | 14,588 (54.3%) |
|  | Male | 0 (0.0%) | 227,934 (100.0%) | 180,916 (44.7%) | 47,018 (49.4%) | 28,538 (45.8%) | 130,441 (45.8%) | 62,966 (45.8%) | 214,175 (45.5%) | 12,264 (45.7%) |
| **Genetic risk (N, %)** | Low | 33,822 (12.9%) | 28,538 (12.9%) | 50,328 (12.8%) | 12,032 (13.0%) | 62,360 (100.0%) | 0 (0.0%) | 0 (0.0%) | 59,081 (12.9%) | 2,998 (11.7%) |
|  | Intermediate | 154,499 (58.8%) | 130,441 (58.8%) | 230,387 (58.7%) | 54,553 (59.0%) | 0 (0.0%) | 284,940 (100.0%) | 0 (0.0%) | 267,886 (58.6%) | 15,665 (61.2%) |
|  | High | 74,619 (28.4%) | 62,966 (28.4%) | 111,767 (28.5%) | 25,818 (27.9%) | 0 (0.0%) | 0 (0.0%) | 137,585 (100.0%) | 130,039 (28.5%) | 6,924 (27.1%) |
| **Overall health (N, %)** | Excellent | 46,125 (17.1%) | 35,374 (15.6%) | 68,215 (17.0%) | 13,284 (14.0%) | 10,144 (16.4%) | 46,788 (16.5%) | 22,485 (16.4%) | 78,507 (16.8%) | 2,773 (10.5%) |
|  | Good | 160,888 (59.5%) | 126,919 (56.1%) | 231,274 (57.5%) | 56,533 (59.8%) | 36,248 (58.4%) | 163,784 (57.8%) | 79,698 (58.3%) | 273,247 (58.3%) | 13,641 (51.8%) |
|  | Fair | 52,593 (19.5%) | 52,117 (23.0%) | 83,720 (20.8%) | 20,990 (22.2%) | 12,879 (20.8%) | 59,784 (21.1%) | 28,580 (20.9%) | 96,515 (20.6%) | 7,789 (29.6%) |
|  | Poor | 10,661 (3.9%) | 11,872 (5.2%) | 18,772 (4.7%) | 3,761 (4.0%) | 2,747 (4.4%) | 12,818 (4.5%) | 6,005 (4.4%) | 20,270 (4.3%) | 2,126 (8.1%) |
| **Ethnicity (N, %)** | White | 256,219 (94.6%) | 214,175 (94.6%) | 378,613 (94.0%) | 91,781 (97.0%) | 59,081 (95.2%) | 267,886 (94.5%) | 130,039 (94.9%) | 470,394 (100.0%) | 0 (0.0%) |
|  | Mixed | 1,837 (0.7%) | 1,096 (0.5%) | 2,673 (0.7%) | 260 (0.3%) | 324 (0.5%) | 1,705 (0.6%) | 790 (0.6%) | 0 (0.0%) | 2,933 (10.9%) |
|  | Asian | 4,562 (1.7%) | 5,268 (2.3%) | 8,580 (2.1%) | 1,250 (1.3%) | 766 (1.2%) | 6,881 (2.4%) | 1,720 (1.3%) | 0 (0.0%) | 9,830 (36.6%) |
|  | Black | 4,624 (1.7%) | 3,378 (1.5%) | 7,193 (1.8%) | 809 (0.9%) | 1,206 (1.9%) | 3,308 (1.2%) | 3,073 (2.2%) | 0 (0.0%) | 8,002 (29.8%) |
|  | Other | 3,565 (1.3%) | 2,522 (1.1%) | 5,548 (1.4%) | 539 (0.6%) | 702 (1.1%) | 3,771 (1.3%) | 1,341 (1.0%) | 0 (0.0%) | 6,087 (22.7%) |
| **Follow up years (median [IQR])** |  | 15.3 [14.6, 16.1] | 15.3 [14.4, 16.0] | 15.4 [14.6, 16.1] | 15.0 [14.1, 15.9] | 15.3 [14.6, 16.1] | 15.3 [14.6, 16.0] | 15.3 [14.5, 16.0] | 15.3 [14.6, 16.1] | 14.8 [14.3, 15.7] |

**Notes:** Genetic risks were evaluated using the *APOE* genotype, with the presence of ε4 indicating a high genetic risk, ε3ε3 an intermediate risk and others a low risk.

**Supplementary Table 7. Associations between 193 exposures and ADRD in ExWAS**

| **Variable code** | **HR** | **Lower confidence interval of HR** | **Upper confidence interval of HR** | **P** | **P**  **Bonferroni**  **adjust** | **Category** | **Variable** |
| --- | --- | --- | --- | --- | --- | --- | --- |
| p6146 | 1.9395 | 1.7918 | 2.0993 | 2.06E-60 | 3.98E-58 | Social | Disability |
| p680 | 1.6449 | 1.5215 | 1.7782 | 6.26E-36 | 1.21E-33 | Social | Rent accommodation versus own |
| p1100 | 0.7584 | 0.7258 | 0.7925 | 5.85E-35 | 1.13E-32 | Lifestyle | Frequent motorway speeding |
| p1080 | 0.8587 | 0.8381 | 0.8799 | 1.42E-34 | 2.73E-32 | Lifestyle | Time spent using computer |
| p728 | 0.8039 | 0.7756 | 0.8334 | 1.19E-32 | 2.29E-30 | Social | Number of vehicles in household |
| p738 | 0.8576 | 0.8352 | 0.8807 | 7.54E-30 | 1.46E-27 | Social | Total household income |
| p6154_6 | 1.7367 | 1.5584 | 1.9354 | 1.76E-23 | 3.39E-21 | Chemical | Usually takes Laxatives |
| p2492 | 1.3536 | 1.2751 | 1.4369 | 2.79E-23 | 5.39E-21 | Chemical | Takes other prescription medications |
| p6144_1 | 1.76 | 1.5731 | 1.969 | 5.45E-23 | 1.05E-20 | Lifestyle | Never eat eggs |
| p1090 | 0.8806 | 0.8586 | 0.9032 | 6.51E-23 | 1.26E-20 | Lifestyle | Time spent driving |
| p6162_1 | 0.7507 | 0.7077 | 0.7963 | 1.61E-21 | 3.11E-19 | Lifestyle | Uses car/motor vehicle as transport |
| p6142 | 1.7268 | 1.5431 | 1.9325 | 1.81E-21 | 3.5E-19 | Social | Unemployed |
| p137 | 1.1925 | 1.1496 | 1.237 | 4.47E-21 | 8.63E-19 | Chemical | Number of medications taken |
| p6164_2 | 0.7832 | 0.7403 | 0.8285 | 1.77E-17 | 3.42E-15 | Lifestyle | Engages in other exercises |
| p6141 | 0.7797 | 0.7361 | 0.8258 | 2.05E-17 | 3.96E-15 | Social | Lives with partner |
| p6138 | 1.2755 | 1.2058 | 1.3493 | 2.17E-17 | 4.18E-15 | Social | Lower education |
| p6164_5 | 0.7905 | 0.7472 | 0.8363 | 2.78E-16 | 5.36E-14 | Lifestyle | Heavy DIY |
| p1269 | 1.4003 | 1.2867 | 1.5239 | 6.16E-15 | 1.19E-12 | Chemical | Exposure to tobacco smoke at home |
| p6144_3 | 1.5761 | 1.3993 | 1.7752 | 6.55E-14 | 1.26E-11 | Lifestyle | Never eat wheat products |
| p6145_6 | 1.3931 | 1.272 | 1.5257 | 8.85E-13 | 1.71E-10 | Social | Financial difficulties |
| p709 | 0.89 | 0.8615 | 0.9195 | 2.4E-12 | 4.62E-10 | Social | Number in household |
| p1110 | 0.9405 | 0.9241 | 0.9572 | 8.31E-12 | 1.6E-09 | Lifestyle | Length of mobile phone use |
| p2020 | 1.2593 | 1.1778 | 1.3463 | 1.39E-11 | 2.69E-09 | Social | Loneliness, isolation |
| p1200 | 0.8781 | 0.8456 | 0.9119 | 1.48E-11 | 2.85E-09 | Lifestyle | Experiences insomnia frequently |
| p1239 | 1.1762 | 1.1219 | 1.2332 | 1.74E-11 | 3.36E-09 | Lifestyle | Current tobacco smoking frequency |
| p6164_4 | 0.8358 | 0.7926 | 0.8813 | 3.26E-11 | 6.29E-09 | Lifestyle | Light DIY |
| p924 | 0.8579 | 0.8194 | 0.8983 | 6.52E-11 | 1.26E-08 | Lifestyle | Usually walks quickly |
| p6160_1 | 0.8088 | 0.7576 | 0.8635 | 2.08E-10 | 4.02E-08 | Lifestyle | Attends a sports club or gym frequently |
| p1488 | 0.92 | 0.8966 | 0.944 | 2.28E-10 | 4.39E-08 | Lifestyle | Tea intake |
| p2159 | 1.3456 | 1.2264 | 1.4764 | 3.59E-10 | 6.93E-08 | Lifestyle | Had same-sex intercourse |
| p6144_4 | 1.2058 | 1.1365 | 1.2793 | 5.77E-10 | 1.11E-07 | Lifestyle | Never eat sugar foods |
| p2277 | 1.0966 | 1.065 | 1.1291 | 6.07E-10 | 1.17E-07 | Physical | Uses solarium/sunlamp frequently |
| p1279 | 1.1978 | 1.1306 | 1.269 | 8.96E-10 | 1.73E-07 | Chemical | Exposure to tobacco smoke outside home |
| p2060* | 1.1402 | 1.0933 | 1.1892 | 9.07E-10 | 1.75E-07 | Social | Feels unenthusiasm/disinterest frequently |
| p20116_2 | 1.3302 | 1.2126 | 1.4592 | 1.53E-09 | 2.95E-07 | Lifestyle | Current smoker |
| p1508_2 | 0.787 | 0.7262 | 0.8529 | 5.25E-09 | 1.01E-06 | Lifestyle | Ground coffee versus decaffeinated |
| p900_1 | 1.1753 | 1.1129 | 1.2411 | 6.4E-09 | 1.24E-06 | Lifestyle | Improper consumption of alcohol |
| p24006 | 1.0822 | 1.0536 | 1.1115 | 7.31E-09 | 1.41E-06 | Chemical | PM2.5 pollution |
| p670_1 | 1.2792 | 1.1754 | 1.3922 | 1.17E-08 | 2.25E-06 | Social | Lives in a flat versus house |
| p2139 | 1.1722 | 1.1097 | 1.2383 | 1.31E-08 | 2.52E-06 | Lifestyle | First sexual intercourse (< age 18) |
| p6144_2 | 1.4472 | 1.2711 | 1.6477 | 2.37E-08 | 4.57E-06 | Lifestyle | Never eat dairy products |
| p2400_1 | 1.0809 | 1.051 | 1.1117 | 5.61E-08 | 1.08E-05 | Chemical | Nitrogen dioxide/oxides air pollution |
| p2267 | 0.9236 | 0.8974 | 0.9506 | 6.14E-08 | 1.19E-05 | Physical | Uses sun or UV protection frequently |
| p2050* | 1.1268 | 1.079 | 1.1767 | 6.81E-08 | 1.31E-05 | Social | Feels depressed mood frequently |
| p2000 | 0.8643 | 0.8186 | 0.9126 | 1.44E-07 | 2.78E-05 | Social | Worry too long after embarrassment |
| p1920 | 1.1542 | 1.0932 | 1.2186 | 2.26E-07 | 4.36E-05 | Social | Mood swings |
| p2070 | 1.119 | 1.0718 | 1.1682 | 3.1E-07 | 5.98E-05 | Social | Feels tenseness/restlessness frequently |
| p6154_1 | 1.1659 | 1.0962 | 1.2401 | 1.07E-06 | 0.000207 | Chemical | Usually takes Aspirin |
| p22039 | 0.9365 | 0.912 | 0.9617 | 1.27E-06 | 0.000245 | Lifestyle | MET minutes/week of vigorous activity |
| p6139_3 | 0.7533 | 0.6708 | 0.846 | 1.72E-06 | 0.000332 | Chemical | Uses open solid fuel fire in winter |
| p1220* | 1.1193 | 1.0681 | 1.1729 | 2.37E-06 | 0.000458 | Lifestyle | Daytime sleep frequency |
| p24506 | 0.939 | 0.9145 | 0.9641 | 3.03E-06 | 0.000585 | Ecosystems | Natural environment percentage (1000m buffer) |
| p2110 | 0.9688 | 0.9559 | 0.9818 | 3.17E-06 | 0.000611 | Social | Able to confide frequently |
| p2010 | 1.1645 | 1.0918 | 1.242 | 3.67E-06 | 0.000708 | Social | Suffer from 'nerves' |
| p699 | 0.9396 | 0.9151 | 0.9648 | 3.97E-06 | 0.000767 | Social | Length of residence at current address |
| p6145_1* | 1.2077 | 1.1141 | 1.3091 | 4.54E-06 | 0.000876 | Social | Own serious illness or injury |
| p943 | 0.9533 | 0.9339 | 0.9731 | 4.95E-06 | 0.000956 | Lifestyle | Climbes stair frequently |
| p6155_2 | 1.2998 | 1.1597 | 1.4567 | 6.55E-06 | 0.001264 | Chemical | Vitamin B supplements |
| p6160_4 | 0.7793 | 0.6987 | 0.8693 | 7.66E-06 | 0.001478 | Lifestyle | Attends a adult education class frequently |
| p670_2 | 1.6047 | 1.3031 | 1.976 | 8.45E-06 | 0.001632 | Social | Lives in mobile structure versus house |
| p1548 | 1.1057 | 1.0576 | 1.156 | 9.61E-06 | 0.001855 | Lifestyle | Diet varies frequently |
| p900_7 | 0.8875 | 0.8412 | 0.9363 | 1.27E-05 | 0.002442 | Lifestyle | Wholegrain eaten above recommendation |
| p2400_2 | 1.0608 | 1.0309 | 1.0916 | 5.25E-05 | 0.010124 | Chemical | PM10 pollution |
| p6164_1 | 0.8892 | 0.8399 | 0.9414 | 5.5E-05 | 0.010621 | Lifestyle | Walks for pleasure |
| p1990 | 1.1489 | 1.0738 | 1.2292 | 5.72E-05 | 0.011041 | Social | Tense/'highly strung' |
| p22038* | 0.9499 | 0.926 | 0.9743 | 7.34E-05 | 0.014162 | Lifestyle | MET minutes/week of moderate activity |
| p1980 | 1.1183 | 1.0581 | 1.1818 | 7.36E-05 | 0.014207 | Social | Worrier/anxious feelings |
| p1428_2 | 1.6979 | 1.3066 | 2.2064 | 7.48E-05 | 0.014437 | Lifestyle | Flora Pro-Active spread versus rare/non-spread |
| p2080* | 1.0697 | 1.0343 | 1.1063 | 8.68E-05 | 0.016746 | Social | Feels tiredness/lethargy frequently |
| p1259 | 1.1786 | 1.0847 | 1.2807 | 0.000105 | 0.020268 | Chemical | Had smoking/smokers in household |
| p900_3 | 1.3077 | 1.1414 | 1.4983 | 0.000111 | 0.021363 | Lifestyle | Vegetable eaten above recommendation |
| p900_2 | 1.1203 | 1.0565 | 1.1878 | 0.000144 | 0.027834 | Lifestyle | Improper sleep duration <7/>9 hours |
| p1190 | 1.0852 | 1.0402 | 1.1322 | 0.000155 | 0.030011 | Lifestyle | Naps frequently |
| p1408 | 0.9541 | 0.9308 | 0.9781 | 0.000203 | 0.039258 | Lifestyle | Cheese intake |
| p1359 | 0.9445 | 0.916 | 0.9738 | 0.000252 | 0.048597 | Lifestyle | Poultry intake |
| p6162_3 | 1.1069 | 1.0483 | 1.1689 | 0.000256 | 0.049482 | Lifestyle | Uses public transport |
| p24012 | 1.049 | 1.0219 | 1.0768 | 0.000335 | 0.064628 | Physical | Inverse distance to major road |
| p24508 | 1.051 | 1.0227 | 1.0801 | 0.000353 | 0.068133 | Ecosystems | Distance to coast |
| p6179_5 | 1.3004 | 1.1226 | 1.5062 | 0.00046 | 0.088857 | Chemical | Iron supplements |
| p1767 | 1.3909 | 1.1554 | 1.6744 | 0.00049 | 0.094659 | Social | Adopted as a child |
| p1757 | 1.1006 | 1.0427 | 1.1617 | 0.000507 | 0.097896 | Physical | Degree of facial ageing |
| p6145_2 | 0.8458 | 0.7679 | 0.9317 | 0.000687 | 0.132585 | Social | Relativeâ€™s serious illness or injury |
| p22037* | 0.9577 | 0.9335 | 0.9826 | 0.000967 | 0.186567 | Lifestyle | MET minutes/week of walking |
| p1970 | 1.109 | 1.0428 | 1.1793 | 0.00098 | 0.189101 | Social | Nervous feelings |
| p22034 | 0.9606 | 0.9378 | 0.9839 | 0.001013 | 0.195585 | Lifestyle | Summed minutes activity |
| p6145_5 | 1.3521 | 1.1283 | 1.6203 | 0.001085 | 0.20941 | Social | Marital separation/divorce |
| p6160_5 | 0.9021 | 0.8467 | 0.961 | 0.001412 | 0.272612 | Lifestyle | Attends other group activities frequently |
| p24007 | 1.0436 | 1.0157 | 1.0722 | 0.002003 | 0.38655 | Chemical | PM2.5 absorbance pollution |
| p22033 | 0.962 | 0.9385 | 0.986 | 0.002053 | 0.396243 | Lifestyle | Summed days activity |
| p1518 | 0.9324 | 0.8908 | 0.9759 | 0.002615 | 0.504743 | Lifestyle | Hot drink temperature |
| p6154_4 | 1.2526 | 1.0728 | 1.4626 | 0.004379 | 0.845217 | Chemical | Usually takes Ranitidine |
| p1478 | 1.0436 | 1.0131 | 1.075 | 0.004836 | 0.933422 | Lifestyle | Adds salt to food frequently |
| p6162_4 | 0.8306 | 0.7292 | 0.9461 | 0.005201 | 1 | Lifestyle | Uses cycle as transport |
| p6145_4 | 1.2509 | 1.0648 | 1.4694 | 0.006441 | 1 | Social | Death of a spouse or partner |
| p6154_3 | 1.0917 | 1.0247 | 1.1631 | 0.006662 | 1 | Chemical | Usually takes Paracetamol |
| p1960 | 1.0804 | 1.0215 | 1.1427 | 0.006827 | 1 | Social | Fed-up feelings |
| p1418_4 | 0.7489 | 0.6057 | 0.9261 | 0.007612 | 1 | Lifestyle | Soya milk versus rare/non-milk |
| p1130 | 1.0346 | 1.0084 | 1.0615 | 0.009368 | 1 | Lifestyle | Uses hands-free or speakerphone frequently |
| p6139_2 | 0.9318 | 0.8829 | 0.9833 | 0.010063 | 1 | Chemical | Uses gas fire in winter |
| p1140_3 | 1.2769 | 1.0542 | 1.5467 | 0.012438 | 1 | Lifestyle | Didn't use a mobile phone versus no difference |
| p22040 | 0.9711 | 0.9485 | 0.9943 | 0.015071 | 1 | Lifestyle | MET minutes/week of all activity |
| p6164_3 | 0.8515 | 0.7473 | 0.9702 | 0.015773 | 1 | Lifestyle | Engages in strenuous sports |
| p1210 | 0.9341 | 0.8836 | 0.9874 | 0.016098 | 1 | Lifestyle | Snoring |
| p6162_2 | 0.9371 | 0.8884 | 0.9884 | 0.016952 | 1 | Lifestyle | Walks as transport |
| p1737 | 0.9665 | 0.9397 | 0.994 | 0.017314 | 1 | Physical | Childhood sunburn occurred frequently |
| p864 | 0.9722 | 0.9486 | 0.9963 | 0.024297 | 1 | Lifestyle | Days/week walked 10+ minutes |
| p20116_1 | 1.0657 | 1.0068 | 1.128 | 0.028254 | 1 | Lifestyle | Previous smoker |
| p1538 | 1.0622 | 1.0061 | 1.1213 | 0.029234 | 1 | Lifestyle | Major dietary changes |
| p100_1 | 0.9422 | 0.8928 | 0.9942 | 0.029943 | 1 | Lifestyle | Healthy diet |
| p6155_1 | 1.1898 | 1.016 | 1.3933 | 0.031036 | 1 | Chemical | Vitamin A supplements |
| p6155_6 | 1.1777 | 1.0125 | 1.3698 | 0.033956 | 1 | Chemical | Folic acid or Folate (Vit B9) supplements |
| p900_9 | 0.9417 | 0.89 | 0.9964 | 0.037002 | 1 | Lifestyle | Processed meat intake reduced |
| p24500 | 0.971 | 0.9445 | 0.9982 | 0.037038 | 1 | Ecosystems | Greenspace percentage (1000 m buffer) |
| p900_6 | 0.7937 | 0.638 | 0.9874 | 0.038122 | 1 | Lifestyle | Whole bread/refined cereals intake reduced |
| p6179_2 | 0.9351 | 0.8772 | 0.9968 | 0.039471 | 1 | Chemical | Glucosamine supplements |
| p1528 | 1.0276 | 1.0004 | 1.0556 | 0.046755 | 1 | Lifestyle | Water intake |
| p1150_2 | 1.1449 | 1.0013 | 1.3091 | 0.047786 | 1 | Lifestyle | Prefers right side of head for mobile phone use |
| p1687_2 | 1.0816 | 1.0002 | 1.1697 | 0.049336 | 1 | Social | Relatively plumper at 10 years old |
| p1508_3 | 1.2248 | 0.9986 | 1.5022 | 0.051532 | 1 | Lifestyle | Other coffee types versus decaffeinated |
| p2030 | 0.9416 | 0.8856 | 1.0012 | 0.054543 | 1 | Social | Guilty feelings |
| p1140_2 | 0.9408 | 0.8839 | 1.0014 | 0.055328 | 1 | Lifestyle | More frequent mobile phone use versus no difference |
| p2237 | 0.9492 | 0.8978 | 1.0036 | 0.066742 | 1 | Lifestyle | Plays computer games frequently |
| p6139_1 | 0.9499 | 0.8986 | 1.0042 | 0.069959 | 1 | Chemical | Uses gas hob or cooker |
| p904 | 0.9761 | 0.9506 | 1.0022 | 0.072498 | 1 | Lifestyle | Days/week of vigorous physical activity |
| p884 | 0.9765 | 0.9512 | 1.0024 | 0.074478 | 1 | Lifestyle | Days/week moderate physical activity 10+ minutes |
| p24507 | 0.9763 | 0.95 | 1.0032 | 0.083845 | 1 | Ecosystems | Natural environment percentage (300 m buffer) |
| p20127 | 1.0237 | 0.9968 | 1.0513 | 0.084477 | 1 | Social | Neuroticism score |
| p874 | 1.0217 | 0.9948 | 1.0493 | 0.114962 | 1 | Lifestyle | Duration of walks |
| p1697_2 | 0.9494 | 0.8893 | 1.0135 | 0.119428 | 1 | Social | Relatively taller at 10 years old |
| p1050 | 1.0237 | 0.9937 | 1.0546 | 0.12295 | 1 | Physical | Time spent outdoors (summer) |
| p1707_1 | 0.8719 | 0.7279 | 1.0443 | 0.136448 | 1 | Lifestyle | Right-handed versus ambidextrous |
| p6160_2 | 1.0468 | 0.9854 | 1.1122 | 0.138197 | 1 | Lifestyle | Attends a pub or social club frequently |
| p6179_4 | 0.8996 | 0.7819 | 1.035 | 0.139179 | 1 | Chemical | Zinc supplements |
| p1180 | 0.98 | 0.9539 | 1.0068 | 0.142736 | 1 | Lifestyle | More evening-type chronotype |
| p1120 | 0.9796 | 0.9529 | 1.0071 | 0.144415 | 1 | Lifestyle | Weekly mobile phone usage |
| p1787 | 1.0445 | 0.9847 | 1.1079 | 0.147802 | 1 | Chemical | Maternal smoking around birth |
| p1418_3 | 0.8959 | 0.7682 | 1.0449 | 0.161532 | 1 | Lifestyle | Skimmed milk versus rare/non-milk |
| p24011 | 1.0188 | 0.9924 | 1.046 | 0.1647 | 1 | Physical | Traffic intensity on nearest major road |
| p24008 | 1.0182 | 0.9921 | 1.045 | 0.172744 | 1 | Chemical | PM2.5-10 pollution |
| p1418_2 | 0.905 | 0.782 | 1.0474 | 0.180775 | 1 | Lifestyle | Semi-skimmed milk versus rare/non-milk |
| p900_4 | 1.037 | 0.9828 | 1.0943 | 0.184897 | 1 | Lifestyle | Fruit eaten above recommendation |
| p900_8 | 0.9663 | 0.9155 | 1.0198 | 0.212652 | 1 | Lifestyle | Red meat intake reduced |
| p1060 | 1.0183 | 0.9894 | 1.0481 | 0.217123 | 1 | Physical | Time spent outdoors (winter) |
| p20160 | 1.0355 | 0.9793 | 1.095 | 0.220412 | 1 | Lifestyle | Ever smoked |
| p1940 | 0.9626 | 0.9047 | 1.0242 | 0.228657 | 1 | Social | Irritability |
| p1070 | 1.0188 | 0.988 | 1.0506 | 0.234464 | 1 | Lifestyle | Time spent watching television |
| p6155_4 | 0.9281 | 0.8174 | 1.0537 | 0.248944 | 1 | Chemical | Vitamin D supplements |
| p6160_3 | 0.9603 | 0.8958 | 1.0294 | 0.253304 | 1 | Lifestyle | Attends a religious group frequently |
| p1428_3 | 0.9499 | 0.8673 | 1.0403 | 0.267574 | 1 | Lifestyle | Other spreads/margarine versus rare/non-spread |
| p24503 | 0.9851 | 0.9592 | 1.0118 | 0.27096 | 1 | Ecosystems | Greenspace percentage (300 m buffer) |
| p1707_2 | 0.8994 | 0.737 | 1.0977 | 0.296927 | 1 | Lifestyle | Left-handed versus ambidextrous |
| p1031 | 0.988 | 0.9651 | 1.0115 | 0.315225 | 1 | Social | Friend/family visits frequently |
| p24502 | 1.0135 | 0.9872 | 1.0405 | 0.318066 | 1 | Ecosystems | Water percentage (1000 m buffer) |
| p24020 | 1.0135 | 0.9871 | 1.0405 | 0.319892 | 1 | Physical | Average noise level (daytime) |
| p24021 | 1.0134 | 0.9871 | 1.0405 | 0.32067 | 1 | Physical | Average noise level (evening) |
| p24022 | 1.0134 | 0.9871 | 1.0404 | 0.322162 | 1 | Physical | Average noise level (nighttime) |
| p1508_1 | 0.9684 | 0.9086 | 1.0322 | 0.324064 | 1 | Lifestyle | Instant coffee versus decaffeinated |
| p1428_1 | 0.9541 | 0.8677 | 1.0492 | 0.332424 | 1 | Lifestyle | Butter/spreadable butter versus rare/non-spread |
| p1930 | 1.0269 | 0.9714 | 1.0855 | 0.348992 | 1 | Social | Miserableness |
| p1697_1 | 1.0327 | 0.9653 | 1.1047 | 0.349814 | 1 | Social | Relatively shorter at 10 years old |
| p1418_5 | 1.1247 | 0.8686 | 1.4561 | 0.37271 | 1 | Lifestyle | Other milk types versus rare/non-milk |
| p6155_3 | 1.0414 | 0.9525 | 1.1385 | 0.373096 | 1 | Chemical | Vitamin C supplements |
| p24009 | 1.0114 | 0.9855 | 1.038 | 0.390384 | 1 | Physical | Traffic intensity on nearest road |
| p22036 | 1.0344 | 0.9574 | 1.1175 | 0.391595 | 1 | Lifestyle | At or above moderate/vigorous/walking recommendation |
| p22032 | 0.9847 | 0.9499 | 1.0207 | 0.400451 | 1 | Lifestyle | IPAQ activity level |
| p1677 | 0.9751 | 0.9172 | 1.0367 | 0.420052 | 1 | Chemical | Breastfed as a baby |
| p6154_2 | 0.9655 | 0.8856 | 1.0526 | 0.42504 | 1 | Chemical | Usually takes Ibuprofen |
| p24014 | 1.0419 | 0.941 | 1.1536 | 0.429836 | 1 | Physical | Close to major road |
| p2040 | 1.0246 | 0.9624 | 1.0909 | 0.446465 | 1 | Social | Risk taking |
| p6155_5 | 1.053 | 0.9136 | 1.2137 | 0.475585 | 1 | Chemical | Vitamin E supplements |
| p6179_3 | 1.0351 | 0.9407 | 1.1391 | 0.479169 | 1 | Chemical | Calcium supplements |
| p1140_1 | 1.0311 | 0.9463 | 1.1234 | 0.484251 | 1 | Lifestyle | Less frequent mobile phone use versus no difference |
| p1418_1 | 1.0615 | 0.8913 | 1.2643 | 0.50312 | 1 | Lifestyle | Full-cream milk versus rare/non-milk |
| p22035 | 0.9819 | 0.9307 | 1.036 | 0.505107 | 1 | Lifestyle | At or above moderate/vigorous recommendation |
| p24505 | 0.9915 | 0.9657 | 1.018 | 0.525418 | 1 | Ecosystems | Water percentage (300 m buffer) |
| p6154_5 | 1.0273 | 0.9386 | 1.1243 | 0.559256 | 1 | Chemical | Usually takes Omeprazole |
| p900_5 | 0.9797 | 0.9146 | 1.0495 | 0.559395 | 1 | Lifestyle | Fish eaten above recommendation |
| p1150_1 | 1.0413 | 0.9056 | 1.1973 | 0.569681 | 1 | Lifestyle | Prefers left side of head for mobile phone use |
| p2149 | 1.0075 | 0.9781 | 1.0377 | 0.620972 | 1 | Lifestyle | Lifetime number of sexual partners |
| p6179_6 | 1.0333 | 0.8821 | 1.2105 | 0.684553 | 1 | Chemical | Selenium supplements |
| p1950 | 1.0107 | 0.9572 | 1.0671 | 0.701618 | 1 | Social | Sensitivity/hurt feelings |
| p6179_1 | 0.9904 | 0.9378 | 1.046 | 0.729518 | 1 | Chemical | Fish oil (including cod liver oil) supplements |
| p1687_1 | 1.0092 | 0.9519 | 1.07 | 0.758455 | 1 | Social | Relatively thinner at 10 years old |
| p6155_7 | 1.0096 | 0.946 | 1.0775 | 0.772484 | 1 | Chemical | Multivitamins +/- minerals supplements |
| p894 | 0.9962 | 0.9704 | 1.0227 | 0.775689 | 1 | Lifestyle | Duration of moderate activity |
| p24504 | 0.9967 | 0.9705 | 1.0237 | 0.808918 | 1 | Ecosystems | Domestic garden percentage (300 m buffer) |
| p24010 | 0.9978 | 0.9716 | 1.0246 | 0.868557 | 1 | Physical | Inverse distance to road |
| p1170 | 0.9973 | 0.962 | 1.0338 | 0.882092 | 1 | Lifestyle | Gets up more easily in the morning |
| p1498 | 1.002 | 0.9749 | 1.0299 | 0.885623 | 1 | Lifestyle | Coffee intake |
| p981 | 1.001 | 0.9861 | 1.016 | 0.899051 | 1 | Lifestyle | Duration of walking for pleasure |
| p24501 | 1.0013 | 0.9754 | 1.0278 | 0.923175 | 1 | Ecosystems | Domestic garden percentage (1000 m buffer) |
| p1249 | 0.9993 | 0.9787 | 1.0205 | 0.950544 | 1 | Lifestyle | Past tobacco smoking frequency |
| p6145_3 | 0.9996 | 0.936 | 1.0675 | 0.989703 | 1 | Social | Death of a close relative |

**Notes:** ADRD = Alzheimer’s Disease and Related Dementias. ExWAS=Exposome-wide association studies. This table presents the ExWAS results from the derivation set (N = 333,328; Cases = 5,926; Controls = 327,402).

* This variable violated the proportional hazards assumption (Schoenfeld residuals P < 0.0001). The HR presented here represents the average hazard ratio over the entire follow-up period. Time-varying effects are detailed in Supplementary Table 12.

**Supplementary Table 8. Associations between 193 exposures and AD in ExWAS**

| **Variable code** | **HR** | **Lower confidence interval of HR** | **Upper confidence interval of HR** | **P** | **P**  **Bonferroni**  **adjust** | **Category** | **Variable** |
| --- | --- | --- | --- | --- | --- | --- | --- |
| p1080 | 0.8367 | 0.8069 | 0.8677 | 6.69E-22 | 1.29E-19 | Lifestyle | Time spent using computer |
| p1100 | 0.7253 | 0.6777 | 0.7763 | 1.95E-20 | 3.77E-18 | Lifestyle | Frequent motorway speeding |
| p738 | 0.8489 | 0.8156 | 0.8835 | 9.51E-16 | 1.84E-13 | Social | Total household income |
| p6144_1 | 1.9393 | 1.6488 | 2.2811 | 1.26E-15 | 2.44E-13 | Lifestyle | Never eat eggs |
| p6146 | 1.675 | 1.4742 | 1.903 | 2.40E-15 | 4.63E-13 | Social | Disability |
| p6138 | 1.3909 | 1.2778 | 1.514 | 2.41E-14 | 4.64E-12 | Social | Lower education |
| p728 | 0.8111 | 0.7683 | 0.8563 | 3.98E-14 | 7.68E-12 | Social | Number of vehicles in household |
| p680 | 1.5796 | 1.3989 | 1.7836 | 1.62E-13 | 3.13E-11 | Social | Rent accommodation versus own |
| p6144_3 | 1.8135 | 1.5335 | 2.1446 | 3.49E-12 | 6.74E-10 | Lifestyle | Never eat wheat products |
| p1200 | 0.8273 | 0.7819 | 0.8754 | 4.89E-11 | 9.44E-09 | Lifestyle | Experiences insomnia frequently |
| p6162_1 | 0.7477 | 0.6848 | 0.8164 | 8.85E-11 | 1.71E-08 | Lifestyle | Uses car/motor vehicle as transport |
| p2000 | 0.7717 | 0.7108 | 0.8378 | 6.48E-10 | 1.25E-07 | Social | Worry too long after embarrassment |
| p6144_4 | 1.278 | 1.1713 | 1.3944 | 3.52E-08 | 6.79E-06 | Lifestyle | Never eat sugar foods |
| p1508_2 | 0.7268 | 0.6445 | 0.8196 | 1.95E-07 | 3.76E-05 | Lifestyle | Ground coffee versus decaffeinated |
| p1090 | 0.9046 | 0.8707 | 0.9397 | 2.51E-07 | 4.85E-05 | Lifestyle | Time spent driving |
| p6164_2 | 0.8054 | 0.7413 | 0.8751 | 3.18E-07 | 6.14E-05 | Lifestyle | Engages in other exercises |
| p6164_4 | 0.8143 | 0.7524 | 0.8814 | 3.61E-07 | 6.98E-05 | Lifestyle | Light DIY |
| p1110 | 0.9357 | 0.9115 | 0.9605 | 6.63E-07 | 0.000128 | Lifestyle | Length of mobile phone use |
| p6164_5 | 0.8136 | 0.7484 | 0.8846 | 1.33E-06 | 0.000257 | Lifestyle | Heavy DIY |
| p24006 | 1.1024 | 1.0594 | 1.1472 | 1.54E-06 | 0.000298 | Chemical | PM2.5 pollution |
| p2400_1 | 1.1021 | 1.0571 | 1.1491 | 4.97E-06 | 0.000959 | Chemical | Nitrogen dioxide/oxides air pollution |
| p6142 | 1.5519 | 1.2841 | 1.8756 | 5.44E-06 | 0.001051 | Social | Unemployed |
| p2492 | 1.2191 | 1.1176 | 1.3298 | 7.92E-06 | 0.001529 | Chemical | Takes other prescription medications |
| p1269 | 1.3418 | 1.1774 | 1.5291 | 1.04E-05 | 0.002005 | Chemical | Exposure to tobacco smoke at home |
| p6141 | 0.8276 | 0.7588 | 0.9026 | 1.91E-05 | 0.003686 | Social | Lives with partner |
| p1548 | 1.1552 | 1.0808 | 1.2346 | 2.14E-05 | 0.004135 | Lifestyle | Diet varies frequently |
| p6160_1 | 0.8136 | 0.7389 | 0.8958 | 2.68E-05 | 0.005167 | Lifestyle | Attends a sports club or gym frequently |
| p1488 | 0.9219 | 0.8868 | 0.9583 | 3.92E-05 | 0.007562 | Lifestyle | Tea intake |
| p6144_2 | 1.4893 | 1.2295 | 1.8042 | 4.67E-05 | 0.009021 | Lifestyle | Never eat dairy products |
| p2159 | 1.3283 | 1.1543 | 1.5286 | 7.4E-05 | 0.014276 | Lifestyle | Had same-sex intercourse |
| p137 | 1.1085 | 1.053 | 1.167 | 8.42E-05 | 0.016256 | Chemical | Number of medications taken |
| p2060 | 1.1371 | 1.0652 | 1.2139 | 0.000115 | 0.022275 | Social | Feels unenthusiasm/disinterest frequently |
| p900_1 | 1.173 | 1.0813 | 1.2725 | 0.000122 | 0.023579 | Lifestyle | Improper consumption of alcohol |
| p6154_6 | 1.4112 | 1.1809 | 1.6865 | 0.000152 | 0.029243 | Chemical | Usually takes Laxatives |
| p6145_6 | 1.3055 | 1.1287 | 1.51 | 0.000329 | 0.063501 | Social | Financial difficulties |
| p6160_4 | 0.7452 | 0.6342 | 0.8755 | 0.000348 | 0.067194 | Lifestyle | Attends a adult education class frequently |
| p2139 | 1.1599 | 1.0681 | 1.2596 | 0.000422 | 0.08136 | Lifestyle | First sexual intercourse (< age 18) |
| p20116_2 | 1.2944 | 1.1204 | 1.4954 | 0.00046 | 0.088733 | Lifestyle | Current smoker |
| p1239 | 1.1411 | 1.0586 | 1.2301 | 0.00057 | 0.110042 | Lifestyle | Current tobacco smoking frequency |
| p2050 | 1.1207 | 1.0474 | 1.1991 | 0.000954 | 0.184167 | Social | Feels depressed mood frequently |
| p943 | 0.9505 | 0.9219 | 0.9798 | 0.001073 | 0.207085 | Lifestyle | Climbes stair frequently |
| p6139_3 | 0.7514 | 0.6327 | 0.8925 | 0.00113 | 0.218135 | Chemical | Uses open solid fuel fire in winter |
| p1528 | 1.0734 | 1.0282 | 1.1205 | 0.001242 | 0.239752 | Lifestyle | Water intake |
| p2277 | 1.0763 | 1.0288 | 1.126 | 0.001412 | 0.27254 | Physical | Uses solarium/sunlamp frequently |
| p2070 | 1.1133 | 1.0412 | 1.1905 | 0.001691 | 0.326304 | Social | Feels tenseness/restlessness frequently |
| p2020 | 1.1767 | 1.0616 | 1.3043 | 0.001951 | 0.376523 | Social | Loneliness, isolation |
| p709 | 0.9266 | 0.8819 | 0.9735 | 0.002501 | 0.482657 | Social | Number in household |
| p1279 | 1.1449 | 1.0486 | 1.2499 | 0.002524 | 0.487139 | Chemical | Exposure to tobacco smoke outside home |
| p1259 | 1.2043 | 1.0625 | 1.365 | 0.003621 | 0.698944 | Chemical | Had smoking/smokers in household |
| p1980 | 1.1278 | 1.0384 | 1.2248 | 0.00432 | 0.833806 | Social | Worrier/anxious feelings |
| p24007 | 1.06 | 1.0183 | 1.1034 | 0.004415 | 0.85211 | Chemical | PM2.5 absorbance pollution |
| p24506 | 0.9449 | 0.9081 | 0.9831 | 0.005075 | 0.979447 | Ecosystems | Natural environment percentage (1000m buffer) |
| p1920 | 1.1221 | 1.0345 | 1.2171 | 0.005478 | 1 | Social | Mood swings |
| p2110 | 0.9721 | 0.9528 | 0.9919 | 0.00594 | 1 | Social | Able to confide frequently |
| p6160_5 | 0.8786 | 0.8002 | 0.9646 | 0.006584 | 1 | Lifestyle | Attends other group activities frequently |
| p22036 | 1.1872 | 1.048 | 1.3449 | 0.006986 | 1 | Lifestyle | At or above moderate/vigorous/walking recommendation |
| p1140_2 | 0.8841 | 0.805 | 0.9711 | 0.01006 | 1 | Lifestyle | More frequent mobile phone use versus no difference |
| p6162_4 | 0.7725 | 0.6326 | 0.9432 | 0.011297 | 1 | Lifestyle | Uses cycle as transport |
| p1757 | 1.1109 | 1.0233 | 1.2059 | 0.012038 | 1 | Physical | Degree of facial ageing |
| p1518 | 0.9171 | 0.8565 | 0.982 | 0.013106 | 1 | Lifestyle | Hot drink temperature |
| p6145_2 | 0.8336 | 0.7209 | 0.9639 | 0.014067 | 1 | Social | Relativeâ€™s serious illness or injury |
| p2030 | 0.8914 | 0.8122 | 0.9783 | 0.015386 | 1 | Social | Guilty feelings |
| p24505 | 0.9542 | 0.9176 | 0.9922 | 0.018745 | 1 | Ecosystems | Water percentage (300 m buffer) |
| p2267 | 0.9497 | 0.9095 | 0.9916 | 0.019224 | 1 | Physical | Uses sun or UV protection frequently |
| p6154_1 | 1.1182 | 1.018 | 1.2282 | 0.019632 | 1 | Chemical | Usually takes Aspirin |
| p900_4 | 1.1004 | 1.0149 | 1.193 | 0.020359 | 1 | Lifestyle | Fruit eaten above recommendation |
| p1787 | 1.1082 | 1.0154 | 1.2095 | 0.021301 | 1 | Chemical | Maternal smoking around birth |
| p6179_5 | 1.2949 | 1.0372 | 1.6166 | 0.022449 | 1 | Chemical | Iron supplements |
| p670_1 | 1.1661 | 1.0217 | 1.3309 | 0.022723 | 1 | Social | Lives in a flat versus house |
| p670_2 | 1.4506 | 1.0425 | 2.0183 | 0.027315 | 1 | Social | Lives in mobile structure versus house |
| p1359 | 0.9508 | 0.9082 | 0.9954 | 0.030985 | 1 | Lifestyle | Poultry intake |
| p900_6 | 0.6825 | 0.4814 | 0.9677 | 0.031986 | 1 | Lifestyle | Whole bread/refined cereals intake reduced |
| p900_7 | 0.9165 | 0.8457 | 0.9932 | 0.033517 | 1 | Lifestyle | Wholegrain eaten above recommendation |
| p1060 | 1.0511 | 1.0037 | 1.1008 | 0.03439 | 1 | Physical | Time spent outdoors (winter) |
| p2040 | 0.9021 | 0.8179 | 0.9949 | 0.039167 | 1 | Social | Risk taking |
| p6155_4 | 0.8137 | 0.6681 | 0.9909 | 0.040315 | 1 | Chemical | Vitamin D supplements |
| p900_3 | 1.2443 | 1.0089 | 1.5347 | 0.041079 | 1 | Lifestyle | Vegetable eaten above recommendation |
| p6155_3 | 1.1354 | 0.999 | 1.2906 | 0.051901 | 1 | Chemical | Vitamin C supplements |
| p1050 | 1.0464 | 0.999 | 1.096 | 0.055201 | 1 | Physical | Time spent outdoors (summer) |
| p1408 | 0.9652 | 0.9301 | 1.0016 | 0.06087 | 1 | Lifestyle | Cheese intake |
| p1210 | 0.9235 | 0.8494 | 1.0041 | 0.062428 | 1 | Lifestyle | Snoring |
| p6154_4 | 1.2512 | 0.9875 | 1.5853 | 0.063445 | 1 | Chemical | Usually takes Ranitidine |
| p2400_2 | 1.0403 | 0.9971 | 1.0854 | 0.067653 | 1 | Chemical | PM10 pollution |
| p6155_7 | 1.0915 | 0.993 | 1.1997 | 0.069524 | 1 | Chemical | Multivitamins +/- minerals supplements |
| p1737 | 0.9617 | 0.9219 | 1.0032 | 0.070119 | 1 | Physical | Childhood sunburn occurred frequently |
| p2080 | 1.0472 | 0.9943 | 1.1031 | 0.081333 | 1 | Social | Feels tiredness/lethargy frequently |
| p6179_1 | 1.0736 | 0.9907 | 1.1634 | 0.083429 | 1 | Chemical | Fish oil (including cod liver oil) supplements |
| p6179_4 | 0.8286 | 0.6681 | 1.0276 | 0.086818 | 1 | Chemical | Zinc supplements |
| p1150_2 | 1.1946 | 0.9731 | 1.4665 | 0.089319 | 1 | Lifestyle | Prefers right side of head for mobile phone use |
| p2149 | 0.9623 | 0.9195 | 1.0072 | 0.098799 | 1 | Lifestyle | Lifetime number of sexual partners |
| p24012 | 1.0332 | 0.9935 | 1.0745 | 0.101891 | 1 | Physical | Inverse distance to major road |
| p22039 | 0.968 | 0.9304 | 1.0071 | 0.107358 | 1 | Lifestyle | MET minutes/week of vigorous activity |
| p874 | 1.0334 | 0.9928 | 1.0757 | 0.108178 | 1 | Lifestyle | Duration of walks |
| p20116_1 | 1.07 | 0.9835 | 1.164 | 0.115528 | 1 | Lifestyle | Previous smoker |
| p900_9 | 0.9354 | 0.8603 | 1.017 | 0.117637 | 1 | Lifestyle | Processed meat intake reduced |
| p24500 | 0.9681 | 0.9292 | 1.0088 | 0.122653 | 1 | Ecosystems | Greenspace percentage (1000 m buffer) |
| p1697_2 | 0.9266 | 0.84 | 1.0222 | 0.128268 | 1 | Social | Relatively taller at 10 years old |
| p699 | 0.9692 | 0.9307 | 1.0092 | 0.129677 | 1 | Social | Length of residence at current address |
| p6164_3 | 0.8628 | 0.7119 | 1.0457 | 0.132494 | 1 | Lifestyle | Engages in strenuous sports |
| p1428_1 | 0.8986 | 0.7814 | 1.0334 | 0.133852 | 1 | Lifestyle | Butter/spreadable butter versus rare/non-spread |
| p6145_4 | 1.1986 | 0.9414 | 1.5259 | 0.14155 | 1 | Social | Death of a spouse or partner |
| p1767 | 1.246 | 0.9278 | 1.6734 | 0.143734 | 1 | Social | Adopted as a child |
| p1418_4 | 0.7896 | 0.5698 | 1.0942 | 0.155773 | 1 | Lifestyle | Soya milk versus rare/non-milk |
| p1418_5 | 1.3135 | 0.8903 | 1.9377 | 0.169292 | 1 | Lifestyle | Other milk types versus rare/non-milk |
| p1130 | 1.0276 | 0.9884 | 1.0684 | 0.170068 | 1 | Lifestyle | Uses hands-free or speakerphone frequently |
| p900_2 | 1.0635 | 0.973 | 1.1625 | 0.174645 | 1 | Lifestyle | Improper sleep duration <7/>9 hours |
| p6155_2 | 1.1321 | 0.9453 | 1.3557 | 0.177444 | 1 | Chemical | Vitamin B supplements |
| p24508 | 1.0277 | 0.9876 | 1.0695 | 0.178891 | 1 | Ecosystems | Distance to coast |
| p6154_2 | 0.9137 | 0.8004 | 1.0431 | 0.181814 | 1 | Chemical | Usually takes Ibuprofen |
| p1170 | 1.0379 | 0.9821 | 1.0968 | 0.187029 | 1 | Lifestyle | Gets up more easily in the morning |
| p1508_1 | 0.9395 | 0.8552 | 1.032 | 0.192879 | 1 | Lifestyle | Instant coffee versus decaffeinated |
| p1428_2 | 1.3258 | 0.8565 | 2.0523 | 0.205876 | 1 | Lifestyle | Flora Pro-Active spread versus rare/non-spread |
| p6154_3 | 1.0638 | 0.9665 | 1.1708 | 0.206148 | 1 | Chemical | Usually takes Paracetamol |
| p24502 | 0.9761 | 0.9392 | 1.0145 | 0.219973 | 1 | Ecosystems | Water percentage (1000 m buffer) |
| p1990 | 1.0666 | 0.961 | 1.1838 | 0.22571 | 1 | Social | Tense/'highly strung' |
| p2010 | 1.0637 | 0.9623 | 1.1757 | 0.227052 | 1 | Social | Suffer from 'nerves' |
| p864 | 1.0248 | 0.9802 | 1.0714 | 0.280543 | 1 | Lifestyle | Days/week walked 10+ minutes |
| p1538 | 1.0447 | 0.9632 | 1.133 | 0.291218 | 1 | Lifestyle | Major dietary changes |
| p1940 | 0.9505 | 0.8649 | 1.0446 | 0.292268 | 1 | Social | Irritability |
| p6139_2 | 0.9584 | 0.8846 | 1.0384 | 0.299125 | 1 | Chemical | Uses gas fire in winter |
| p1418_1 | 1.1534 | 0.8759 | 1.5189 | 0.309352 | 1 | Lifestyle | Full-cream milk versus rare/non-milk |
| p904 | 1.02 | 0.9803 | 1.0613 | 0.328697 | 1 | Lifestyle | Days/week of vigorous physical activity |
| p2237 | 0.9595 | 0.883 | 1.0427 | 0.33029 | 1 | Lifestyle | Plays computer games frequently |
| p6162_2 | 0.9622 | 0.8887 | 1.0417 | 0.341305 | 1 | Lifestyle | Walks as transport |
| p22032 | 1.0264 | 0.9722 | 1.0836 | 0.346801 | 1 | Lifestyle | IPAQ activity level |
| p22040 | 1.0196 | 0.979 | 1.062 | 0.348471 | 1 | Lifestyle | MET minutes/week of all activity |
| p1220 | 1.0342 | 0.9617 | 1.1121 | 0.364593 | 1 | Lifestyle | Daytime sleep frequency |
| p1697_1 | 1.0472 | 0.9473 | 1.1575 | 0.367383 | 1 | Social | Relatively shorter at 10 years old |
| p6155_1 | 1.1131 | 0.8744 | 1.4168 | 0.384329 | 1 | Chemical | Vitamin A supplements |
| p24011 | 1.0171 | 0.9779 | 1.0579 | 0.397869 | 1 | Physical | Traffic intensity on nearest major road |
| p6164_1 | 0.9631 | 0.8828 | 1.0508 | 0.398057 | 1 | Lifestyle | Walks for pleasure |
| p1180 | 0.983 | 0.944 | 1.0236 | 0.406455 | 1 | Lifestyle | More evening-type chronotype |
| p22038 | 0.9835 | 0.9451 | 1.0234 | 0.412749 | 1 | Lifestyle | MET minutes/week of moderate activity |
| p1508_3 | 1.137 | 0.829 | 1.5593 | 0.425692 | 1 | Lifestyle | Other coffee types versus decaffeinated |
| p20160 | 1.0332 | 0.9511 | 1.1224 | 0.439004 | 1 | Lifestyle | Ever smoked |
| p1428_3 | 0.9514 | 0.8331 | 1.0864 | 0.461503 | 1 | Lifestyle | Other spreads/margarine versus rare/non-spread |
| p24008 | 1.0145 | 0.9759 | 1.0545 | 0.467503 | 1 | Chemical | PM2.5-10 pollution |
| p1140_3 | 1.1122 | 0.8214 | 1.5059 | 0.491762 | 1 | Lifestyle | Didn't use a mobile phone versus no difference |
| p6145_5 | 1.1144 | 0.8176 | 1.5188 | 0.493058 | 1 | Social | Marital separation/divorce |
| p1687_2 | 1.0413 | 0.9253 | 1.1719 | 0.501987 | 1 | Social | Relatively plumper at 10 years old |
| p894 | 1.0134 | 0.974 | 1.0544 | 0.509898 | 1 | Lifestyle | Duration of moderate activity |
| p6155_6 | 1.0814 | 0.8534 | 1.3703 | 0.517103 | 1 | Chemical | Folic acid or Folate (Vit B9) supplements |
| p6139_1 | 1.0274 | 0.9448 | 1.1171 | 0.527353 | 1 | Chemical | Uses gas hob or cooker |
| p1190 | 0.9799 | 0.9185 | 1.0453 | 0.537719 | 1 | Lifestyle | Naps frequently |
| p6145_1 | 0.9583 | 0.8369 | 1.0974 | 0.53799 | 1 | Social | Own serious illness or injury |
| p1031 | 1.0112 | 0.9757 | 1.0479 | 0.54203 | 1 | Social | Friend/family visits frequently |
| p900_8 | 0.976 | 0.9006 | 1.0576 | 0.552985 | 1 | Lifestyle | Red meat intake reduced |
| p924 | 0.9795 | 0.9143 | 1.0493 | 0.554809 | 1 | Lifestyle | Usually walks quickly |
| p1120 | 0.9875 | 0.947 | 1.0297 | 0.555866 | 1 | Lifestyle | Weekly mobile phone usage |
| p1478 | 1.0134 | 0.9685 | 1.0605 | 0.564361 | 1 | Lifestyle | Adds salt to food frequently |
| p6145_3 | 1.029 | 0.933 | 1.135 | 0.567316 | 1 | Social | Death of a close relative |
| p981 | 0.9937 | 0.9716 | 1.0163 | 0.582678 | 1 | Lifestyle | Duration of walking for pleasure |
| p24014 | 0.9588 | 0.8183 | 1.1233 | 0.602417 | 1 | Physical | Close to major road |
| p1140_1 | 0.9672 | 0.8471 | 1.1043 | 0.621961 | 1 | Lifestyle | Less frequent mobile phone use versus no difference |
| p1950 | 0.9806 | 0.9043 | 1.0633 | 0.634673 | 1 | Social | Sensitivity/hurt feelings |
| p22034 | 1.0103 | 0.9683 | 1.0541 | 0.636132 | 1 | Lifestyle | Summed minutes activity |
| p1070 | 1.0106 | 0.966 | 1.0573 | 0.646353 | 1 | Lifestyle | Time spent watching television |
| p884 | 1.0097 | 0.9687 | 1.0525 | 0.648268 | 1 | Lifestyle | Days/week moderate physical activity 10+ minutes |
| p22035 | 1.0188 | 0.94 | 1.1042 | 0.649677 | 1 | Lifestyle | At or above moderate/vigorous recommendation |
| p6179_2 | 1.021 | 0.9314 | 1.1193 | 0.656729 | 1 | Chemical | Glucosamine supplements |
| p6160_3 | 0.9785 | 0.8841 | 1.083 | 0.674565 | 1 | Lifestyle | Attends a religious group frequently |
| p1150_1 | 1.0454 | 0.8443 | 1.2944 | 0.683668 | 1 | Lifestyle | Prefers left side of head for mobile phone use |
| p1970 | 1.0197 | 0.9281 | 1.1203 | 0.684911 | 1 | Social | Nervous feelings |
| p6179_6 | 0.9516 | 0.7464 | 1.2131 | 0.688661 | 1 | Chemical | Selenium supplements |
| p1498 | 1.0081 | 0.9672 | 1.0508 | 0.701416 | 1 | Lifestyle | Coffee intake |
| p1707_2 | 1.0639 | 0.7689 | 1.4721 | 0.708359 | 1 | Lifestyle | Left-handed versus ambidextrous |
| p24501 | 1.007 | 0.9684 | 1.0472 | 0.727404 | 1 | Ecosystems | Domestic garden percentage (1000 m buffer) |
| p1249 | 1.0055 | 0.9745 | 1.0374 | 0.731836 | 1 | Lifestyle | Past tobacco smoking frequency |
| p1707_1 | 1.0524 | 0.7808 | 1.4185 | 0.73746 | 1 | Lifestyle | Right-handed versus ambidextrous |
| p1677 | 0.985 | 0.8985 | 1.0797 | 0.746252 | 1 | Chemical | Breastfed as a baby |
| p900_5 | 0.9831 | 0.8859 | 1.091 | 0.749016 | 1 | Lifestyle | Fish eaten above recommendation |
| p6155_5 | 0.9658 | 0.7768 | 1.2008 | 0.754382 | 1 | Chemical | Vitamin E supplements |
| p24503 | 0.9939 | 0.9551 | 1.0343 | 0.763125 | 1 | Ecosystems | Greenspace percentage (300 m buffer) |
| p1960 | 1.0126 | 0.9304 | 1.1022 | 0.771417 | 1 | Social | Fed-up feelings |
| p1687_1 | 0.9879 | 0.9051 | 1.0781 | 0.784252 | 1 | Social | Relatively thinner at 10 years old |
| p6154_5 | 1.0186 | 0.8873 | 1.1692 | 0.793889 | 1 | Chemical | Usually takes Omeprazole |
| p24009 | 0.9959 | 0.9569 | 1.0365 | 0.841894 | 1 | Physical | Traffic intensity on nearest road |
| p6160_2 | 1.0088 | 0.92 | 1.1063 | 0.851589 | 1 | Lifestyle | Attends a pub or social club frequently |
| p6162_3 | 1.0078 | 0.9287 | 1.0936 | 0.852492 | 1 | Lifestyle | Uses public transport |
| p22033 | 1.004 | 0.9608 | 1.0492 | 0.859087 | 1 | Lifestyle | Summed days activity |
| p24022 | 1.0033 | 0.9642 | 1.0441 | 0.869195 | 1 | Physical | Average noise level (nighttime) |
| p1930 | 0.993 | 0.9136 | 1.0794 | 0.8692 | 1 | Social | Miserableness |
| p24021 | 1.0033 | 0.9641 | 1.044 | 0.872343 | 1 | Physical | Average noise level (evening) |
| p24020 | 1.0032 | 0.964 | 1.0439 | 0.875585 | 1 | Physical | Average noise level (daytime) |
| p6179_3 | 1.0087 | 0.8764 | 1.161 | 0.903838 | 1 | Chemical | Calcium supplements |
| p1418_2 | 1.0143 | 0.8056 | 1.277 | 0.903975 | 1 | Lifestyle | Semi-skimmed milk versus rare/non-milk |
| p22037 | 1.0024 | 0.9596 | 1.0471 | 0.914009 | 1 | Lifestyle | MET minutes/week of walking |
| p100_1 | 0.996 | 0.9197 | 1.0786 | 0.921167 | 1 | Lifestyle | Healthy diet |
| p24010 | 1.002 | 0.963 | 1.0426 | 0.921292 | 1 | Physical | Inverse distance to road |
| p20127 | 1.0015 | 0.9634 | 1.0411 | 0.938381 | 1 | Social | Neuroticism score |
| p1418_3 | 1.0088 | 0.793 | 1.2834 | 0.943023 | 1 | Lifestyle | Skimmed milk versus rare/non-milk |
| p24507 | 1.0012 | 0.9603 | 1.0438 | 0.95542 | 1 | Ecosystems | Natural environment percentage (300 m buffer) |
| p24504 | 0.9998 | 0.9608 | 1.0404 | 0.992219 | 1 | Ecosystems | Domestic garden percentage (300 m buffer) |

**Notes:** AD = Alzheimer’s Disease. ExWAS=Exposome-wide association studies. This table presents the ExWAS results from the derivation set (N = 330,076; 2,674 cases and 327,402 controls).

**Supplementary Table 9.** Time-dependent effects of the exposures on incident ADRD

| **Variable code** | **Average HR (from original model)** | **Main HR (at earliest follow-up)** | **P-value (Main)** | **Interaction HR (Exposure × log-time)** | **P-value (Interaction)** | **Variable** |
| --- | --- | --- | --- | --- | --- | --- |
| p2060 | 1.1402 | 1.4664 | 2.36E-13 | 0.8801 | 3.14E-07 | Feels unenthusiasm/disinterest frequently |
| p2050 | 1.1268 | 1.4525 | 7.16E-12 | 0.8794 | 7.33E-07 | Feels depressed mood frequently |
| p1220 | 1.1193 | 1.4997 | 1.97E-09 | 0.8645 | 4.68E-06 | Daytime sleep frequency |
| p6145_1 | 1.2077 | 1.9315 | 6.23E-10 | 0.7883 | 2.82E-06 | Own serious illness or injury |
| p22038 | 0.9499 | 0.8355 | 9.59E-07 | 1.0661 | 0.000217983 | MET minutes/week of moderate activity |
| p2080 | 1.0697 | 1.2701 | 9.58E-08 | 0.918 | 4.08E-05 | Feels tiredness/lethargy frequently |
| p22037 | 0.9577 | 0.8422 | 4.45E-08 | 1.068 | 1.42E-05 | MET minutes/week of walking |

**Notes:** Average HR is derived from the standard Cox proportional hazards model without time interaction. For these variables, the proportional hazards assumption was significantly violated (Schoenfeld residuals P < 0.0001), indicating that their effect sizes changed over the follow-up period. To appropriately model this, a time-dependent interaction term [Exposure × log(time)] was included. Main HR represents the estimated hazard ratio at the earliest follow-up time. Interaction HR represents the multiplicative change in the main hazard ratio per unit increase in log-time. ADRD: Alzheimer's Disease and Related Dementias; HR: Hazard Ratio.

**Supplementary Table 10. Associations between 193 exposures and ADRD in subgroups (by sex, age, genetics, and ethnicity)**

| **Variable Code** | **Female** | | **Male** | | **Age<65** | | **Age>=65** | | **Low genetic risk** | | **Intermediate genetic risk** | | **High genetic risk** | | **White British** | | **Other ethnicities** | | **Category** | **Variable** |
| --- | --- | --- | --- | --- | --- | --- | --- | --- | --- | --- | --- | --- | --- | --- | --- | --- | --- | --- | --- | --- |
|  | **HR** | **P** | **HR** | **P** | **HR** | **P** | **HR** | **P** | **HR** | **P** | **HR** | **P** | **HR** | **P** | **HR** | **P** | **HR** | **P** |  |  |
| p6146 | 1.8901 | 6.33E-24 | 1.8869 | 1.41E-28 | 2.7938 | 8.41E-60 | 1.6645 | 8.08E-19 | 2.3783 | 6.07E-08 | 2.1575 | 5.47E-34 | 1.6302 | 2.51E-14 | 1.9311 | 1.89E-54 | 2.0025 | 0.01683 | Social | Disability |
| p680 | 1.7291 | 2.44E-19 | 1.5712 | 2.64E-14 | 1.2717 | 0.009328 | 1.5809 | 6.09E-15 | 1.9237 | 0.000366 | 1.8912 | 1.01E-24 | 1.4713 | 1.73E-09 | 1.7126 | 1.56E-37 | 1.074 | 1 | Social | Rent accommodation versus own |
| p1100 | 0.6554 | 4.38E-20 | 0.7578 | 4.14E-23 | 0.6418 | 1.46E-42 | 0.741 | 1.70E-19 | 0.7009 | 0.002387 | 0.77 | 1.09E-11 | 0.7346 | 4.26E-20 | 0.7559 | 7.00E-33 | 0.6073 | 0.265982 | Lifestyle | Frequent motorway speeding |
| p1080 | 0.8491 | 1.90E-17 | 0.8529 | 1.15E-18 | 0.7856 | 3.63E-33 | 0.8585 | 2.85E-19 | 0.8821 | 1 | 0.8502 | 2.53E-14 | 0.8505 | 1.80E-19 | 0.8618 | 8.97E-30 | 0.8624 | 1 | Lifestyle | Time spent using computer |
| p728 | 0.7905 | 9.21E-16 | 0.8179 | 4.26E-14 | 0.7446 | 6.15E-30 | 0.792 | 2.03E-17 | 0.7058 | 4.71E-05 | 0.7722 | 9.17E-17 | 0.8359 | 2.11E-10 | 0.8025 | 1.03E-29 | 0.774 | 0.428297 | Social | Number of vehicles in household |
| p738 | 0.836 | 2.89E-15 | 0.8711 | 8.78E-13 | 0.6938 | 7.77E-74 | 0.8918 | 1.03E-07 | 0.8524 | 0.217592 | 0.86 | 2.84E-10 | 0.8794 | 6.39E-10 | 0.8715 | 2.63E-21 | 0.9049 | 1 | Social | Total household income |
| p6154_6 | 1.3519 | 0.009008 | 2.1773 | 5.86E-17 | 2.0779 | 2.74E-15 | 1.5451 | 8.16E-07 | 1.6009 | 1 | 1.8491 | 1.75E-10 | 1.6587 | 4.88E-09 | 1.5981 | 2.92E-13 | 2.0727 | 0.095967 | Chemical | Usually takes Laxatives |
| p2492 | 1.3496 | 2.46E-09 | 1.3389 | 3.40E-10 | 2.1785 | 1.07E-60 | 1.2508 | 3.14E-06 | 1.7456 | 0.000288 | 1.4361 | 3.41E-11 | 1.2693 | 1.03E-06 | 1.3693 | 4.81E-22 | 1.6227 | 0.311764 | Chemical | Takes other prescription medications |
| p6144_1 | 1.6763 | 2.05E-07 | 1.663 | 4.35E-08 | 1.8972 | 5.55E-10 | 1.6514 | 3.24E-09 | 2.2113 | 0.013814 | 1.7263 | 6.02E-07 | 1.681 | 8.73E-09 | 1.7281 | 1.26E-17 | 1.2205 | 1 | Lifestyle | Never eat eggs |
| p1090 | 0.8729 | 4.75E-13 | 0.9111 | 0.000457 | 0.8193 | 1.85E-23 | 0.886 | 2.08E-10 | 0.778 | 7.65E-06 | 0.8633 | 8.32E-11 | 0.9 | 5.25E-07 | 0.8748 | 5.65E-22 | 0.8977 | 1 | Lifestyle | Time spent driving |
| p6162_1 | 0.7569 | 1.11E-08 | 0.7772 | 5.79E-07 | 0.6807 | 1.83E-14 | 0.7618 | 8.09E-10 | 0.5979 | 0.000255 | 0.7359 | 1.97E-08 | 0.7801 | 3.55E-07 | 0.7274 | 2.95E-23 | 0.8317 | 1 | Lifestyle | Uses car/motor vehicle as transport |
| p6142 | 1.6745 | 3.78E-06 | 1.6427 | 1.03E-08 | 1.3145 | 0.002161 | 1.4249 | 1 | 1.5852 | 1 | 1.9542 | 2.54E-13 | 1.5452 | 7.25E-05 | 1.7268 | 8.10E-18 | 1.5652 | 1 | Social | Unemployed |
| p137 | 1.2219 | 3.20E-10 | 1.1783 | 5.99E-09 | 1.559 | 2.11E-51 | 1.1466 | 9.66E-06 | 1.201 | 1 | 1.2251 | 4.74E-09 | 1.1451 | 9.46E-06 | 1.214 | 6.58E-22 | 1.2994 | 1 | Chemical | Number of medications taken |
| p6164_2 | 0.7307 | 5.51E-12 | 0.7829 | 1.57E-07 | 0.6605 | 3.53E-18 | 0.7538 | 1.23E-11 | 0.728 | 0.565456 | 0.7228 | 4.56E-10 | 0.7482 | 2.59E-11 | 0.7945 | 3.74E-13 | 1.1556 | 1 | Lifestyle | Engages in other exercises |
| p6141 | 0.8219 | 0.000236 | 0.7659 | 5.41E-08 | 0.8203 | 0.001923 | 0.8005 | 1.51E-06 | 0.7268 | 0.516604 | 0.6995 | 8.34E-13 | 0.8382 | 0.002399 | 0.8014 | 2.90E-11 | 0.669 | 0.751614 | Social | Lives with partner |
| p6138 | 1.314 | 1.30E-08 | 1.2882 | 1.82E-08 | 1.3745 | 3.45E-11 | 1.3026 | 7.51E-10 | 1.1383 | 1 | 1.3438 | 2.28E-08 | 1.282 | 3.87E-08 | 1.2987 | 6.53E-17 | 1.0884 | 1 | Social | Lower education |
| p6164_5 | 0.8245 | 0.001608 | 0.7932 | 1.69E-07 | 0.881 | 0.869964 | 0.8201 | 1.86E-05 | 0.6685 | 0.031659 | 0.7851 | 2.52E-05 | 0.8628 | 0.027147 | 0.819 | 9.73E-10 | 0.7056 | 1 | Lifestyle | Heavy DIY |
| p1269 | 1.3619 | 0.000256 | 1.325 | 0.000409 | 1.3534 | 0.000268 | 1.2596 | 0.021759 | 1.7956 | 0.007646 | 1.4141 | 5.30E-05 | 1.2684 | 0.021726 | 1.4152 | 5.77E-13 | 1.4484 | 1 | Chemical | Exposure to tobacco smoke at home |
| p6144_3 | 1.2677 | 1 | 1.8835 | 6.26E-12 | 1.6292 | 0.000194 | 1.6448 | 1.16E-08 | 1.2436 | 1 | 1.5492 | 0.001411 | 1.6331 | 2.48E-07 | 1.5621 | 3.11E-10 | 1.4593 | 1 | Lifestyle | Never eat wheat products |
| p6145_6 | 1.4188 | 5.06E-05 | 1.3246 | 0.001923 | 0.8605 | 1 | 1.1813 | 1 | 1.5571 | 0.82215 | 1.583 | 4.31E-09 | 1.2251 | 0.638504 | 1.3931 | 1.37E-09 | 1.5781 | 1 | Social | Financial difficulties |
| p709 | 0.9098 | 0.012622 | 0.9094 | 0.007346 | 0.7419 | 7.17E-48 | 0.8996 | 0.000886 | 0.8104 | 0.085369 | 0.8681 | 9.33E-06 | 0.935 | 0.630949 | 0.9052 | 1.34E-06 | 0.8664 | 1 | Social | Number in household |
| p1110 | 0.9368 | 0.000252 | 0.9493 | 0.002378 | 0.8363 | 9.31E-33 | 0.9314 | 6.61E-08 | 0.9762 | 1 | 0.9202 | 8.11E-07 | 0.9543 | 0.02388 | 0.9436 | 3.30E-08 | 0.9452 | 1 | Lifestyle | Length of mobile phone use |
| p2020 | 1.194 | 0.023352 | 1.3322 | 1.57E-06 | 1.1648 | 0.404165 | 1.2316 | 0.001366 | 1.7345 | 0.000322 | 1.3588 | 1.23E-06 | 1.1927 | 0.036997 | 1.3018 | 4.22E-12 | 1.2982 | 1 | Social | Loneliness, isolation |
| p1200 | 0.8417 | 4.98E-07 | 0.9099 | 0.039986 | 0.9471 | 1 | 0.9011 | 0.006697 | 0.9377 | 1 | 0.9191 | 1 | 0.871 | 2.99E-05 | 0.886 | 1.02E-07 | 0.8962 | 1 | Lifestyle | Experiences insomnia frequently |
| p1239 | 1.269 | 2.15E-09 | 1.0712 | 1 | 1.074 | 1 | 1.0644 | 1 | 1.2622 | 0.62803 | 1.2635 | 5.09E-09 | 1.0529 | 1 | 1.2026 | 2.94E-12 | 1.1703 | 1 | Lifestyle | Current tobacco smoking frequency |
| p6164_4 | 0.8257 | 0.000172 | 0.8374 | 0.000342 | 0.8746 | 0.267272 | 0.8687 | 0.012605 | 0.7454 | 0.623793 | 0.8701 | 0.223979 | 0.872 | 0.037758 | 0.8196 | 7.25E-11 | 0.9026 | 1 | Lifestyle | Light DIY |
| p924 | 0.8883 | 0.089797 | 0.8319 | 2.22E-06 | 0.6916 | 9.17E-23 | 0.8603 | 0.000195 | 0.7299 | 0.056645 | 0.8079 | 1.86E-06 | 0.9377 | 1 | 0.8831 | 3.69E-05 | 0.7865 | 1 | Lifestyle | Usually walks quickly |
| p6160_1 | 0.7658 | 6.70E-06 | 0.8431 | 0.032567 | 0.7399 | 7.92E-07 | 0.8259 | 0.001963 | 0.8504 | 1 | 0.768 | 0.000187 | 0.7732 | 3.57E-06 | 0.8338 | 9.79E-06 | 1.1825 | 1 | Lifestyle | Attends a sports club or gym frequently |
| p1488 | 0.9765 | 1 | 0.9147 | 0.000226 | 0.9546 | 1 | 0.9412 | 0.135942 | 0.8802 | 1 | 0.9131 | 0.002007 | 0.9211 | 0.000941 | 0.9224 | 2.34E-07 | 0.9188 | 1 | Lifestyle | Tea intake |
| p2159 | 1.4227 | 3.04E-05 | 1.222 | 0.51759 | 1.1656 | 1 | 1.365 | 4.82E-05 | 1.3012 | 1 | 1.3049 | 0.076175 | 1.248 | 0.183736 | 1.2758 | 0.000237 | 1.4405 | 1 | Lifestyle | Had same-sex intercourse |
| p6144_4 | 1.3832 | 3.52E-12 | 1.1048 | 1 | 1.7021 | 3.34E-27 | 1.1647 | 0.017478 | 1.386 | 0.491333 | 1.2227 | 0.005063 | 1.1793 | 0.012608 | 1.2127 | 5.54E-08 | 1.0699 | 1 | Lifestyle | Never eat sugar foods |
| p2277 | 1.0495 | 1 | 1.1374 | 3.46E-08 | 0.9641 | 1 | 1.1032 | 0.000528 | 1.1349 | 1 | 1.1095 | 0.00133 | 1.0747 | 0.091408 | 1.1038 | 9.66E-09 | 0.9961 | 1 | Physical | Uses solarium/sunlamp frequently |
| p1279 | 1.2037 | 0.003854 | 1.1587 | 0.03947 | 1.2247 | 0.000936 | 1.1324 | 0.282845 | 1.4581 | 0.052113 | 1.3086 | 7.53E-07 | 1.0583 | 1 | 1.2154 | 1.40E-08 | 0.9765 | 1 | Chemical | Exposure to tobacco smoke outside home |
| p2060 | 1.16 | 0.000276 | 1.1525 | 0.0002 | 0.9938 | 1 | 1.1692 | 1.90E-05 | 1.2616 | 0.23998 | 1.1069 | 0.506353 | 1.173 | 8.81E-06 | 1.1647 | 1.61E-09 | 1.0275 | 1 | Social | Feels unenthusiasm/disinterest frequently |
| p20116_2 | 1.5838 | 1.78E-09 | 1.1166 | 1 | 1.2374 | 0.173419 | 1.1356 | 1 | 1.5417 | 1 | 1.5293 | 2.15E-07 | 1.1069 | 1 | 1.382 | 1.27E-09 | 1.271 | 1 | Lifestyle | Current smoker |
| p1508_2 | 0.8809 | 1 | 0.7817 | 0.003086 | 0.7335 | 0.000134 | 0.7982 | 0.004839 | 0.7297 | 1 | 0.8646 | 1 | 0.8385 | 0.288014 | 0.8101 | 6.47E-05 | 0.7533 | 1 | Lifestyle | Ground coffee versus decaffeinated |
| p900_1 | 1.1081 | 1 | 1.2051 | 0.00014 | 1.2393 | 0.000109 | 1.1091 | 0.859792 | 1.3313 | 0.925131 | 1.1768 | 0.042424 | 1.146 | 0.061867 | 1.1678 | 6.82E-06 | 0.9487 | 1 | Lifestyle | Improper consumption of alcohol |
| p24006 | 1.1077 | 1.95E-05 | 1.0848 | 0.003131 | 1.0634 | 0.583967 | 1.0902 | 0.000426 | 1.1733 | 0.203448 | 1.1194 | 2.78E-05 | 1.0915 | 0.00062 | 1.0793 | 6.59E-06 | 1.1623 | 1 | Chemical | PM2.5 pollution |
| p670_1 | 1.3885 | 1.18E-05 | 1.3266 | 0.000231 | 1.1143 | 1 | 1.3446 | 2.53E-05 | 1.1397 | 1 | 1.4911 | 5.50E-08 | 1.2578 | 0.021866 | 1.3162 | 7.99E-08 | 1.5792 | 0.709679 | Social | Lives in a flat versus house |
| p2139 | 1.1071 | 1 | 1.197 | 0.00037 | 0.8982 | 1 | 1.1553 | 0.018738 | 1.2516 | 1 | 1.2035 | 0.004787 | 1.1457 | 0.076909 | 1.1936 | 8.85E-08 | 1.039 | 1 | Lifestyle | First sexual intercourse (< age 18) |
| p6144_2 | 1.3088 | 0.984454 | 1.4198 | 0.040212 | 1.7252 | 3.56E-05 | 1.4299 | 0.003798 | 1.5528 | 1 | 1.3295 | 1 | 1.5249 | 0.000108 | 1.4825 | 9.85E-07 | 1.7481 | 1 | Lifestyle | Never eat dairy products |
| p2400_1 | 1.1123 | 5.67E-05 | 1.0897 | 0.001942 | 1.0263 | 1 | 1.1061 | 1.55E-05 | 1.0953 | 1 | 1.1077 | 0.00139 | 1.0879 | 0.003173 | 1.0776 | 4.50E-05 | 1.1205 | 1 | Chemical | Nitrogen dioxide/oxides air pollution |
| p2267 | 0.9065 | 0.000605 | 0.936 | 0.194776 | 0.8612 | 2.03E-08 | 0.9453 | 0.603045 | 0.8654 | 1 | 0.9062 | 0.00395 | 0.9182 | 0.004293 | 0.9183 | 2.17E-06 | 0.9743 | 1 | Physical | Uses sun or UV protection frequently |
| p2050 | 1.1201 | 0.040797 | 1.189 | 2.73E-06 | 1.0047 | 1 | 1.1684 | 5.74E-05 | 1.2336 | 0.935743 | 1.137 | 0.030072 | 1.1687 | 5.23E-05 | 1.1871 | 1.97E-12 | 1.0998 | 1 | Social | Feels depressed mood frequently |
| p2000 | 0.7989 | 1.80E-06 | 0.9058 | 1 | 0.7705 | 1.75E-07 | 0.8671 | 0.01771 | 0.8286 | 1 | 0.8562 | 0.077222 | 0.8835 | 0.204561 | 0.8762 | 0.000514 | 1.0147 | 1 | Social | Worry too long after embarrassment |
| p1920 | 1.1423 | 0.161087 | 1.1796 | 0.002716 | 0.9718 | 1 | 1.1537 | 0.0148 | 1.3549 | 0.493512 | 1.1486 | 0.312272 | 1.1528 | 0.031815 | 1.18 | 7.29E-07 | 1.3464 | 1 | Social | Mood swings |
| p2070 | 1.1029 | 0.342351 | 1.1481 | 0.000799 | 0.9996 | 1 | 1.1192 | 0.045312 | 1.1167 | 1 | 1.1182 | 0.189192 | 1.1033 | 0.30579 | 1.1337 | 5.65E-06 | 1.1854 | 1 | Social | Feels tenseness/restlessness frequently |
| p6154_1 | 1.3007 | 1.16E-05 | 1.1355 | 0.307268 | 1.9976 | 7.74E-41 | 1.1676 | 0.015581 | 1.2978 | 1 | 1.2443 | 0.001573 | 1.1539 | 0.166216 | 1.184 | 2.30E-05 | 1.9079 | 0.0013 | Chemical | Usually takes Aspirin |
| p22039 | 0.9304 | 0.040636 | 0.9618 | 1 | 0.9056 | 0.000434 | 0.9478 | 0.46036 | 0.9372 | 1 | 0.9685 | 1 | 0.9108 | 7.41E-05 | 0.9678 | 1 | 1.0118 | 1 | Lifestyle | MET minutes/week of vigorous activity |
| p6139_3 | 0.6707 | 0.001791 | 0.7939 | 0.606564 | 0.6291 | 0.000116 | 0.8104 | 1 | 0.6616 | 1 | 0.7655 | 0.842426 | 0.7467 | 0.06187 | 0.7525 | 0.000308 | 0.5753 | 1 | Chemical | Uses open solid fuel fire in winter |
| p1220 | 1.133 | 0.07972 | 1.1093 | 0.226114 | 1.3015 | 1.15E-10 | 1.1296 | 0.01397 | 1.3741 | 0.018093 | 1.2232 | 5.65E-06 | 1.0232 | 1 | 1.1322 | 6.13E-05 | 1.1057 | 1 | Lifestyle | Daytime sleep frequency |
| p24506 | 0.9241 | 0.003932 | 0.9447 | 0.522939 | 0.9793 | 1 | 0.9366 | 0.038464 | 1.0046 | 1 | 0.9115 | 0.001278 | 0.9487 | 0.99239 | 0.9373 | 0.000731 | 0.943 | 1 | Ecosystems | Natural environment percentage (1000m buffer) |
| p2110 | 0.9676 | 0.282187 | 0.9657 | 0.016255 | 0.9537 | 0.001534 | 0.9639 | 0.005882 | 0.9799 | 1 | 0.9623 | 0.06021 | 0.9593 | 0.001423 | 0.9683 | 0.000585 | 0.9617 | 1 | Social | Able to confide frequently |
| p2010 | 1.1786 | 0.085379 | 1.1186 | 1 | 0.993 | 1 | 1.1472 | 0.384352 | 1.1989 | 1 | 1.1217 | 1 | 1.1272 | 1 | 1.1566 | 0.002628 | 1.3727 | 1 | Social | Suffer from 'nerves' |
| p699 | 0.9613 | 1 | 0.9327 | 0.031322 | 1.2636 | 1.22E-20 | 0.9658 | 1 | 0.9452 | 1 | 0.9154 | 0.004573 | 0.9555 | 1 | 0.9458 | 0.010239 | 1.0157 | 1 | Social | Length of residence at current address |
| p6145_1 | 1.109 | 1 | 1.2272 | 0.024624 | 1.2682 | 0.018002 | 1.147 | 1 | 1.2192 | 1 | 1.3088 | 0.002737 | 1.0366 | 1 | 1.1868 | 0.010054 | 1.1601 | 1 | Social | Own serious illness or injury |
| p943 | 0.9641 | 1 | 0.9768 | 1 | 0.9621 | 1 | 0.9706 | 1 | 0.9393 | 1 | 0.9693 | 1 | 0.9743 | 1 | 0.9714 | 1 | 0.8945 | 1 | Lifestyle | Climbes stair frequently |
| p6155_2 | 1.2808 | 0.268878 | 1.3964 | 0.020471 | 1.3767 | 0.057412 | 1.1497 | 1 | 1.6058 | 1 | 1.3637 | 0.114701 | 1.2421 | 1 | 1.3099 | 0.00119 | 1.3703 | 1 | Chemical | Vitamin B supplements |
| p6160_4 | 0.6821 | 1.94E-05 | 0.8596 | 1 | 1.0195 | 1 | 0.6848 | 7.14E-05 | 0.7962 | 1 | 0.8618 | 1 | 0.809 | 0.723788 | 0.8128 | 0.036311 | 1.2966 | 1 | Lifestyle | Attends a adult education class frequently |
| p670_2 | 1.7302 | 0.030217 | 1.6426 | 0.092011 | 2.621 | 9.51E-07 | 1.6282 | 0.026649 | 1.7823 | 1 | 1.6385 | 0.663962 | 1.756 | 0.007442 | 1.6351 | 0.001438 | 2.3383 | 1 | Social | Lives in mobile structure versus house |
| p1548 | 1.0114 | 1 | 1.0975 | 0.402365 | 1.0717 | 1 | 1.0838 | 1 | 1.0175 | 1 | 1.0487 | 1 | 1.096 | 0.634982 | 1.1156 | 0.000391 | 1.0438 | 1 | Lifestyle | Diet varies frequently |
| p900_7 | 0.8968 | 1 | 0.9178 | 1 | 1.0142 | 1 | 0.9179 | 1 | 0.8194 | 1 | 0.8279 | 0.002225 | 0.9628 | 1 | 0.8918 | 0.007156 | 1.0005 | 1 | Lifestyle | Wholegrain eaten above recommendation |
| p2400_2 | 1.0722 | 0.189146 | 1.054 | 1 | 1.0058 | 1 | 1.065 | 0.191549 | 1.0713 | 1 | 1.1049 | 0.003542 | 1.0378 | 1 | 1.0518 | 0.118277 | 1.0382 | 1 | Chemical | PM10 pollution |
| p6164_1 | 0.8931 | 1 | 0.9085 | 1 | 0.9275 | 1 | 0.8517 | 0.004297 | 0.7442 | 0.769991 | 0.8908 | 1 | 0.9064 | 1 | 0.8836 | 0.005811 | 0.8643 | 1 | Lifestyle | Walks for pleasure |
| p1990 | 1.0823 | 1 | 1.1894 | 0.08887 | 1.0091 | 1 | 1.1632 | 0.19189 | 0.9877 | 1 | 1.1946 | 0.194552 | 1.0807 | 1 | 1.1511 | 0.012503 | 1.1393 | 1 | Social | Tense/'highly strung' |
| p22038 | 0.9358 | 0.105068 | 0.9594 | 1 | 1.0024 | 1 | 0.9594 | 1 | 0.9134 | 1 | 0.9359 | 0.179101 | 0.9599 | 1 | 0.9551 | 0.100141 | 0.9965 | 1 | Lifestyle | MET minutes/week of moderate activity |
| p1980 | 1.0616 | 1 | 1.1546 | 0.022279 | 1.104 | 1 | 1.0733 | 1 | 1.1613 | 1 | 1.0511 | 1 | 1.1035 | 1 | 1.1129 | 0.034813 | 1.0043 | 1 | Social | Worrier/anxious feelings |
| p1428_2 | 1.3215 | 1 | 1.4517 | 1 | 1.9251 | 0.239682 | 1.5416 | 1 | 0.8033 | 1 | 2.321 | 0.004833 | 1.1704 | 1 | 1.6689 | 0.087785 | 2.8188 | 1 | Lifestyle | Flora Pro-Active spread versus rare/non-spread |
| p2080 | 1.0992 | 0.016959 | 1.0512 | 1 | 0.9468 | 1 | 1.1088 | 0.001298 | 1.1861 | 0.787893 | 1.1163 | 0.005288 | 1.0645 | 1 | 1.092 | 9.75E-05 | 1.0139 | 1 | Social | Feels tiredness/lethargy frequently |
| p1259 | 1.2295 | 0.136825 | 1.1682 | 1 | 1.0283 | 1 | 1.127 | 1 | 1.3651 | 1 | 1.2069 | 0.787865 | 1.1511 | 1 | 1.1809 | 0.023096 | 1.9277 | 0.019013 | Chemical | Had smoking/smokers in household |
| p900_3 | 1.1756 | 1 | 1.4945 | 0.001323 | 1.3453 | 0.916717 | 1.3662 | 0.118359 | 0.8933 | 1 | 1.3097 | 1 | 1.5053 | 0.001133 | 1.2911 | 0.101332 | 1.1593 | 1 | Lifestyle | Vegetable eaten above recommendation |
| p900_2 | 1.0883 | 1 | 1.1371 | 0.382272 | 1.0728 | 1 | 1.1308 | 0.352918 | 1.1683 | 1 | 1.2109 | 0.007542 | 1.1688 | 0.023382 | 1.1282 | 0.013722 | 0.9504 | 1 | Lifestyle | Improper sleep duration <7/>9 hours |
| p1190 | 1.091 | 1 | 1.0586 | 1 | 1.3078 | 1.88E-13 | 1.0454 | 1 | 1.3996 | 0.002121 | 1.1278 | 0.069381 | 0.9783 | 1 | 1.0748 | 0.196687 | 1.2513 | 1 | Lifestyle | Naps frequently |
| p1408 | 0.9735 | 1 | 0.9617 | 1 | 0.9731 | 1 | 0.9283 | 0.001324 | 0.9289 | 1 | 0.962 | 1 | 0.9564 | 1 | 0.9653 | 1 | 1.0171 | 1 | Lifestyle | Cheese intake |
| p1359 | 0.9478 | 1 | 0.9854 | 1 | 0.9222 | 0.062738 | 0.9839 | 1 | 1.0644 | 1 | 0.9574 | 1 | 0.9491 | 1 | 0.9582 | 1 | 1.0289 | 1 | Lifestyle | Poultry intake |
| p6162_3 | 1.0521 | 1 | 1.0473 | 1 | 1.4953 | 4.53E-18 | 1.0615 | 1 | 1.193 | 1 | 1.1844 | 0.022089 | 1.019 | 1 | 1.1043 | 0.085274 | 0.9273 | 1 | Lifestyle | Uses public transport |
| p24012 | 1.0446 | 1 | 1.039 | 1 | 1.0098 | 1 | 1.0473 | 1 | 1.0208 | 1 | 1.0401 | 1 | 1.0624 | 0.172435 | 1.0257 | 1 | 1.1008 | 1 | Physical | Inverse distance to major road |
| p24508 | 1.0255 | 1 | 1.046 | 1 | 1.0258 | 1 | 1.0431 | 1 | 1.0547 | 1 | 1.0351 | 1 | 1.053 | 1 | 1.0293 | 1 | 1.1119 | 1 | Ecosystems | Distance to coast |
| p6179_5 | 1.2136 | 1 | 1.4416 | 0.12572 | 0.9564 | 1 | 1.2525 | 1 | 2.2763 | 0.026656 | 1.0941 | 1 | 1.1872 | 1 | 1.2589 | 0.825217 | 1.3347 | 1 | Chemical | Iron supplements |
| p1767 | 1.4032 | 1 | 1.093 | 1 | 1.7987 | 0.000993 | 1.0375 | 1 | 1.8127 | 1 | 1.27 | 1 | 1.2594 | 1 | 1.4109 | 0.072344 | 1.4458 | 1 | Social | Adopted as a child |
| p1757 | 1.0841 | 1 | 1.1045 | 1 | 0.9946 | 1 | 1.0741 | 1 | 1.0595 | 1 | 1.1212 | 1 | 1.0567 | 1 | 1.0747 | 1 | 1.2015 | 1 | Physical | Degree of facial ageing |
| p6145_2 | 0.8636 | 1 | 0.816 | 1 | 0.6881 | 6.26E-05 | 0.8407 | 1 | 0.9508 | 1 | 0.8119 | 1 | 0.8459 | 1 | 0.8017 | 0.003128 | 0.8108 | 1 | Social | Relativeâ€™s serious illness or injury |
| p22037 | 0.9589 | 1 | 0.9427 | 0.058572 | 0.9743 | 1 | 0.9611 | 1 | 0.8848 | 0.389018 | 0.9423 | 0.472042 | 0.9659 | 1 | 0.9566 | 0.186637 | 0.9616 | 1 | Lifestyle | MET minutes/week of walking |
| p1970 | 1.0474 | 1 | 1.1685 | 0.113502 | 1.0473 | 1 | 1.0405 | 1 | 1.1901 | 1 | 1.1382 | 1 | 1.0242 | 1 | 1.0965 | 0.781285 | 1.322 | 1 | Social | Nervous feelings |
| p22034 | 0.939 | 0.274348 | 0.9595 | 1 | 0.9807 | 1 | 0.9617 | 1 | 0.9269 | 1 | 0.9376 | 0.052805 | 0.9514 | 0.679616 | 0.9556 | 0.047455 | 0.8848 | 1 | Lifestyle | Summed minutes activity |
| p6145_5 | 1.3583 | 1 | 1.3626 | 1 | 0.8963 | 1 | 1.1655 | 1 | 1.7132 | 1 | 1.4886 | 0.722985 | 1.1977 | 1 | 1.3785 | 0.140709 | 1.4972 | 1 | Social | Marital separation/divorce |
| p6160_5 | 0.8765 | 0.433577 | 0.8889 | 1 | 1.1575 | 0.864308 | 0.8291 | 0.001318 | 1.0379 | 1 | 0.9042 | 1 | 0.8627 | 0.14463 | 0.8834 | 0.031153 | 1.1712 | 1 | Lifestyle | Attends other group activities frequently |
| p24007 | 1.0631 | 0.408299 | 1.0343 | 1 | 0.9881 | 1 | 1.0652 | 0.095581 | 0.9828 | 1 | 1.05 | 1 | 1.0551 | 0.916734 | 1.0297 | 1 | 0.9946 | 1 | Chemical | PM2.5 absorbance pollution |
| p22033 | 0.9371 | 0.073103 | 0.9659 | 1 | 0.9907 | 1 | 0.9621 | 1 | 0.9112 | 1 | 0.9438 | 0.301759 | 0.9509 | 0.831352 | 0.9669 | 1 | 0.8729 | 0.653288 | Lifestyle | Summed days activity |
| p1518 | 0.9084 | 0.688877 | 0.9273 | 1 | 0.9383 | 1 | 0.92 | 1 | 0.8834 | 1 | 0.8958 | 0.496134 | 0.9678 | 1 | 0.9064 | 0.006603 | 0.9043 | 1 | Lifestyle | Hot drink temperature |
| p6154_4 | 1.365 | 0.604446 | 1.3244 | 1 | 1.303 | 1 | 1.1539 | 1 | 1.6559 | 1 | 1.2878 | 1 | 1.2291 | 1 | 1.3051 | 0.147202 | 1.718 | 1 | Chemical | Usually takes Ranitidine |
| p1478 | 1.0334 | 1 | 1.0357 | 1 | 1.047 | 1 | 0.995 | 1 | 1.0959 | 1 | 1.0825 | 0.134236 | 0.9969 | 1 | 1.0473 | 0.528702 | 1.1005 | 1 | Lifestyle | Adds salt to food frequently |
| p6162_4 | 0.7642 | 1 | 0.8754 | 1 | 0.6574 | 0.006006 | 0.7674 | 0.765171 | 0.8302 | 1 | 0.7285 | 1 | 0.9598 | 1 | 0.9047 | 1 | 0.8973 | 1 | Lifestyle | Uses cycle as transport |
| p6145_4 | 1.3134 | 1 | 1.2218 | 1 | 1.9235 | 0.000173 | 1.2554 | 1 | 1.3403 | 1 | 1.351 | 1 | 1.0375 | 1 | 1.2081 | 1 | 1.7809 | 1 | Social | Death of a spouse or partner |
| p6154_3 | 1.1683 | 0.053009 | 1.0417 | 1 | 1.0063 | 1 | 1.0744 | 1 | 1.0152 | 1 | 1.1105 | 1 | 1.0985 | 1 | 1.0849 | 1 | 1.2184 | 1 | Chemical | Usually takes Paracetamol |
| p1960 | 1.1206 | 0.964796 | 1.0539 | 1 | 0.8613 | 0.120456 | 1.0741 | 1 | 1.2187 | 1 | 1.0774 | 1 | 1.0782 | 1 | 1.1118 | 0.049384 | 1.385 | 1 | Social | Fed-up feelings |
| p1418_4 | 0.9993 | 1 | 0.5461 | 0.072683 | 0.7617 | 1 | 0.7843 | 1 | 0.4452 | 1 | 0.5688 | 0.398116 | 0.9856 | 1 | 0.8281 | 1 | 3.118 | 1 | Lifestyle | Soya milk versus rare/non-milk |
| p1130 | 1.0286 | 1 | 0.9994 | 1 | 0.9412 | 1 | 1.0321 | 1 | 0.9948 | 1 | 1.0507 | 1 | 1.0189 | 1 | 1.0288 | 1 | 1.0275 | 1 | Lifestyle | Uses hands-free or speakerphone frequently |
| p6139_2 | 0.9472 | 1 | 0.9163 | 1 | 1.1305 | 0.694358 | 0.9103 | 1 | 1.0408 | 1 | 0.8979 | 1 | 0.9851 | 1 | 0.9699 | 1 | 0.8874 | 1 | Chemical | Uses gas fire in winter |
| p1140_3 | 1.295 | 1 | 0.9013 | 1 | 1.3091 | 1 | 1.0695 | 1 | 1.209 | 1 | 1.06 | 1 | 1.2672 | 1 | 1.1504 | 1 | 0.7805 | 1 | Lifestyle | Didn't use a mobile phone versus no difference |
| p22040 | 0.9683 | 1 | 0.9595 | 1 | 0.9721 | 1 | 0.9554 | 0.85206 | 0.9343 | 1 | 0.943 | 0.188646 | 0.9716 | 1 | 0.969 | 1 | 0.9044 | 1 | Lifestyle | MET minutes/week of all activity |
| p6164_3 | 0.9266 | 1 | 0.8522 | 1 | 0.4837 | 1.43E-11 | 0.9113 | 1 | 1.3918 | 1 | 0.8749 | 1 | 0.8514 | 1 | 0.8818 | 1 | 1.2981 | 1 | Lifestyle | Engages in strenuous sports |
| p1210 | 1.0259 | 1 | 0.8723 | 0.046838 | 0.932 | 1 | 0.8922 | 0.469662 | 0.9808 | 1 | 0.9244 | 1 | 0.9347 | 1 | 0.93 | 1 | 0.9051 | 1 | Lifestyle | Snoring |
| p6162_2 | 0.9319 | 1 | 0.9167 | 1 | 1.0566 | 1 | 0.909 | 1 | 0.7472 | 0.786876 | 0.9161 | 1 | 0.95 | 1 | 0.9517 | 1 | 0.8708 | 1 | Lifestyle | Walks as transport |
| p1737 | 0.9558 | 1 | 0.9996 | 1 | 0.85 | 4.53E-12 | 0.948 | 0.994553 | 1.0119 | 1 | 0.9848 | 1 | 0.9556 | 1 | 0.9926 | 1 | 0.9147 | 1 | Physical | Childhood sunburn occurred frequently |
| p864 | 0.9519 | 1 | 0.9601 | 1 | 0.9771 | 1 | 0.9713 | 1 | 0.8912 | 0.131464 | 0.9468 | 0.430702 | 0.9856 | 1 | 0.9705 | 1 | 0.895 | 1 | Lifestyle | Days/week walked 10+ minutes |
| p20116_1 | 1.0746 | 1 | 1.0266 | 1 | 1.2458 | 0.000276 | 1.0592 | 1 | 1.1416 | 1 | 1.0555 | 1 | 1.0687 | 1 | 1.0704 | 1 | 1.0771 | 1 | Lifestyle | Previous smoker |
| p1538 | 1.0891 | 1 | 1.1277 | 0.304992 | 1.1148 | 1 | 1.0192 | 1 | 1.4175 | 0.095295 | 1.0166 | 1 | 1.0468 | 1 | 1.0529 | 1 | 1.1179 | 1 | Lifestyle | Major dietary changes |
| p100_1 | 0.9463 | 1 | 1.013 | 1 | 1.1011 | 1 | 1.0302 | 1 | 0.822 | 1 | 0.8954 | 1 | 1.0541 | 1 | 0.9648 | 1 | 1.0572 | 1 | Lifestyle | Healthy diet |
| p6155_1 | 1.1023 | 1 | 1.2874 | 1 | 1.3545 | 1 | 1.0411 | 1 | 1.4675 | 1 | 1.2078 | 1 | 1.0079 | 1 | 1.0847 | 1 | 0.8802 | 1 | Chemical | Vitamin A supplements |
| p6155_6 | 1.345 | 0.376893 | 1.3902 | 0.67561 | 1.5533 | 0.010959 | 1.2275 | 1 | 0.6395 | 1 | 1.2865 | 1 | 1.3756 | 0.253308 | 1.4123 | 0.000449 | 1.4441 | 1 | Chemical | Folic acid or Folate (Vit B9) supplements |
| p900_9 | 0.9905 | 1 | 0.961 | 1 | 1.0171 | 1 | 0.9603 | 1 | 0.9653 | 1 | 0.9104 | 1 | 1.0425 | 1 | 0.9612 | 1 | 0.7646 | 1 | Lifestyle | Processed meat intake reduced |
| p24500 | 0.9493 | 1 | 0.95 | 1 | 1.0058 | 1 | 0.9653 | 1 | 0.9613 | 1 | 0.9429 | 1 | 0.9692 | 1 | 0.9676 | 1 | 0.8983 | 1 | Ecosystems | Greenspace percentage (1000 m buffer) |
| p900_6 | 0.7795 | 1 | 0.693 | 1 | 0.7478 | 1 | 0.7751 | 1 | 0.7594 | 1 | 0.7632 | 1 | 0.7772 | 1 | 0.783 | 1 | 0.2629 | 1 | Lifestyle | Whole bread/refined cereals intake reduced |
| p6179_2 | 0.8737 | 0.475897 | 0.9469 | 1 | 1.2882 | 0.000194 | 0.9229 | 1 | 0.7617 | 1 | 0.8942 | 1 | 0.9759 | 1 | 0.9235 | 1 | 0.8538 | 1 | Chemical | Glucosamine supplements |
| p1528 | 1.0082 | 1 | 1.0688 | 0.038638 | 0.9725 | 1 | 1.048 | 1 | 0.9425 | 1 | 1.0242 | 1 | 1.0631 | 0.389506 | 1.0422 | 0.589126 | 1.05 | 1 | Lifestyle | Water intake |
| p1150_2 | 0.9331 | 1 | 1.2289 | 1 | 1.1958 | 1 | 1.187 | 1 | 0.8532 | 1 | 1.0517 | 1 | 1.1769 | 1 | 1.0616 | 1 | 1.4961 | 1 | Lifestyle | Prefers right side of head for mobile phone use |
| p1687_2 | 1.0315 | 1 | 1.0621 | 1 | 0.9501 | 1 | 1.0057 | 1 | 1.3257 | 1 | 1.136 | 1 | 1.0029 | 1 | 1.0899 | 1 | 0.8517 | 1 | Social | Relatively plumper at 10 years old |
| p1508_3 | 1.0237 | 1 | 1.2014 | 1 | 1.1692 | 1 | 1.0316 | 1 | 1.1374 | 1 | 1.4254 | 1 | 1.0295 | 1 | 1.1953 | 1 | 0.8198 | 1 | Lifestyle | Other coffee types versus decaffeinated |
| p2030 | 0.8345 | 0.004936 | 1.0552 | 1 | 0.8516 | 0.125956 | 0.9467 | 1 | 1.0094 | 1 | 0.9871 | 1 | 0.9353 | 1 | 0.9827 | 1 | 0.841 | 1 | Social | Guilty feelings |
| p1140_2 | 0.8873 | 1 | 0.9785 | 1 | 0.7037 | 5.66E-11 | 1.0127 | 1 | 0.8211 | 1 | 0.9984 | 1 | 0.9909 | 1 | 0.9449 | 1 | 0.8572 | 1 | Lifestyle | More frequent mobile phone use versus no difference |
| p2237 | 0.9115 | 1 | 0.9676 | 1 | 0.8357 | 0.00682 | 0.9323 | 1 | 0.953 | 1 | 0.8785 | 0.988057 | 0.9459 | 1 | 0.9676 | 1 | 0.9839 | 1 | Lifestyle | Plays computer games frequently |
| p6139_1 | 0.9879 | 1 | 0.9321 | 1 | 0.8875 | 1 | 0.9301 | 1 | 0.9691 | 1 | 0.9006 | 1 | 0.9625 | 1 | 0.9546 | 1 | 0.7431 | 1 | Chemical | Uses gas hob or cooker |
| p904 | 0.9654 | 1 | 0.9908 | 1 | 0.9361 | 0.32094 | 0.9763 | 1 | 0.9354 | 1 | 0.9752 | 1 | 0.9641 | 1 | 1.001 | 1 | 1.0563 | 1 | Lifestyle | Days/week of vigorous physical activity |
| p884 | 0.9632 | 1 | 0.9834 | 1 | 1.0049 | 1 | 0.9938 | 1 | 0.9116 | 1 | 0.9771 | 1 | 0.9843 | 1 | 0.9867 | 1 | 1.0273 | 1 | Lifestyle | Days/week moderate physical activity 10+ minutes |
| p24507 | 0.936 | 0.11164 | 0.9823 | 1 | 0.9732 | 1 | 0.9548 | 1 | 1.0339 | 1 | 0.9356 | 0.256913 | 0.9869 | 1 | 0.9704 | 1 | 0.9259 | 1 | Ecosystems | Natural environment percentage (300 m buffer) |
| p20127 | 0.9886 | 1 | 1.0282 | 1 | 0.9374 | 0.38268 | 1.0242 | 1 | 1.0269 | 1 | 1.0034 | 1 | 1.0101 | 1 | 1.0219 | 1 | 1.0898 | 1 | Social | Neuroticism score |
| p874 | 1.0455 | 1 | 1.0175 | 1 | 1.0115 | 1 | 1.029 | 1 | 0.9605 | 1 | 0.9977 | 1 | 1.0418 | 1 | 1.0247 | 1 | 0.9977 | 1 | Lifestyle | Duration of walks |
| p1697_2 | 0.9132 | 1 | 0.9206 | 1 | 0.858 | 0.647736 | 0.9182 | 1 | 1.0545 | 1 | 0.992 | 1 | 0.8926 | 1 | 0.9305 | 1 | 1.2588 | 1 | Social | Relatively taller at 10 years old |
| p1050 | 0.9936 | 1 | 1.0335 | 1 | 1.164 | 5.53E-09 | 0.9722 | 1 | 1.0791 | 1 | 0.9837 | 1 | 1.055 | 1 | 1.0203 | 1 | 0.9112 | 1 | Physical | Time spent outdoors (summer) |
| p1707_1 | 0.8045 | 1 | 1.0138 | 1 | 0.7886 | 1 | 0.8437 | 1 | 1.4419 | 1 | 0.7678 | 1 | 0.9275 | 1 | 0.8408 | 1 | 1.3589 | 1 | Lifestyle | Right-handed versus ambidextrous |
| p6160_2 | 1.1057 | 1 | 1.0493 | 1 | 1.0172 | 1 | 1.0135 | 1 | 0.9715 | 1 | 1.0326 | 1 | 1.1025 | 1 | 1.0369 | 1 | 0.9397 | 1 | Lifestyle | Attends a pub or social club frequently |
| p6179_4 | 0.9247 | 1 | 0.9078 | 1 | 1.0538 | 1 | 0.8374 | 1 | 1.1662 | 1 | 0.8536 | 1 | 0.9601 | 1 | 0.9305 | 1 | 0.66 | 1 | Chemical | Zinc supplements |
| p1180 | 0.9843 | 1 | 0.9966 | 1 | 0.922 | 0.025035 | 0.9791 | 1 | 1.0156 | 1 | 0.9807 | 1 | 0.9876 | 1 | 0.9885 | 1 | 1.0696 | 1 | Lifestyle | More evening-type chronotype |
| p1120 | 0.9731 | 1 | 0.9526 | 1 | 0.8117 | 8.72E-27 | 0.9978 | 1 | 1.0397 | 1 | 0.9876 | 1 | 0.9929 | 1 | 0.9832 | 1 | 0.9423 | 1 | Lifestyle | Weekly mobile phone usage |
| p1787 | 1.0448 | 1 | 0.975 | 1 | 0.9144 | 1 | 1.0769 | 1 | 0.9845 | 1 | 1.0802 | 1 | 1.0543 | 1 | 1.0628 | 1 | 1.2045 | 1 | Chemical | Maternal smoking around birth |
| p1418_3 | 1.0064 | 1 | 0.8114 | 1 | 0.9826 | 1 | 0.9903 | 1 | 1.035 | 1 | 0.8648 | 1 | 0.9115 | 1 | 0.8807 | 1 | 4.2183 | 1 | Lifestyle | Skimmed milk versus rare/non-milk |
| p24011 | 1.0204 | 1 | 1.0033 | 1 | 1.0082 | 1 | 1.0116 | 1 | 1.0212 | 1 | 1.0213 | 1 | 0.9975 | 1 | 1.0204 | 1 | 0.9585 | 1 | Physical | Traffic intensity on nearest major road |
| p24008 | 1.0004 | 1 | 0.9869 | 1 | 1.001 | 1 | 1.0041 | 1 | 1.0292 | 1 | 1.0173 | 1 | 0.9987 | 1 | 1.0059 | 1 | 0.9792 | 1 | Chemical | PM2.5-10 pollution |
| p1418_2 | 1.0051 | 1 | 0.8086 | 1 | 0.8726 | 1 | 0.994 | 1 | 0.9223 | 1 | 0.8723 | 1 | 0.9187 | 1 | 0.8825 | 1 | 3.6422 | 1 | Lifestyle | Semi-skimmed milk versus rare/non-milk |
| p900_4 | 1.034 | 1 | 1.139 | 0.082985 | 1.2973 | 1.23E-07 | 1.0732 | 1 | 0.9305 | 1 | 1.033 | 1 | 1.114 | 0.802034 | 1.0503 | 1 | 1.3179 | 1 | Lifestyle | Fruit eaten above recommendation |
| p900_8 | 0.9849 | 1 | 0.9819 | 1 | 0.9404 | 1 | 0.9874 | 1 | 0.8993 | 1 | 0.966 | 1 | 0.9503 | 1 | 0.9506 | 1 | 0.9368 | 1 | Lifestyle | Red meat intake reduced |
| p1060 | 0.9966 | 1 | 1.0443 | 1 | 1.1587 | 4.87E-07 | 0.9823 | 1 | 0.9741 | 1 | 0.9816 | 1 | 1.0925 | 0.014678 | 1.0293 | 1 | 0.9354 | 1 | Physical | Time spent outdoors (winter) |
| p20160 | 1.086 | 1 | 0.9798 | 1 | 1.1677 | 0.07873 | 1.0114 | 1 | 1.0197 | 1 | 1.0307 | 1 | 1.0391 | 1 | 1.0535 | 1 | 1.15 | 1 | Lifestyle | Ever smoked |
| p1940 | 0.9453 | 1 | 0.9509 | 1 | 0.8048 | 0.000893 | 0.9599 | 1 | 0.9513 | 1 | 0.961 | 1 | 0.9659 | 1 | 0.9665 | 1 | 1.1485 | 1 | Social | Irritability |
| p1070 | 1.0162 | 1 | 1.0218 | 1 | 1.2196 | 1.80E-08 | 0.9897 | 1 | 1.0221 | 1 | 1.018 | 1 | 1.0284 | 1 | 1.0286 | 1 | 0.9734 | 1 | Lifestyle | Time spent watching television |
| p6155_4 | 0.8354 | 1 | 1.1984 | 1 | 1.0915 | 1 | 0.9327 | 1 | 0.9143 | 1 | 0.9845 | 1 | 0.8842 | 1 | 0.8834 | 1 | 0.9158 | 1 | Chemical | Vitamin D supplements |
| p6160_3 | 1.0206 | 1 | 0.967 | 1 | 1.3274 | 8.50E-05 | 0.9846 | 1 | 0.9682 | 1 | 0.9419 | 1 | 0.8907 | 1 | 0.962 | 1 | 1.0355 | 1 | Lifestyle | Attends a religious group frequently |
| p1428_3 | 0.9518 | 1 | 1.0316 | 1 | 0.8902 | 1 | 0.9855 | 1 | 1.002 | 1 | 0.9745 | 1 | 0.8417 | 0.770902 | 1.0085 | 1 | 0.9833 | 1 | Lifestyle | Other spreads/margarine versus rare/non-spread |
| p24503 | 0.9391 | 0.295305 | 0.9715 | 1 | 0.9686 | 1 | 0.9799 | 1 | 0.9568 | 1 | 0.9541 | 1 | 0.982 | 1 | 0.9853 | 1 | 0.885 | 1 | Ecosystems | Greenspace percentage (300 m buffer) |
| p1707_2 | 0.7722 | 1 | 1.0151 | 1 | 0.7827 | 1 | 0.8445 | 1 | 1.387 | 1 | 0.7033 | 1 | 0.9163 | 1 | 0.8064 | 1 | 1.6529 | 1 | Lifestyle | Left-handed versus ambidextrous |
| p1031 | 0.9839 | 1 | 0.9859 | 1 | 1.039 | 1 | 0.9925 | 1 | 0.9315 | 1 | 0.9841 | 1 | 0.9895 | 1 | 0.9915 | 1 | 0.9062 | 1 | Social | Friend/family visits frequently |
| p24502 | 1.0153 | 1 | 1.0031 | 1 | 1.0044 | 1 | 0.988 | 1 | 1.0393 | 1 | 1.0026 | 1 | 1.007 | 1 | 1.0097 | 1 | 1.001 | 1 | Ecosystems | Water percentage (1000 m buffer) |
| p24020 | 1.0059 | 1 | 0.9932 | 1 | 0.9821 | 1 | 1.0163 | 1 | 1.0095 | 1 | 0.9946 | 1 | 1.0213 | 1 | 0.9933 | 1 | 1.0024 | 1 | Physical | Average noise level (daytime) |
| p24021 | 1.0059 | 1 | 0.9931 | 1 | 0.9822 | 1 | 1.0164 | 1 | 1.0096 | 1 | 0.9947 | 1 | 1.0212 | 1 | 0.9933 | 1 | 1.0023 | 1 | Physical | Average noise level (evening) |
| p24022 | 1.006 | 1 | 0.993 | 1 | 0.9822 | 1 | 1.0165 | 1 | 1.0098 | 1 | 0.9947 | 1 | 1.0211 | 1 | 0.9932 | 1 | 1.0019 | 1 | Physical | Average noise level (nighttime) |
| p1508_1 | 0.983 | 1 | 0.9494 | 1 | 0.923 | 1 | 0.9335 | 1 | 0.8 | 1 | 0.9948 | 1 | 0.9777 | 1 | 0.9429 | 1 | 0.9725 | 1 | Lifestyle | Instant coffee versus decaffeinated |
| p1428_1 | 0.9196 | 1 | 1.0378 | 1 | 0.8436 | 1 | 0.9434 | 1 | 0.9896 | 1 | 1.0457 | 1 | 0.8545 | 1 | 1.0036 | 1 | 0.9683 | 1 | Lifestyle | Butter/spreadable butter versus rare/non-spread |
| p1930 | 1.0213 | 1 | 1.0437 | 1 | 0.8504 | 0.035358 | 1.0147 | 1 | 1.1157 | 1 | 0.9689 | 1 | 1.0299 | 1 | 1.0237 | 1 | 1.3687 | 1 | Social | Miserableness |
| p1697_1 | 0.9173 | 1 | 1.0696 | 1 | 1.033 | 1 | 1.0172 | 1 | 1.077 | 1 | 1.0581 | 1 | 0.9898 | 1 | 1.036 | 1 | 1.0697 | 1 | Social | Relatively shorter at 10 years old |
| p1418_5 | 0.8295 | 1 | 1.1145 | 1 | 1.0487 | 1 | 0.9565 | 1 | 0.4648 | 1 | 1.2735 | 1 | 1.0084 | 1 | 1.135 | 1 | NA | NA | Lifestyle | Other milk types versus rare/non-milk |
| p6155_3 | 1.1122 | 1 | 1.0378 | 1 | 1.0923 | 1 | 1.0676 | 1 | 0.9085 | 1 | 1.0811 | 1 | 1.0088 | 1 | 1.046 | 1 | 0.9312 | 1 | Chemical | Vitamin C supplements |
| p24009 | 0.9804 | 1 | 0.9856 | 1 | 0.9445 | 1 | 0.9922 | 1 | 0.9955 | 1 | 0.9583 | 1 | 1.0182 | 1 | 0.9797 | 1 | 1.01 | 1 | Physical | Traffic intensity on nearest road |
| p22036 | 1.0561 | 1 | 0.9437 | 1 | 1.2267 | 0.092973 | 0.9817 | 1 | 0.8028 | 1 | 0.9835 | 1 | 1.0631 | 1 | 1.0181 | 1 | 0.8029 | 1 | Lifestyle | At or above moderate/vigorous/walking recommendation |
| p22032 | 0.9502 | 1 | 0.9981 | 1 | 1.0314 | 1 | 0.9608 | 1 | 0.8618 | 1 | 0.9788 | 1 | 0.978 | 1 | 0.9969 | 1 | 1.0821 | 1 | Lifestyle | IPAQ activity level |
| p1677 | 1.0558 | 1 | 0.9665 | 1 | 1.3092 | 3.05E-06 | 1.004 | 1 | 0.9927 | 1 | 0.997 | 1 | 1.0266 | 1 | 1.0132 | 1 | 0.9636 | 1 | Chemical | Breastfed as a baby |
| p6154_2 | 0.9505 | 1 | 0.9315 | 1 | 0.7582 | 0.001401 | 0.9814 | 1 | 1.0403 | 1 | 1.0459 | 1 | 0.9239 | 1 | 1.0062 | 1 | 1.0668 | 1 | Chemical | Usually takes Ibuprofen |
| p24014 | 0.9678 | 1 | 0.91 | 1 | 0.8874 | 1 | 0.9963 | 1 | 0.9778 | 1 | 0.9674 | 1 | 1.0885 | 1 | 0.9875 | 1 | 1.04 | 1 | Physical | Close to major road |
| p2040 | 1.0696 | 1 | 0.9669 | 1 | 0.9242 | 1 | 1.0609 | 1 | 0.9814 | 1 | 1.1241 | 1 | 1.0284 | 1 | 1.0373 | 1 | 0.8266 | 1 | Social | Risk taking |
| p6155_5 | 1.0514 | 1 | 1.2076 | 1 | 1.2837 | 1 | 0.9985 | 1 | 1.037 | 1 | 1.0786 | 1 | 1.129 | 1 | 1.0462 | 1 | 0.7958 | 1 | Chemical | Vitamin E supplements |
| p6179_3 | 1.0255 | 1 | 1.1194 | 1 | 1.3897 | 0.00341 | 1.0529 | 1 | 0.8391 | 1 | 1.01 | 1 | 1.1642 | 1 | 1.1074 | 1 | 0.9283 | 1 | Chemical | Calcium supplements |
| p1140_1 | 1.0571 | 1 | 0.9547 | 1 | 0.9717 | 1 | 1.034 | 1 | 1.0373 | 1 | 0.9646 | 1 | 1.171 | 1 | 1.0793 | 1 | 1.0201 | 1 | Lifestyle | Less frequent mobile phone use versus no difference |
| p1418_1 | 1.2797 | 1 | 0.8078 | 1 | 0.9651 | 1 | 1.0695 | 1 | 0.845 | 1 | 0.9492 | 1 | 1.0498 | 1 | 0.9457 | 1 | 4.3338 | 1 | Lifestyle | Full-cream milk versus rare/non-milk |
| p22035 | 0.955 | 1 | 0.9862 | 1 | 1.0563 | 1 | 0.9921 | 1 | 0.9125 | 1 | 0.9749 | 1 | 0.969 | 1 | 0.9862 | 1 | 1.0577 | 1 | Lifestyle | At or above moderate/vigorous recommendation |
| p24505 | 0.9683 | 1 | 0.965 | 1 | 1.0221 | 1 | 0.9505 | 0.679679 | 1.0245 | 1 | 0.9773 | 1 | 1.0076 | 1 | 0.9858 | 1 | 0.9412 | 1 | Ecosystems | Water percentage (300 m buffer) |
| p6154_5 | 1.09 | 1 | 1.1121 | 1 | 1.2791 | 0.223685 | 1.0828 | 1 | 1.3079 | 1 | 0.9758 | 1 | 1.021 | 1 | 1.0266 | 1 | 1.5048 | 1 | Chemical | Usually takes Omeprazole |
| p900_5 | 1.0277 | 1 | 1.0237 | 1 | 1.29 | 0.00012 | 1.0379 | 1 | 0.8722 | 1 | 0.9772 | 1 | 1.0506 | 1 | 0.9915 | 1 | 1.2352 | 1 | Lifestyle | Fish eaten above recommendation |
| p1150_1 | 0.8788 | 1 | 1.1165 | 1 | 1.0554 | 1 | 1.0642 | 1 | 0.7177 | 1 | 0.9569 | 1 | 1.0665 | 1 | 0.9754 | 1 | 1.3911 | 1 | Lifestyle | Prefers left side of head for mobile phone use |
| p2149 | 0.9717 | 1 | 1.0113 | 1 | 0.834 | 1.66E-14 | 0.9977 | 1 | 1.0575 | 1 | 1.0376 | 1 | 1.0021 | 1 | 1.01 | 1 | 1.1177 | 1 | Lifestyle | Lifetime number of sexual partners |
| p6179_6 | 1.0154 | 1 | 0.9545 | 1 | 1.1908 | 1 | 0.9327 | 1 | 0.4619 | 1 | 1.0615 | 1 | 1.0411 | 1 | 0.967 | 1 | 1.311 | 1 | Chemical | Selenium supplements |
| p1950 | 1.0228 | 1 | 0.9936 | 1 | 0.9818 | 1 | 1.0088 | 1 | 1.0463 | 1 | 0.9814 | 1 | 1.0123 | 1 | 0.9867 | 1 | 1.1708 | 1 | Social | Sensitivity/hurt feelings |
| p6179_1 | 0.9962 | 1 | 0.937 | 1 | 1.4001 | 1.83E-12 | 0.9064 | 1 | 1.0417 | 1 | 0.8894 | 1 | 0.9782 | 1 | 0.9727 | 1 | 0.8573 | 1 | Chemical | Fish oil (including cod liver oil) supplements |
| p1687_1 | 1.0763 | 1 | 0.9952 | 1 | 0.9756 | 1 | 1.0307 | 1 | 1.1115 | 1 | 0.9852 | 1 | 1.056 | 1 | 1.0478 | 1 | 0.8811 | 1 | Social | Relatively thinner at 10 years old |
| p6155_7 | 1.0084 | 1 | 0.9492 | 1 | 0.9701 | 1 | 0.9738 | 1 | 0.9785 | 1 | 1.0283 | 1 | 0.9718 | 1 | 0.9997 | 1 | 0.8941 | 1 | Chemical | Multivitamins +/- minerals supplements |
| p894 | 1.0223 | 1 | 1.0124 | 1 | 1.1382 | 1.19E-07 | 1.0189 | 1 | 0.9659 | 1 | 0.9833 | 1 | 1.0023 | 1 | 0.989 | 1 | 1.0211 | 1 | Lifestyle | Duration of moderate activity |
| p24504 | 1.0069 | 1 | 1.022 | 1 | 1.0294 | 1 | 1.0132 | 1 | 1.0782 | 1 | 0.9866 | 1 | 0.9891 | 1 | 1.0143 | 1 | 1.0136 | 1 | Ecosystems | Domestic garden percentage (300 m buffer) |
| p24010 | 1.018 | 1 | 1.0113 | 1 | 1.0102 | 1 | 1.0041 | 1 | 1.0468 | 1 | 0.9865 | 1 | 1.0086 | 1 | 0.9997 | 1 | 1.0229 | 1 | Physical | Inverse distance to road |
| p1170 | 1.0385 | 1 | 0.9598 | 1 | 1.2208 | 5.01E-11 | 0.9873 | 1 | 0.9036 | 1 | 0.9677 | 1 | 1.0337 | 1 | 1.0042 | 1 | 0.7994 | 1 | Lifestyle | Gets up more easily in the morning |
| p1498 | 0.9509 | 1 | 1.0124 | 1 | 1.0594 | 1 | 0.9734 | 1 | 1.0283 | 1 | 0.9852 | 1 | 0.9973 | 1 | 0.9828 | 1 | 0.9696 | 1 | Lifestyle | Coffee intake |
| p981 | 0.9964 | 1 | 1.0016 | 1 | 1.0179 | 1 | 1.0077 | 1 | 1.0193 | 1 | 0.9862 | 1 | 1.0097 | 1 | 1.0003 | 1 | 1.0573 | 1 | Lifestyle | Duration of walking for pleasure |
| p24501 | 0.9937 | 1 | 1.0317 | 1 | 1.0164 | 1 | 0.9982 | 1 | 1.0497 | 1 | 0.9873 | 1 | 0.9897 | 1 | 1.0179 | 1 | 1.0297 | 1 | Ecosystems | Domestic garden percentage (1000 m buffer) |
| p1249 | 1.0133 | 1 | 0.9915 | 1 | 1.0828 | 0.000327 | 1.0068 | 1 | 0.9958 | 1 | 0.9942 | 1 | 1.0148 | 1 | 1.0014 | 1 | 1.0497 | 1 | Lifestyle | Past tobacco smoking frequency |
| p6145_3 | 0.9764 | 1 | 0.9101 | 1 | 0.9525 | 1 | 0.9414 | 1 | 0.9565 | 1 | 0.9141 | 1 | 1.0221 | 1 | 0.9408 | 1 | 1.0339 | 1 | Social | Death of a close relative |

**Notes:** P represents the Bonferroni-adjusted P value. ADRD = Alzheimer’s Disease and Related Dementias.

**Supplementary Table 11. Associations between 193 exposures and AD in subgroups (by sex, age, genetics, and ethnicity)**

| **Variable Code** | **Female** | | **Male** | | **Age<65** | | **Age>=65** | | **Low genetic risk** | | **Intermediate genetic risk** | | **High genetic risk** | | **White British** | | **Other**  **ethnicities** | | **Category** | **Variable** |
| --- | --- | --- | --- | --- | --- | --- | --- | --- | --- | --- | --- | --- | --- | --- | --- | --- | --- | --- | --- | --- |
|  | **HR** | **P** | **HR** | **P** | **HR** | **P** | **HR** | **P** | **HR** | **P** | **HR** | **P** | **HR** | **P** | **HR** | **P** | **HR** | **P** |  |  |
| p1080 | 0.8235 | 2.70704E-12 | 0.8365 | 1.91391E-09 | 0.7608 | 4.83359E-18 | 0.8321 | 4.64154E-13 | 0.8521 | 1 | 0.8015 | 9.86779E-10 | 0.8373 | 4.45015E-12 | 0.842 | 1.26396E-17 | 0.8441 | 1 | Lifestyle | Time spent using computer |
| p1100 | 0.6513 | 3.32444E-10 | 0.7199 | 1.28583E-12 | 0.6074 | 1.10913E-21 | 0.713 | 6.8645E-11 | 0.5205 | 0.008352863 | 0.7866 | 0.004837496 | 0.7446 | 3.78285E-09 | 0.747 | 6.8298E-15 | 0.6734 | 1 | Lifestyle | Frequent motorway speeding |
| p738 | 0.8545 | 1.53537E-05 | 0.8864 | 0.001630244 | 0.6776 | 6.16994E-36 | 0.8776 | 0.000251109 | 0.8603 | 1 | 0.8469 | 0.000460962 | 0.9064 | 0.019016063 | 0.8785 | 6.25667E-08 | 0.8985 | 1 | Social | Total household income |
| p6144_1 | 1.8151 | 5.05726E-05 | 1.8423 | 7.30449E-05 | 2.1844 | 1.30819E-06 | 1.7432 | 3.36055E-05 | 1.3905 | 1 | 2.0286 | 0.000137266 | 1.8441 | 1.39229E-06 | 1.8654 | 1.4096E-10 | 1.6556 | 1 | Lifestyle | Never eat eggs |
| p6146 | 1.5125 | 0.00196243 | 1.6025 | 8.39889E-05 | 2.0231 | 8.59158E-09 | 1.6393 | 2.34874E-07 | 2.0537 | 0.542092357 | 1.8672 | 1.82277E-06 | 1.4253 | 0.010414767 | 1.6319 | 7.52411E-11 | 1.562 | 1 | Social | Disability |
| p6138 | 1.3949 | 3.1418E-06 | 1.432 | 8.12708E-07 | 1.5215 | 4.54504E-08 | 1.4039 | 2.04515E-07 | 1.7416 | 0.61027842 | 1.5404 | 2.91671E-06 | 1.2811 | 0.000697379 | 1.4393 | 2.9874E-14 | 1.3289 | 1 | Social | Lower education |
| p728 | 0.8256 | 9.55043E-05 | 0.8169 | 2.89816E-05 | 0.7477 | 4.73561E-12 | 0.7892 | 4.18334E-08 | 0.6197 | 0.011605398 | 0.8104 | 0.00202983 | 0.8363 | 5.79988E-05 | 0.8256 | 1.84895E-09 | 0.7753 | 1 | Social | Number of vehicles in household |
| p680 | 1.6674 | 1.93323E-07 | 1.531 | 0.000344647 | 1.1549 | 1 | 1.5845 | 1.72978E-06 | 1.4032 | 1 | 1.8193 | 8.39191E-07 | 1.3992 | 0.006759958 | 1.6138 | 1.55884E-11 | 1.025 | 1 | Social | Rent accommodation versus own |
| p6144_3 | 1.3915 | 1 | 2.195 | 2.6468E-08 | 1.6376 | 0.277157059 | 1.9422 | 1.96926E-08 | 1.1182 | 1 | 1.4644 | 1 | 1.9323 | 4.07552E-08 | 1.8367 | 8.74039E-10 | 0.4392 | 1 | Lifestyle | Never eat wheat products |
| p1200 | 0.8051 | 1.55568E-05 | 0.876 | 0.154620025 | 0.8833 | 1 | 0.8843 | 0.147109873 | 0.881 | 1 | 0.891 | 1 | 0.8254 | 2.38859E-05 | 0.8399 | 4.4765E-07 | 0.7316 | 1 | Lifestyle | Experiences insomnia frequently |
| p6162_1 | 0.7929 | 0.022258397 | 0.7097 | 2.17849E-05 | 0.693 | 4.9085E-05 | 0.7684 | 0.000680705 | 0.5271 | 0.05539929 | 0.7743 | 0.21652925 | 0.7932 | 0.009065065 | 0.7351 | 2.48541E-09 | 0.7721 | 1 | Lifestyle | Uses car/motor vehicle as transport |
| p2000 | 0.7327 | 3.34517E-06 | 0.8124 | 0.135708968 | 0.6914 | 3.48166E-06 | 0.8056 | 0.009425546 | 0.8336 | 1 | 0.8063 | 0.570184573 | 0.8207 | 0.030973946 | 0.7889 | 4.60581E-06 | 0.7461 | 1 | Social | Worry too long after embarrassment |
| p6144_4 | 1.4916 | 1.43314E-09 | 1.1097 | 1 | 1.9313 | 1.23775E-18 | 1.2143 | 0.102155939 | 1.6216 | 1 | 1.3552 | 0.014147695 | 1.2504 | 0.013110264 | 1.2882 | 3.56168E-06 | 1.0198 | 1 | Lifestyle | Never eat sugar foods |
| p1508_2 | 0.8697 | 1 | 0.7677 | 0.556483458 | 0.6908 | 0.022807651 | 0.7429 | 0.023919797 | 0.6481 | 1 | 0.8872 | 1 | 0.7669 | 0.108088149 | 0.755 | 0.001134724 | 0.8246 | 1 | Lifestyle | Ground coffee versus decaffeinated |
| p1090 | 0.8931 | 0.00059415 | 0.9318 | 1 | 0.8697 | 0.000600738 | 0.8756 | 9.67274E-06 | 0.7103 | 0.001248628 | 0.8824 | 0.039387129 | 0.904 | 0.006363486 | 0.8883 | 3.62414E-07 | 0.9312 | 1 | Lifestyle | Time spent driving |
| p6164_2 | 0.7699 | 0.000837558 | 0.7722 | 0.004434622 | 0.6973 | 1.36985E-05 | 0.7725 | 0.00033238 | 0.7535 | 1 | 0.7053 | 0.000628707 | 0.7431 | 5.45714E-06 | 0.7947 | 1.53029E-05 | 1.101 | 1 | Lifestyle | Engages in other exercises |
| p6164_4 | 0.8706 | 1 | 0.8712 | 1 | 0.8418 | 1 | 0.9139 | 1 | 0.679 | 1 | 0.8663 | 1 | 0.9077 | 1 | 0.8103 | 4.9731E-05 | 0.9564 | 1 | Lifestyle | Light DIY |
| p1110 | 0.9348 | 0.064700854 | 0.9604 | 1 | 0.8174 | 7.67289E-18 | 0.9272 | 0.000825539 | 0.944 | 1 | 0.9223 | 0.089758582 | 0.9423 | 0.073970226 | 0.952 | 0.057968414 | 0.9026 | 1 | Lifestyle | Length of mobile phone use |
| p6164_5 | 0.8554 | 1 | 0.8072 | 0.045282387 | 0.93 | 1 | 0.8651 | 1 | 0.508 | 0.061884915 | 0.757 | 0.037898432 | 0.8841 | 1 | 0.8767 | 0.409274051 | 0.6257 | 1 | Lifestyle | Heavy DIY |
| p24006 | 1.1404 | 0.000168795 | 1.0627 | 1 | 1.0855 | 1 | 1.0974 | 0.081784516 | 1.2569 | 0.928664742 | 1.1098 | 0.583585444 | 1.1228 | 0.001240685 | 1.1058 | 0.000178072 | 1.1139 | 1 | Chemical | PM2.5 pollution |
| p2400_1 | 1.1433 | 0.000773112 | 1.0503 | 1 | 1.0309 | 1 | 1.1045 | 0.046214459 | 1.1626 | 1 | 1.052 | 1 | 1.127 | 0.001552748 | 1.0777 | 0.096022109 | 1.0298 | 1 | Chemical | Nitrogen dioxide/oxides air pollution |
| p6142 | 1.4084 | 1 | 1.4013 | 1 | 1.0309 | 1 | 1.6718 | 1 | 1.5062 | 1 | 2.0963 | 0.000123217 | 1.225 | 1 | 1.4408 | 0.087319758 | 0.6527 | 1 | Social | Unemployed |
| p2492 | 1.1863 | 0.852840915 | 1.1762 | 1 | 1.9512 | 6.11237E-20 | 1.2064 | 0.169855575 | 1.8838 | 0.279147758 | 1.3715 | 0.010545152 | 1.1091 | 1 | 1.2558 | 7.91017E-05 | 1.4356 | 1 | Chemical | Takes other prescription medications |
| p1269 | 1.3668 | 0.113430077 | 1.1715 | 1 | 1.2551 | 1 | 1.2457 | 1 | 1.4436 | 1 | 1.3613 | 1 | 1.2328 | 1 | 1.3345 | 0.004870033 | 1.314 | 1 | Chemical | Exposure to tobacco smoke at home |
| p6141 | 0.8856 | 1 | 0.8476 | 1 | 0.9147 | 1 | 0.8306 | 0.178425229 | 0.7685 | 1 | 0.7065 | 0.000702346 | 0.9228 | 1 | 0.8848 | 1 | 0.6701 | 1 | Social | Lives with partner |
| p1548 | 1.02 | 1 | 1.1179 | 1 | 1.1295 | 1 | 1.1327 | 0.746580552 | 1.0847 | 1 | 1.1694 | 1 | 1.0782 | 1 | 1.1428 | 0.020775006 | 1.226 | 1 | Lifestyle | Diet varies frequently |
| p6160_1 | 0.7729 | 0.021392382 | 0.9189 | 1 | 0.7407 | 0.016644966 | 0.8366 | 0.763415586 | 1.0693 | 1 | 0.745 | 0.141988741 | 0.7787 | 0.010582513 | 0.8493 | 0.161039145 | 1.0955 | 1 | Lifestyle | Attends a sports club or gym frequently |
| p1488 | 0.9633 | 1 | 0.9688 | 1 | 0.9416 | 1 | 0.9651 | 1 | 0.8488 | 1 | 0.9392 | 1 | 0.9209 | 0.171834185 | 0.9387 | 0.3515278 | 0.8348 | 1 | Lifestyle | Tea intake |
| p6144_2 | 1.4922 | 0.329792461 | 1.4929 | 0.995560512 | 1.8371 | 0.023139729 | 1.5654 | 0.02520945 | 1.3182 | 1 | 1.2628 | 1 | 1.698 | 0.000375615 | 1.6241 | 9.75357E-05 | 1.604 | 1 | Lifestyle | Never eat dairy products |
| p2159 | 1.4137 | 0.050164271 | 1.1088 | 1 | 1.0294 | 1 | 1.4212 | 0.009583095 | 1.9055 | 1 | 1.2343 | 1 | 1.2559 | 1 | 1.3486 | 0.009576443 | 1.4477 | 1 | Lifestyle | Had same-sex intercourse |
| p137 | 1.0971 | 1 | 1.0837 | 1 | 1.4851 | 3.57622E-19 | 1.0969 | 1 | 1.1992 | 1 | 1.2414 | 0.001886851 | 1.0505 | 1 | 1.1456 | 9.397E-05 | 1.2028 | 1 | Chemical | Number of medications taken |
| p2060 | 1.146 | 0.501221108 | 1.1715 | 0.140196925 | 0.9377 | 1 | 1.2012 | 0.003097819 | 1.2159 | 1 | 1.0969 | 1 | 1.1764 | 0.016214444 | 1.1845 | 0.000144201 | 0.9047 | 1 | Social | Feels unenthusiasm/disinterest frequently |
| p900_1 | 1.0887 | 1 | 1.1798 | 0.924015222 | 1.1991 | 1 | 1.1114 | 1 | 1.3733 | 1 | 1.2982 | 0.059424827 | 1.083 | 1 | 1.1157 | 1 | 1.0593 | 1 | Lifestyle | Improper consumption of alcohol |
| p6154_6 | 1.147 | 1 | 1.733 | 0.104703955 | 1.875 | 0.001154495 | 1.244 | 1 | 2.0119 | 1 | 1.4523 | 1 | 1.4263 | 0.305361019 | 1.3233 | 0.649019568 | 1.1262 | 1 | Chemical | Usually takes Laxatives |
| p6145_6 | 1.349 | 0.633865696 | 1.2106 | 1 | 0.8509 | 1 | 0.9517 | 1 | 1.4934 | 1 | 1.51 | 0.123573769 | 1.0903 | 1 | 1.3189 | 0.06769739 | 1.0645 | 1 | Social | Financial difficulties |
| p6160_4 | 0.6995 | 0.051037127 | 0.7264 | 1 | 1.0935 | 1 | 0.663 | 0.021940322 | 0.4406 | 1 | 0.9929 | 1 | 0.7056 | 0.151551029 | 0.8149 | 1 | 0.9181 | 1 | Lifestyle | Attends a adult education class frequently |
| p2139 | 1.0717 | 1 | 1.2605 | 0.014901741 | 0.8261 | 0.635294113 | 1.1333 | 1 | 1.297 | 1 | 1.2746 | 0.144777037 | 1.1245 | 1 | 1.1935 | 0.006580068 | 0.8999 | 1 | Lifestyle | First sexual intercourse (< age 18) |
| p20116_2 | 1.4849 | 0.015768559 | 1.0675 | 1 | 1.0242 | 1 | 1.1858 | 1 | 1.5861 | 1 | 1.454 | 0.308209652 | 1.1442 | 1 | 1.4249 | 0.000188508 | 1.4474 | 1 | Lifestyle | Current smoker |
| p1239 | 1.2024 | 0.101548692 | 1.048 | 1 | 0.9856 | 1 | 1.078 | 1 | 1.2667 | 1 | 1.2396 | 0.080004617 | 1.0497 | 1 | 1.1986 | 0.000256109 | 1.3219 | 1 | Lifestyle | Current tobacco smoking frequency |
| p2050 | 1.1112 | 1 | 1.1893 | 0.117964609 | 0.9887 | 1 | 1.1604 | 0.18393982 | 1.3153 | 1 | 1.0787 | 1 | 1.1864 | 0.011287868 | 1.211 | 3.21704E-06 | 1.0195 | 1 | Social | Feels depressed mood frequently |
| p943 | 0.9968 | 1 | 0.9707 | 1 | 1.0006 | 1 | 0.9601 | 1 | 1.0013 | 1 | 0.9768 | 1 | 0.9723 | 1 | 0.9733 | 1 | 0.8639 | 1 | Lifestyle | Climbes stair frequently |
| p6139_3 | 0.7084 | 0.867205616 | 0.751 | 1 | 0.5664 | 0.0177666 | 0.8355 | 1 | 0.6269 | 1 | 0.8308 | 1 | 0.8199 | 1 | 0.7717 | 0.538619464 | 0.7456 | 1 | Chemical | Uses open solid fuel fire in winter |
| p1528 | 1.1074 | 0.608395652 | 1.1013 | 0.166214523 | 0.9868 | 1 | 1.1218 | 0.017308212 | 1.065 | 1 | 1.049 | 1 | 1.095 | 0.299963171 | 1.0658 | 0.690530187 | 1.5412 | 1 | Lifestyle | Water intake |
| p2277 | 1.0211 | 1 | 1.12 | 0.084508832 | 0.9678 | 1 | 1.0615 | 1 | 1.1506 | 1 | 1.1239 | 0.377214028 | 1.0467 | 1 | 1.0862 | 0.065597369 | 0.8563 | 1 | Physical | Uses solarium/sunlamp frequently |
| p2070 | 1.0893 | 1 | 1.2035 | 0.019222764 | 0.9375 | 1 | 1.1463 | 0.423416976 | 1.1182 | 1 | 1.1128 | 1 | 1.0869 | 1 | 1.1302 | 0.093104801 | 1.1733 | 1 | Social | Feels tenseness/restlessness frequently |
| p2020 | 1.1392 | 1 | 1.3076 | 0.16218268 | 1.0857 | 1 | 1.1739 | 1 | 1.5872 | 1 | 1.3329 | 0.236225865 | 1.1914 | 1 | 1.2277 | 0.020210938 | 1.0641 | 1 | Social | Loneliness, isolation |
| p709 | 0.9365 | 1 | 0.9583 | 1 | 0.7389 | 1.28217E-20 | 0.9109 | 1 | 0.8182 | 1 | 0.9112 | 1 | 0.9833 | 1 | 0.9317 | 1 | 0.9546 | 1 | Social | Number in household |
| p1279 | 1.1563 | 1 | 1.1373 | 1 | 1.1457 | 1 | 1.1608 | 1 | 1.3746 | 1 | 1.4075 | 0.000873055 | 0.9496 | 1 | 1.1622 | 0.183484487 | 1.021 | 1 | Chemical | Exposure to tobacco smoke outside home |
| p1259 | 1.2914 | 0.524251559 | 1.0999 | 1 | 0.9713 | 1 | 1.0974 | 1 | 1.2891 | 1 | 1.1726 | 1 | 1.1412 | 1 | 1.1875 | 1 | 1.4576 | 1 | Chemical | Had smoking/smokers in household |
| p1980 | 1.0471 | 1 | 1.1153 | 1 | 1.1434 | 1 | 1.0726 | 1 | 1.383 | 1 | 1.0699 | 1 | 1.1033 | 1 | 1.0944 | 1 | 0.867 | 1 | Social | Worrier/anxious feelings |
| p24007 | 1.0822 | 0.843091676 | 1.0128 | 1 | 0.9834 | 1 | 1.0531 | 1 | 1.1121 | 1 | 1.0059 | 1 | 1.0593 | 1 | 1.031 | 1 | 0.9571 | 1 | Chemical | PM2.5 absorbance pollution |
| p24506 | 0.919 | 0.189149629 | 0.9813 | 1 | 0.9867 | 1 | 0.947 | 1 | 0.9456 | 1 | 0.9446 | 1 | 0.9258 | 0.331381065 | 0.9381 | 0.412893766 | 0.9734 | 1 | Ecosystems | Natural environment percentage (1000m buffer) |
| p1920 | 1.1324 | 1 | 1.092 | 1 | 0.9259 | 1 | 1.1264 | 1 | 1.364 | 1 | 1.1582 | 1 | 1.1933 | 0.12459452 | 1.1788 | 0.016875761 | 1.2155 | 1 | Social | Mood swings |
| p2110 | 0.9641 | 1 | 0.9766 | 1 | 0.9633 | 1 | 0.966 | 1 | 0.9806 | 1 | 0.9552 | 1 | 0.9793 | 1 | 0.974 | 1 | 0.987 | 1 | Social | Able to confide frequently |
| p6160_5 | 0.8831 | 1 | 0.9157 | 1 | 1.2078 | 1 | 0.8692 | 1 | 1.0095 | 1 | 0.9407 | 1 | 0.8761 | 1 | 0.8757 | 1 | 1.3432 | 1 | Lifestyle | Attends other group activities frequently |
| p22036 | 1.1037 | 1 | 1.0849 | 1 | 1.502 | 0.006372984 | 1.0512 | 1 | 0.7438 | 1 | 1.1971 | 1 | 1.2244 | 1 | 1.1713 | 1 | 1.0226 | 1 | Lifestyle | At or above moderate/vigorous/walking recommendation |
| p1140_2 | 0.8181 | 0.258650677 | 0.9228 | 1 | 0.6772 | 1.7524E-05 | 1.0051 | 1 | 0.5348 | 1 | 0.9693 | 1 | 0.9183 | 1 | 0.8971 | 1 | 0.8587 | 1 | Lifestyle | More frequent mobile phone use versus no difference |
| p6162_4 | 0.764 | 1 | 0.8416 | 1 | 0.523 | 0.019194094 | 0.7834 | 1 | 0.6423 | 1 | 0.7523 | 1 | 0.9654 | 1 | 0.8371 | 1 | 0.617 | 1 | Lifestyle | Uses cycle as transport |
| p1757 | 1.0555 | 1 | 1.1392 | 1 | 0.9965 | 1 | 1.0545 | 1 | 1.1202 | 1 | 1.0965 | 1 | 1.0772 | 1 | 1.0271 | 1 | 1.4831 | 1 | Physical | Degree of facial ageing |
| p1518 | 0.9068 | 1 | 0.9281 | 1 | 0.9598 | 1 | 0.9207 | 1 | 0.7977 | 1 | 0.9257 | 1 | 0.9349 | 1 | 0.904 | 0.87682233 | 0.6053 | 0.4441772 | Lifestyle | Hot drink temperature |
| p6145_2 | 0.8355 | 1 | 0.8306 | 1 | 0.5905 | 0.001604337 | 0.9346 | 1 | 0.7542 | 1 | 0.8198 | 1 | 0.8691 | 1 | 0.766 | 0.11954834 | 1.1615 | 1 | Social | Relativeâ€™s serious illness or injury |
| p2030 | 0.7949 | 0.034258511 | 1.0142 | 1 | 0.8507 | 1 | 0.93 | 1 | 1.0212 | 1 | 1.1197 | 1 | 0.8605 | 1 | 0.9452 | 1 | 0.9219 | 1 | Social | Guilty feelings |
| p24505 | 0.9418 | 1 | 0.9328 | 1 | 0.9938 | 1 | 0.9222 | 0.245620575 | 0.9723 | 1 | 0.9365 | 1 | 0.9433 | 1 | 0.9279 | 0.04162202 | 0.977 | 1 | Ecosystems | Water percentage (300 m buffer) |
| p2267 | 0.9561 | 1 | 0.9732 | 1 | 0.9018 | 0.718839212 | 0.9833 | 1 | 0.8654 | 1 | 0.9322 | 1 | 0.9614 | 1 | 0.96 | 1 | 0.9032 | 1 | Physical | Uses sun or UV protection frequently |
| p6154_1 | 1.1597 | 1 | 1.0253 | 1 | 1.896 | 1.82751E-13 | 1.1118 | 1 | 0.947 | 1 | 1.2098 | 1 | 1.0554 | 1 | 1.1147 | 1 | 1.8489 | 1 | Chemical | Usually takes Aspirin |
| p900_4 | 1.1324 | 1 | 1.2481 | 0.020994899 | 1.3697 | 0.000203327 | 1.1811 | 0.306695669 | 1.027 | 1 | 1.2226 | 0.987979748 | 1.0926 | 1 | 1.163 | 0.061504582 | 1.0652 | 1 | Lifestyle | Fruit eaten above recommendation |
| p1787 | 1.068 | 1 | 0.9912 | 1 | 0.9063 | 1 | 1.1272 | 1 | 0.9615 | 1 | 1.1467 | 1 | 1.063 | 1 | 1.0737 | 1 | 1.5059 | 1 | Chemical | Maternal smoking around birth |
| p6179_5 | 1.1322 | 1 | 1.4093 | 1 | 0.9364 | 1 | 1.1556 | 1 | 3.0202 | 0.157548969 | 1.0369 | 1 | 1.1215 | 1 | 1.1904 | 1 | 1.5279 | 1 | Chemical | Iron supplements |
| p670_1 | 1.2475 | 1 | 1.2236 | 1 | 1.0278 | 1 | 1.2371 | 1 | 1.2136 | 1 | 1.2822 | 1 | 1.255 | 1 | 1.1683 | 1 | 1.2798 | 1 | Social | Lives in a flat versus house |
| p670_2 | 1.4224 | 1 | 1.6332 | 1 | 2.3267 | 0.505231679 | 1.6137 | 1 | 1.4573 | 1 | 2.0123 | 1 | 1.5424 | 1 | 1.3942 | 1 | 1.9824 | 1 | Social | Lives in mobile structure versus house |
| p1359 | 0.9259 | 1 | 0.9953 | 1 | 0.9202 | 1 | 0.9803 | 1 | 1.1498 | 1 | 0.9689 | 1 | 0.945 | 1 | 0.9514 | 1 | 1.0036 | 1 | Lifestyle | Poultry intake |
| p900_6 | 0.6539 | 1 | 0.6761 | 1 | 0.5308 | 1 | 0.6739 | 1 | 1.4975 | 1 | 0.8236 | 1 | 0.6212 | 1 | 0.7731 | 1 | 0.4601 | 1 | Lifestyle | Whole bread/refined cereals intake reduced |
| p900_7 | 0.9863 | 1 | 0.9246 | 1 | 1.096 | 1 | 0.9469 | 1 | 0.8332 | 1 | 0.8151 | 0.72417619 | 1.0163 | 1 | 0.9446 | 1 | 0.9935 | 1 | Lifestyle | Wholegrain eaten above recommendation |
| p1060 | 1.0523 | 1 | 1.0694 | 1 | 1.2638 | 1.00797E-05 | 1.0017 | 1 | 0.953 | 1 | 0.9952 | 1 | 1.126 | 0.051515357 | 1.0865 | 0.232321042 | 1.0095 | 1 | Physical | Time spent outdoors (winter) |
| p2040 | 0.9824 | 1 | 0.8805 | 1 | 0.818 | 1 | 0.9625 | 1 | 0.8947 | 1 | 0.9785 | 1 | 1.0164 | 1 | 0.8982 | 1 | 0.7968 | 1 | Social | Risk taking |
| p6155_4 | 0.7859 | 1 | 1.0007 | 1 | 0.9586 | 1 | 0.8032 | 1 | 0.6257 | 1 | 1.0221 | 1 | 0.7381 | 1 | 0.7378 | 0.964374274 | 1.464 | 1 | Chemical | Vitamin D supplements |
| p900_3 | 1.162 | 1 | 1.1584 | 1 | 1.3011 | 1 | 1.1135 | 1 | 0.4691 | 1 | 1.2942 | 1 | 1.3853 | 1 | 1.3245 | 1 | 1.469 | 1 | Lifestyle | Vegetable eaten above recommendation |
| p6155_3 | 1.0504 | 1 | 1.2216 | 1 | 1.2296 | 1 | 1.0638 | 1 | 1.3823 | 1 | 1.2037 | 1 | 0.9354 | 1 | 1.1182 | 1 | 1.2288 | 1 | Chemical | Vitamin C supplements |
| p1050 | 1.0628 | 1 | 1.0564 | 1 | 1.2501 | 2.10615E-07 | 0.9861 | 1 | 0.9411 | 1 | 1.0361 | 1 | 1.063 | 1 | 1.0528 | 1 | 1.0115 | 1 | Physical | Time spent outdoors (summer) |
| p1408 | 0.9765 | 1 | 0.9718 | 1 | 0.9549 | 1 | 0.9355 | 1 | 0.8929 | 1 | 0.9413 | 1 | 0.9726 | 1 | 0.963 | 1 | 1.0066 | 1 | Lifestyle | Cheese intake |
| p1210 | 1.0739 | 1 | 0.8268 | 0.202318796 | 0.9604 | 1 | 0.8594 | 1 | 0.795 | 1 | 0.9186 | 1 | 0.9735 | 1 | 0.9423 | 1 | 0.8077 | 1 | Lifestyle | Snoring |
| p6154_4 | 1.3159 | 1 | 1.2717 | 1 | 1.2453 | 1 | 1.0903 | 1 | 1.7248 | 1 | 1.4296 | 1 | 1.0452 | 1 | 1.3215 | 1 | 1.3177 | 1 | Chemical | Usually takes Ranitidine |
| p2400_2 | 1.0511 | 1 | 1.0087 | 1 | 0.9762 | 1 | 1.0385 | 1 | 1.0929 | 1 | 0.9745 | 1 | 1.0536 | 1 | 1.0152 | 1 | 0.9347 | 1 | Chemical | PM10 pollution |
| p6155_7 | 1.0743 | 1 | 1.0018 | 1 | 0.989 | 1 | 1.0301 | 1 | 1.2142 | 1 | 1.2569 | 0.913753228 | 0.9237 | 1 | 1.0991 | 1 | 0.7958 | 1 | Chemical | Multivitamins +/- minerals supplements |
| p1737 | 0.9867 | 1 | 0.9867 | 1 | 0.8491 | 9.01241E-05 | 0.9362 | 1 | 1.0256 | 1 | 0.9892 | 1 | 0.9615 | 1 | 0.996 | 1 | 0.9494 | 1 | Physical | Childhood sunburn occurred frequently |
| p2080 | 1.0747 | 1 | 1.0413 | 1 | 0.8938 | 1 | 1.0744 | 1 | 1.3363 | 0.588474082 | 1.1055 | 1 | 1.0553 | 1 | 1.074 | 1 | 0.9902 | 1 | Social | Feels tiredness/lethargy frequently |
| p6179_1 | 1.0359 | 1 | 1.0513 | 1 | 1.5671 | 9.92022E-10 | 0.9531 | 1 | 0.982 | 1 | 1.0217 | 1 | 0.9805 | 1 | 1.0969 | 1 | 0.7261 | 1 | Chemical | Fish oil (including cod liver oil) supplements |
| p6179_4 | 0.8059 | 1 | 0.6768 | 1 | 1.04 | 1 | 0.7235 | 1 | 1.5945 | 1 | 0.8857 | 1 | 0.778 | 1 | 0.8366 | 1 | 0.4709 | 1 | Chemical | Zinc supplements |
| p1150_2 | 1.0416 | 1 | 1.372 | 1 | 1.4215 | 1 | 1.0679 | 1 | 0.5803 | 1 | 1.1632 | 1 | 1.0877 | 1 | 1.0907 | 1 | 1.0015 | 1 | Lifestyle | Prefers right side of head for mobile phone use |
| p2149 | 0.9258 | 1 | 0.9644 | 1 | 0.7777 | 3.20356E-11 | 0.9465 | 1 | 1.0664 | 1 | 0.9775 | 1 | 0.9652 | 1 | 0.9618 | 1 | 0.9067 | 1 | Lifestyle | Lifetime number of sexual partners |
| p24012 | 1.0418 | 1 | 1.021 | 1 | 0.9813 | 1 | 1.0195 | 1 | 1.0032 | 1 | 1.0049 | 1 | 1.0681 | 1 | 1.0053 | 1 | 1.023 | 1 | Physical | Inverse distance to major road |
| p22039 | 0.9564 | 1 | 1.0201 | 1 | 0.9555 | 1 | 0.9762 | 1 | 1.0175 | 1 | 1.0129 | 1 | 0.9299 | 0.780673264 | 1.0169 | 1 | 0.9864 | 1 | Lifestyle | MET minutes/week of vigorous activity |
| p874 | 1.0385 | 1 | 1.0883 | 0.556977284 | 1.0794 | 1 | 1.0545 | 1 | 0.9993 | 1 | 1.0545 | 1 | 1.0422 | 1 | 1.0708 | 0.194300588 | 0.9939 | 1 | Lifestyle | Duration of walks |
| p20116_1 | 1.0694 | 1 | 0.9996 | 1 | 1.1821 | 1 | 1.0708 | 1 | 1.2343 | 1 | 1.008 | 1 | 1.0507 | 1 | 1.0942 | 1 | 1.0061 | 1 | Lifestyle | Previous smoker |
| p900_9 | 1.0433 | 1 | 0.9773 | 1 | 1.117 | 1 | 0.9856 | 1 | 0.8377 | 1 | 1.0352 | 1 | 1.0832 | 1 | 1.0201 | 1 | 0.7443 | 1 | Lifestyle | Processed meat intake reduced |
| p24500 | 0.95 | 1 | 0.9913 | 1 | 0.9952 | 1 | 0.9808 | 1 | 0.8764 | 1 | 0.9836 | 1 | 0.9474 | 1 | 0.9701 | 1 | 1.1043 | 1 | Ecosystems | Greenspace percentage (1000 m buffer) |
| p1697_2 | 0.9358 | 1 | 0.8767 | 1 | 0.8521 | 1 | 0.9216 | 1 | 0.7934 | 1 | 1.0201 | 1 | 0.9231 | 1 | 0.9229 | 1 | 1.1516 | 1 | Social | Relatively taller at 10 years old |
| p699 | 1.0144 | 1 | 0.9827 | 1 | 1.3538 | 2.34092E-13 | 0.9823 | 1 | 0.9091 | 1 | 0.9531 | 1 | 0.9831 | 1 | 0.9798 | 1 | 1.0659 | 1 | Social | Length of residence at current address |
| p6164_3 | 0.9496 | 1 | 0.9704 | 1 | 0.4057 | 1.02369E-06 | 0.9882 | 1 | 1.8449 | 1 | 0.8092 | 1 | 0.9222 | 1 | 0.9347 | 1 | 0.4058 | 1 | Lifestyle | Engages in strenuous sports |
| p1428_1 | 0.8096 | 1 | 1.0931 | 1 | 0.7955 | 1 | 0.869 | 1 | 0.7325 | 1 | 0.9608 | 1 | 0.8106 | 1 | 0.8848 | 1 | 0.897 | 1 | Lifestyle | Butter/spreadable butter versus rare/non-spread |
| p6145_4 | 1.3323 | 1 | 1.3152 | 1 | 1.8457 | 0.589243487 | 1.3789 | 1 | 2.1482 | 1 | 1.5104 | 1 | 1.0243 | 1 | 1.1111 | 1 | 1.5761 | 1 | Social | Death of a spouse or partner |
| p1767 | 0.9384 | 1 | 1.3638 | 1 | 1.3902 | 1 | 1.1947 | 1 | 2.7315 | 1 | 0.9249 | 1 | 1.3197 | 1 | 1.3252 | 1 | 2.3763 | 1 | Social | Adopted as a child |
| p1418_4 | 0.8041 | 1 | 0.5701 | 1 | 0.7799 | 1 | 0.7616 | 1 | NA | NA | 0.6663 | 1 | 0.9437 | 1 | 0.8173 | 1 | NA | NA | Lifestyle | Soya milk versus rare/non-milk |
| p1418_5 | 0.8305 | 1 | 1.1937 | 1 | 1.2562 | 1 | 1.0699 | 1 | NA | NA | 1.9845 | 1 | 1.2969 | 1 | 1.3635 | 1 | NA | NA | Lifestyle | Other milk types versus rare/non-milk |
| p1130 | 1.0245 | 1 | 0.9856 | 1 | 0.9466 | 1 | 1.0289 | 1 | 0.9888 | 1 | 1.0305 | 1 | 1.0155 | 1 | 1.0086 | 1 | 1.0986 | 1 | Lifestyle | Uses hands-free or speakerphone frequently |
| p900_2 | 1.0611 | 1 | 1.0864 | 1 | 0.9904 | 1 | 1.1097 | 1 | 1.2095 | 1 | 1.184 | 1 | 1.0993 | 1 | 1.0922 | 1 | 0.6798 | 1 | Lifestyle | Improper sleep duration <7/>9 hours |
| p6155_2 | 1.1377 | 1 | 1.506 | 0.328658979 | 1.3123 | 1 | 1.052 | 1 | 0.8741 | 1 | 1.3776 | 1 | 1.1441 | 1 | 1.2154 | 1 | 0.8035 | 1 | Chemical | Vitamin B supplements |
| p24508 | 0.9957 | 1 | 1.0305 | 1 | 0.9933 | 1 | 1.0162 | 1 | 1.0383 | 1 | 1.0077 | 1 | 1.0211 | 1 | 0.9968 | 1 | 1.1307 | 1 | Ecosystems | Distance to coast |
| p6154_2 | 0.8336 | 1 | 0.9501 | 1 | 0.6534 | 0.003588753 | 0.973 | 1 | 0.9597 | 1 | 1.1104 | 1 | 0.9015 | 1 | 1.0348 | 1 | 0.9683 | 1 | Chemical | Usually takes Ibuprofen |
| p1170 | 1.0984 | 1 | 1.014 | 1 | 1.2992 | 1.89007E-07 | 1.0633 | 1 | 1.0648 | 1 | 1.0109 | 1 | 1.0701 | 1 | 1.0555 | 1 | 0.9441 | 1 | Lifestyle | Gets up more easily in the morning |
| p1508_1 | 1.0214 | 1 | 0.9519 | 1 | 0.939 | 1 | 0.8979 | 1 | 0.9739 | 1 | 1.0227 | 1 | 0.9654 | 1 | 0.9398 | 1 | 0.999 | 1 | Lifestyle | Instant coffee versus decaffeinated |
| p1428_2 | 0.847 | 1 | 1.1807 | 1 | 1.9289 | 1 | 0.8724 | 1 | 0 | 1 | 2.2803 | 1 | 1.166 | 1 | 1.1295 | 1 | 1.0988 | 1 | Lifestyle | Flora Pro-Active spread versus rare/non-spread |
| p6154_3 | 1.2101 | 0.285369926 | 1.0618 | 1 | 0.9867 | 1 | 1.0998 | 1 | 1.1637 | 1 | 1.1873 | 1 | 1.1136 | 1 | 1.1196 | 1 | 0.9896 | 1 | Chemical | Usually takes Paracetamol |
| p24502 | 0.9687 | 1 | 0.9662 | 1 | 0.9351 | 1 | 0.9702 | 1 | 1.0207 | 1 | 0.9266 | 1 | 0.9567 | 1 | 0.9481 | 1 | 1.0643 | 1 | Ecosystems | Water percentage (1000 m buffer) |
| p1990 | 1.0325 | 1 | 1.1808 | 1 | 0.9144 | 1 | 1.1629 | 1 | 0.9442 | 1 | 1.1501 | 1 | 1.0383 | 1 | 1.0738 | 1 | 1.2394 | 1 | Social | Tense/'highly strung' |
| p2010 | 1.1254 | 1 | 1.0104 | 1 | 0.9284 | 1 | 1.1142 | 1 | 1.155 | 1 | 1.1022 | 1 | 1.023 | 1 | 1.0399 | 1 | 1.3563 | 1 | Social | Suffer from 'nerves' |
| p864 | 0.9894 | 1 | 1.0465 | 1 | 1.0391 | 1 | 1.029 | 1 | 0.8709 | 1 | 1.0483 | 1 | 1.0303 | 1 | 1.0508 | 1 | 0.8751 | 1 | Lifestyle | Days/week walked 10+ minutes |
| p1538 | 0.9973 | 1 | 1.1083 | 1 | 1.0724 | 1 | 0.9909 | 1 | 1.7551 | 0.1682929 | 0.9123 | 1 | 0.9973 | 1 | 0.994 | 1 | 1.1934 | 1 | Lifestyle | Major dietary changes |
| p1940 | 0.9013 | 1 | 0.8401 | 1 | 0.8165 | 1 | 0.9211 | 1 | 0.7472 | 1 | 0.99 | 1 | 0.9315 | 1 | 0.9427 | 1 | 1.1182 | 1 | Social | Irritability |
| p6139_2 | 0.939 | 1 | 0.9668 | 1 | 1.1546 | 1 | 0.902 | 1 | 1.1172 | 1 | 0.9682 | 1 | 0.9467 | 1 | 1.0025 | 1 | 1.0679 | 1 | Chemical | Uses gas fire in winter |
| p1418_1 | 1.2305 | 1 | 0.732 | 1 | 0.9588 | 1 | 1.0919 | 1 | 0.7097 | 1 | 1.1197 | 1 | 1.1211 | 1 | 1.0119 | 1 | 3.7889 | 1 | Lifestyle | Full-cream milk versus rare/non-milk |
| p904 | 1.0057 | 1 | 1.0629 | 1 | 1.0253 | 1 | 1.0092 | 1 | 1.0278 | 1 | 1.048 | 1 | 0.9908 | 1 | 1.077 | 0.067986583 | 1.1724 | 1 | Lifestyle | Days/week of vigorous physical activity |
| p2237 | 0.8966 | 1 | 0.9879 | 1 | 0.8296 | 1 | 0.9683 | 1 | 0.8607 | 1 | 0.8621 | 1 | 0.9394 | 1 | 0.9465 | 1 | 1.2274 | 1 | Lifestyle | Plays computer games frequently |
| p6162_2 | 0.9868 | 1 | 0.9528 | 1 | 1.1724 | 1 | 0.934 | 1 | 0.6096 | 0.926377293 | 0.9867 | 1 | 0.9556 | 1 | 0.9861 | 1 | 0.9202 | 1 | Lifestyle | Walks as transport |
| p22032 | 0.9812 | 1 | 1.0852 | 1 | 1.1284 | 0.97718879 | 1.0034 | 1 | 0.8764 | 1 | 1.0213 | 1 | 1.0336 | 1 | 1.0787 | 1 | 0.9449 | 1 | Lifestyle | IPAQ activity level |
| p22040 | 0.9944 | 1 | 1.0417 | 1 | 1.0754 | 1 | 0.9922 | 1 | 0.9454 | 1 | 1.0128 | 1 | 1.0052 | 1 | 1.0376 | 1 | 0.9134 | 1 | Lifestyle | MET minutes/week of all activity |
| p1220 | 1.0297 | 1 | 1.038 | 1 | 1.2017 | 0.350389321 | 1.0415 | 1 | 1.3472 | 1 | 1.1645 | 1 | 0.9644 | 1 | 1.0529 | 1 | 0.9141 | 1 | Lifestyle | Daytime sleep frequency |
| p1697_1 | 0.9653 | 1 | 1.1102 | 1 | 1.0108 | 1 | 1.0535 | 1 | 1.0599 | 1 | 1.0563 | 1 | 1.0601 | 1 | 1.0889 | 1 | 1.3116 | 1 | Social | Relatively shorter at 10 years old |
| p6155_1 | 0.9388 | 1 | 1.3026 | 1 | 1.1816 | 1 | 0.8865 | 1 | 1.2682 | 1 | 1.3164 | 1 | 0.79 | 1 | 0.9963 | 1 | 0.8765 | 1 | Chemical | Vitamin A supplements |
| p24011 | 0.9985 | 1 | 1.0055 | 1 | 1.0043 | 1 | 0.9935 | 1 | 1.0634 | 1 | 0.9334 | 1 | 1.0007 | 1 | 1.0085 | 1 | 0.938 | 1 | Physical | Traffic intensity on nearest major road |
| p6164_1 | 0.983 | 1 | 0.9596 | 1 | 1.1674 | 1 | 0.8993 | 1 | 0.8414 | 1 | 0.9907 | 1 | 0.947 | 1 | 0.9591 | 1 | 1.4507 | 1 | Lifestyle | Walks for pleasure |
| p1180 | 0.9537 | 1 | 1.0178 | 1 | 0.9123 | 0.949753883 | 0.9588 | 1 | 0.9144 | 1 | 0.9748 | 1 | 0.968 | 1 | 0.9741 | 1 | 0.9837 | 1 | Lifestyle | More evening-type chronotype |
| p22038 | 0.9572 | 1 | 1.0133 | 1 | 1.1009 | 0.801217827 | 0.9761 | 1 | 0.9687 | 1 | 1.0049 | 1 | 0.9875 | 1 | 0.9902 | 1 | 1.1299 | 1 | Lifestyle | MET minutes/week of moderate activity |
| p1508_3 | 0.9971 | 1 | 1.4121 | 1 | 0.9078 | 1 | 1.0835 | 1 | 1.7387 | 1 | 1.4634 | 1 | 0.9669 | 1 | 1.2584 | 1 | 0.5865 | 1 | Lifestyle | Other coffee types versus decaffeinated |
| p20160 | 1.0928 | 1 | 0.978 | 1 | 1.0762 | 1 | 1.0006 | 1 | 1.2096 | 1 | 0.9741 | 1 | 1.0386 | 1 | 1.0791 | 1 | 1.0328 | 1 | Lifestyle | Ever smoked |
| p1428_3 | 0.8643 | 1 | 1.1506 | 1 | 0.9067 | 1 | 0.9459 | 1 | 0.7842 | 1 | 0.9942 | 1 | 0.8418 | 1 | 0.9449 | 1 | 1.1306 | 1 | Lifestyle | Other spreads/margarine versus rare/non-spread |
| p24008 | 0.9822 | 1 | 0.9847 | 1 | 1.0016 | 1 | 0.9908 | 1 | 1.0926 | 1 | 0.9546 | 1 | 0.9945 | 1 | 0.9968 | 1 | 0.9265 | 1 | Chemical | PM2.5-10 pollution |
| p1140_3 | 1.0475 | 1 | 0.7955 | 1 | 0.9029 | 1 | 1.0881 | 1 | 1.5027 | 1 | 1.2318 | 1 | 1.0354 | 1 | 1.0317 | 1 | 0 | 1 | Lifestyle | Didn't use a mobile phone versus no difference |
| p6145_5 | 1.1113 | 1 | 1.1147 | 1 | 0.7634 | 1 | 0.8784 | 1 | 2.2474 | 1 | 1.3637 | 1 | 1.0092 | 1 | 1.0944 | 1 | 1.0355 | 1 | Social | Marital separation/divorce |
| p1687_2 | 0.9758 | 1 | 1.0222 | 1 | 0.9629 | 1 | 0.9066 | 1 | 1.3803 | 1 | 1.114 | 1 | 0.9947 | 1 | 1.0029 | 1 | 0.6344 | 1 | Social | Relatively plumper at 10 years old |
| p894 | 1.0647 | 1 | 1.0508 | 1 | 1.203 | 1.72281E-06 | 1.0613 | 1 | 1.0386 | 1 | 1.0277 | 1 | 1.0309 | 1 | 1.0135 | 1 | 1.0013 | 1 | Lifestyle | Duration of moderate activity |
| p6155_6 | 1.525 | 0.215361639 | 1.3835 | 1 | 1.5861 | 1 | 1.1153 | 1 | 0 | 1 | 1.3959 | 1 | 1.2986 | 1 | 1.4253 | 0.237175485 | 0.7127 | 1 | Chemical | Folic acid or Folate (Vit B9) supplements |
| p6139_1 | 1.0894 | 1 | 0.9203 | 1 | 0.9373 | 1 | 0.9664 | 1 | 1.1737 | 1 | 0.8875 | 1 | 1.0178 | 1 | 1.0345 | 1 | 0.7641 | 1 | Chemical | Uses gas hob or cooker |
| p1190 | 1.0086 | 1 | 0.9333 | 1 | 1.1438 | 1 | 0.9607 | 1 | 1.3137 | 1 | 1.0679 | 1 | 0.8822 | 0.593603557 | 0.9629 | 1 | 1.0068 | 1 | Lifestyle | Naps frequently |
| p6145_1 | 0.9928 | 1 | 1.0326 | 1 | 1.1175 | 1 | 1.0049 | 1 | 0.586 | 1 | 1.0499 | 1 | 0.9538 | 1 | 0.9558 | 1 | 0.7387 | 1 | Social | Own serious illness or injury |
| p1031 | 0.9982 | 1 | 1.0107 | 1 | 1.0977 | 0.234863872 | 1.0084 | 1 | 0.9699 | 1 | 1.0086 | 1 | 1.0156 | 1 | 1.0204 | 1 | 1.0142 | 1 | Social | Friend/family visits frequently |
| p900_8 | 0.9908 | 1 | 0.9506 | 1 | 0.9345 | 1 | 0.9884 | 1 | 0.9455 | 1 | 0.9617 | 1 | 0.9321 | 1 | 0.9515 | 1 | 0.9737 | 1 | Lifestyle | Red meat intake reduced |
| p924 | 1.009 | 1 | 0.9798 | 1 | 0.819 | 0.050701862 | 0.9658 | 1 | 0.7232 | 1 | 0.925 | 1 | 1.0801 | 1 | 1.0147 | 1 | 1.3413 | 1 | Lifestyle | Usually walks quickly |
| p1120 | 0.9805 | 1 | 0.9263 | 1 | 0.8064 | 1.4531E-11 | 1.0147 | 1 | 0.9874 | 1 | 0.9872 | 1 | 1.005 | 1 | 0.9967 | 1 | 0.9869 | 1 | Lifestyle | Weekly mobile phone usage |
| p1478 | 0.9886 | 1 | 1.0122 | 1 | 0.9993 | 1 | 0.9324 | 1 | 1.1499 | 1 | 1.0301 | 1 | 0.9918 | 1 | 0.9982 | 1 | 1.1887 | 1 | Lifestyle | Adds salt to food frequently |
| p6145_3 | 0.971 | 1 | 0.9146 | 1 | 0.9192 | 1 | 0.9419 | 1 | 0.8092 | 1 | 1.021 | 1 | 0.9919 | 1 | 0.9866 | 1 | 0.7952 | 1 | Social | Death of a close relative |
| p981 | 0.9876 | 1 | 1.0105 | 1 | 0.9931 | 1 | 1.0154 | 1 | 0.9808 | 1 | 1.0007 | 1 | 1.0101 | 1 | 1.0024 | 1 | 0.9367 | 1 | Lifestyle | Duration of walking for pleasure |
| p24014 | 0.9235 | 1 | 0.8549 | 1 | 0.8461 | 1 | 0.8706 | 1 | 0.6998 | 1 | 0.9213 | 1 | 1.0485 | 1 | 0.9506 | 1 | 0.7547 | 1 | Physical | Close to major road |
| p1140_1 | 0.8887 | 1 | 0.9768 | 1 | 0.8863 | 1 | 1.0595 | 1 | 0.8824 | 1 | 0.8479 | 1 | 1.0855 | 1 | 1.0195 | 1 | 0.9818 | 1 | Lifestyle | Less frequent mobile phone use versus no difference |
| p1950 | 0.9809 | 1 | 0.9869 | 1 | 0.9073 | 1 | 1.0633 | 1 | 0.9232 | 1 | 0.9588 | 1 | 1.0125 | 1 | 0.971 | 1 | 1.1634 | 1 | Social | Sensitivity/hurt feelings |
| p22034 | 0.9724 | 1 | 1.0411 | 1 | 1.1286 | 0.298647806 | 1.0242 | 1 | 0.9275 | 1 | 1.0296 | 1 | 0.9896 | 1 | 1.0185 | 1 | 0.9038 | 1 | Lifestyle | Summed minutes activity |
| p1070 | 1.0477 | 1 | 1.0065 | 1 | 1.1839 | 0.023818334 | 0.9841 | 1 | 1.0696 | 1 | 0.9932 | 1 | 1.0334 | 1 | 1.0318 | 1 | 0.9295 | 1 | Lifestyle | Time spent watching television |
| p884 | 0.9807 | 1 | 1.0297 | 1 | 1.0669 | 1 | 1.0245 | 1 | 0.9053 | 1 | 1.0139 | 1 | 1.0064 | 1 | 1.0419 | 1 | 1.0322 | 1 | Lifestyle | Days/week moderate physical activity 10+ minutes |
| p22035 | 0.9778 | 1 | 1.0562 | 1 | 1.1345 | 1 | 1.0379 | 1 | 0.9056 | 1 | 1.1044 | 1 | 0.9934 | 1 | 1.0545 | 1 | 0.9336 | 1 | Lifestyle | At or above moderate/vigorous recommendation |
| p6179_2 | 0.9697 | 1 | 1.0382 | 1 | 1.5269 | 2.55463E-06 | 0.9748 | 1 | 0.7394 | 1 | 1.0262 | 1 | 1.0243 | 1 | 1.0061 | 1 | 0.732 | 1 | Chemical | Glucosamine supplements |
| p6160_3 | 1.0329 | 1 | 0.8924 | 1 | 1.3705 | 0.033838956 | 0.9474 | 1 | 0.8642 | 1 | 0.9064 | 1 | 0.9184 | 1 | 0.9778 | 1 | 0.8383 | 1 | Lifestyle | Attends a religious group frequently |
| p1150_1 | 0.9573 | 1 | 1.1826 | 1 | 1.1889 | 1 | 0.9317 | 1 | 0.4152 | 1 | 1.0202 | 1 | 0.9877 | 1 | 1.0122 | 1 | 0.7694 | 1 | Lifestyle | Prefers left side of head for mobile phone use |
| p1970 | 0.9771 | 1 | 1.07 | 1 | 0.9749 | 1 | 1.0135 | 1 | 1.3647 | 1 | 1.0833 | 1 | 0.9706 | 1 | 1.0086 | 1 | 1.2253 | 1 | Social | Nervous feelings |
| p6179_6 | 0.9148 | 1 | 1.0939 | 1 | 1.0227 | 1 | 1.0497 | 1 | 1.0382 | 1 | 1.1615 | 1 | 0.9658 | 1 | 0.9184 | 1 | 1.3781 | 1 | Chemical | Selenium supplements |
| p1498 | 0.9485 | 1 | 1.0168 | 1 | 1.0867 | 1 | 0.9683 | 1 | 1.0918 | 1 | 0.9708 | 1 | 0.9936 | 1 | 0.9944 | 1 | 0.8579 | 1 | Lifestyle | Coffee intake |
| p1707_2 | 0.6768 | 1 | 1.1245 | 1 | 0.7518 | 1 | 1.049 | 1 | 0.9594 | 1 | 0.6688 | 1 | 0.9427 | 1 | 0.8269 | 1 | 1.4391 | 1 | Lifestyle | Left-handed versus ambidextrous |
| p24501 | 0.987 | 1 | 1.0023 | 1 | 1.0187 | 1 | 0.9776 | 1 | 1.0066 | 1 | 0.9589 | 1 | 1.0094 | 1 | 1.0166 | 1 | 0.938 | 1 | Ecosystems | Domestic garden percentage (1000 m buffer) |
| p1249 | 1.009 | 1 | 0.9979 | 1 | 1.0668 | 1 | 1.0062 | 1 | 1.0637 | 1 | 0.99 | 1 | 1.0092 | 1 | 1.0139 | 1 | 1.0352 | 1 | Lifestyle | Past tobacco smoking frequency |
| p1707_1 | 0.7371 | 1 | 1.0987 | 1 | 0.7656 | 1 | 0.9985 | 1 | 1.0021 | 1 | 0.7385 | 1 | 1.0168 | 1 | 0.8885 | 1 | 1.0333 | 1 | Lifestyle | Right-handed versus ambidextrous |
| p1677 | 1.1317 | 1 | 0.9015 | 1 | 1.3808 | 0.002162338 | 0.9903 | 1 | 0.9486 | 1 | 1.0012 | 1 | 1.0125 | 1 | 1.0224 | 1 | 1.0138 | 1 | Chemical | Breastfed as a baby |
| p900_5 | 1.036 | 1 | 0.9842 | 1 | 1.1646 | 1 | 1.0516 | 1 | 1.0561 | 1 | 0.9604 | 1 | 0.998 | 1 | 0.9759 | 1 | 1.5078 | 1 | Lifestyle | Fish eaten above recommendation |
| p6155_5 | 0.8307 | 1 | 1.2815 | 1 | 1.1817 | 1 | 0.981 | 1 | 0.8848 | 1 | 1.1029 | 1 | 1.0361 | 1 | 1.0884 | 1 | 0.7417 | 1 | Chemical | Vitamin E supplements |
| p24503 | 0.9179 | 0.349114931 | 1.0122 | 1 | 0.978 | 1 | 0.9906 | 1 | 0.9192 | 1 | 1.0044 | 1 | 0.958 | 1 | 0.9988 | 1 | 1.0304 | 1 | Ecosystems | Greenspace percentage (300 m buffer) |
| p1960 | 1.0695 | 1 | 1.0089 | 1 | 0.8001 | 0.170832971 | 1.0718 | 1 | 1.0876 | 1 | 1.0863 | 1 | 1.0883 | 1 | 1.084 | 1 | 1.3916 | 1 | Social | Fed-up feelings |
| p1687_1 | 1.0208 | 1 | 1.0217 | 1 | 0.9811 | 1 | 1.0487 | 1 | 1.1595 | 1 | 0.9768 | 1 | 1.0458 | 1 | 1.0798 | 1 | 0.8593 | 1 | Social | Relatively thinner at 10 years old |
| p6154_5 | 1.033 | 1 | 1.0076 | 1 | 1.1351 | 1 | 1.1258 | 1 | 1.2018 | 1 | 0.9601 | 1 | 0.9726 | 1 | 0.9807 | 1 | 1.3931 | 1 | Chemical | Usually takes Omeprazole |
| p24009 | 0.9864 | 1 | 0.9513 | 1 | 0.8895 | 0.641472908 | 0.9633 | 1 | 0.9014 | 1 | 0.9467 | 1 | 1.0035 | 1 | 0.9685 | 1 | 0.9879 | 1 | Physical | Traffic intensity on nearest road |
| p6160_2 | 1.1455 | 1 | 0.9772 | 1 | 0.9821 | 1 | 0.994 | 1 | 1.0715 | 1 | 0.9434 | 1 | 1.0947 | 1 | 1.0483 | 1 | 0.7841 | 1 | Lifestyle | Attends a pub or social club frequently |
| p6162_3 | 0.9659 | 1 | 0.9525 | 1 | 1.3818 | 0.000284823 | 0.9705 | 1 | 1.3279 | 1 | 1.0021 | 1 | 0.9849 | 1 | 1.0178 | 1 | 1.0907 | 1 | Lifestyle | Uses public transport |
| p22033 | 0.9648 | 1 | 1.0357 | 1 | 1.0929 | 1 | 1.0141 | 1 | 0.9089 | 1 | 1.0167 | 1 | 0.9845 | 1 | 1.0201 | 1 | 0.9282 | 1 | Lifestyle | Summed days activity |
| p24022 | 1 | 1 | 0.983 | 1 | 0.9872 | 1 | 0.9819 | 1 | 1.0307 | 1 | 0.9891 | 1 | 0.9938 | 1 | 0.971 | 1 | 1.0153 | 1 | Physical | Average noise level (nighttime) |
| p1930 | 0.9402 | 1 | 1.0556 | 1 | 0.7687 | 0.014883312 | 0.9987 | 1 | 1.0574 | 1 | 1.0154 | 1 | 1.0051 | 1 | 0.9719 | 1 | 1.2311 | 1 | Social | Miserableness |
| p24021 | 0.9998 | 1 | 0.983 | 1 | 0.987 | 1 | 0.9817 | 1 | 1.0298 | 1 | 0.9888 | 1 | 0.994 | 1 | 0.9711 | 1 | 1.0152 | 1 | Physical | Average noise level (evening) |
| p24020 | 0.9996 | 1 | 0.9831 | 1 | 0.9868 | 1 | 0.9816 | 1 | 1.0292 | 1 | 0.9886 | 1 | 0.9942 | 1 | 0.9711 | 1 | 1.0149 | 1 | Physical | Average noise level (daytime) |
| p6179_3 | 1.0303 | 1 | 0.9145 | 1 | 1.4318 | 0.319486656 | 1.002 | 1 | 0.8346 | 1 | 0.9477 | 1 | 1.1054 | 1 | 1.0306 | 1 | 0.4813 | 1 | Chemical | Calcium supplements |
| p1418_2 | 0.9265 | 1 | 0.8393 | 1 | 0.8871 | 1 | 1.1114 | 1 | 0.8028 | 1 | 0.9974 | 1 | 1.0275 | 1 | 0.9576 | 1 | 2.7315 | 1 | Lifestyle | Semi-skimmed milk versus rare/non-milk |
| p22037 | 1.0031 | 1 | 1.0019 | 1 | 1.06 | 1 | 1.0578 | 1 | 0.9089 | 1 | 1.0185 | 1 | 0.9965 | 1 | 1.0265 | 1 | 0.9981 | 1 | Lifestyle | MET minutes/week of walking |
| p100_1 | 1.0372 | 1 | 1.0518 | 1 | 1.2305 | 0.24587971 | 1.0776 | 1 | 0.9193 | 1 | 0.9635 | 1 | 1.0637 | 1 | 1.0555 | 1 | 1.2039 | 1 | Lifestyle | Healthy diet |
| p24010 | 1.0255 | 1 | 1.0094 | 1 | 1.0307 | 1 | 0.9902 | 1 | 1.1932 | 1 | 0.9839 | 1 | 0.9921 | 1 | 0.9801 | 1 | 1.0679 | 1 | Physical | Inverse distance to road |
| p20127 | 0.9799 | 1 | 0.9889 | 1 | 0.8958 | 0.062814823 | 1.0088 | 1 | 0.9831 | 1 | 1.0002 | 1 | 0.9928 | 1 | 0.9936 | 1 | 1.1427 | 1 | Social | Neuroticism score |
| p1418_3 | 0.919 | 1 | 0.7701 | 1 | 0.9792 | 1 | 1.0935 | 1 | 0.9398 | 1 | 1.0571 | 1 | 0.9998 | 1 | 0.9427 | 1 | 3.4246 | 1 | Lifestyle | Skimmed milk versus rare/non-milk |
| p24507 | 0.9265 | 0.830298526 | 1.0308 | 1 | 0.972 | 1 | 0.962 | 1 | 0.9531 | 1 | 0.9765 | 1 | 0.9473 | 1 | 0.9909 | 1 | 1.0271 | 1 | Ecosystems | Natural environment percentage (300 m buffer) |
| p24504 | 1.0272 | 1 | 1.001 | 1 | 1.0485 | 1 | 1.0133 | 1 | 1.0659 | 1 | 0.9519 | 1 | 1.0116 | 1 | 1.0154 | 1 | 0.92 | 1 | Ecosystems | Domestic garden percentage (300 m buffer) |

**Notes:** P represents the Bonferroni-adjusted P value. AD = Alzheimer’s Disease.

**Supplementary Table 12. Cross-validation performance of XGBoost models across subgroups for ADRD and AD**

|  | **All incident ADRD** | | | | | | **All incident AD** | | | | | |
| --- | --- | --- | --- | --- | --- | --- | --- | --- | --- | --- | --- | --- |
|  | **Average AUC (SD)** | **Fold1_**  **AUC** | **Fold2_**  **AUC** | **Fold3_**  **AUC** | **Fold4_**  **AUC** | **Fold5_**  **AUC** | **Average AUC (SD)** | **Fold1_**  **AUC** | **Fold2_**  **AUC** | **Fold3_**  **AUC** | **Fold4_**  **AUC** | **Fold5_ AUC** |
| **All** | 0.636±0.008 | 0.643 | 0.625 | 0.63 | 0.639 | 0.642 | 0.609±0.022 | 0.644 | 0.604 | 0.615 | 0.589 | 0.595 |
| **Female** | 0.645±0.018 | 0.663 | 0.641 | 0.654 | 0.649 | 0.616 | 0.589±0.02 | 0.561 | 0.609 | 0.607 | 0.579 | 0.586 |
| **Male** | 0.626±0.003 | 0.624 | 0.624 | 0.625 | 0.631 | 0.627 | 0.594±0.019 | 0.612 | 0.605 | 0.605 | 0.575 | 0.571 |
| **Age<65** | 0.733±0.018 | 0.751 | 0.723 | 0.716 | 0.754 | 0.721 | 0.74±0.024 | 0.751 | 0.726 | 0.713 | 0.776 | 0.734 |
| **Age≥65** | 0.616±0.01 | 0.617 | 0.601 | 0.628 | 0.619 | 0.616 | 0.595±0.031 | 0.55 | 0.616 | 0.589 | 0.589 | 0.631 |
| **Low genetic risk** | 0.64±0.042 | 0.653 | 0.612 | 0.6 | 0.705 | 0.631 | 0.624±0.051 | 0.627 | 0.606 | 0.61 | 0.567 | 0.707 |
| **Intermediate genetic risk** | 0.638±0.009 | 0.645 | 0.626 | 0.631 | 0.646 | 0.644 | 0.597±0.013 | 0.615 | 0.585 | 0.591 | 0.588 | 0.606 |
| **High genetic risk** | 0.612±0.012 | 0.598 | 0.627 | 0.603 | 0.621 | 0.608 | 0.589±0.036 | 0.627 | 0.559 | 0.544 | 0.617 | 0.597 |
| **White British** | 0.636±0.011 | 0.644 | 0.627 | 0.622 | 0.643 | 0.644 | 0.609±0.027 | 0.6 | 0.652 | 0.594 | 0.618 | 0.581 |
| **Other Ethnicities** | 0.657±0.054 | 0.63 | 0.635 | 0.673 | 0.604 | 0.743 | — | — | — | — | — | — |

**Notes:** Values represent the mean AUC (± standard deviation) derived from five-fold cross-validation on the training dataset, followed by individual AUC values for each fold. ADRD = Alzheimer's disease and related dementias; AD = Alzheimer's disease; AUC = area under the receiver operating characteristic curve. "—" indicates insufficient sample size for model training in the Other Ethnicities subgroup for AD.

**Supplementary Table 13. Model performance metrics for ADRD and AD prediction**

|  | **Models** | **Accuracy** | **Balanced Accuracy** | **Sensitivity** | **Specificity** | **Precision** | **AUC** | **AUPRC** |
| --- | --- | --- | --- | --- | --- | --- | --- | --- |
| **All incident ADRD** | Exposures + Demographics + *APOE* status | 0.700±0.028 | 0.664±0.030 | 0.627±0.030 | 0.701±0.029 | 0.037±0.002 | 0.718±0.005 | 0.044±0.002 |
|  | Exposures | 0.651±0.039 | 0.637±0.041 | 0.623±0.041 | 0.651±0.040 | 0.032±0.002 | 0.678±0.005 | 0.035±0.001 |
|  | Demographics + *APOE* status | 0.784±0.012 | 0.664±0.015 | 0.538±0.016 | 0.789±0.013 | 0.045±0.002 | 0.680±0.005 | 0.037±0.001 |
| **All incident AD** | Exposures + Demographics + *APOE* status | 0.742±0.010 | 0.675±0.013 | 0.607±0.016 | 0.743±0.010 | 0.019±0.001 | 0.724±0.007 | 0.022±0.001 |
|  | Exposures | 0.607±0.025 | 0.645±0.026 | 0.683±0.027 | 0.606±0.025 | 0.014±0.001 | 0.680±0.007 | 0.016±0.001 |
|  | Demographics + *APOE* status | 0.795±0.004 | 0.683±0.009 | 0.569±0.014 | 0.797±0.004 | 0.023±0.001 | 0.693±0.008 | 0.020±0.001 |

**Notes:** Values are presented as mean ± standard deviation based on 1,000 bootstrap resamples. Cut-offs were determined based on the achievement of the largest Youden index (YI = sensitivity + specificity - 1). ADRD = Alzheimer’s Disease and Related Dementias, AD = Alzheimer’s Disease, AUC = Area under the ROC Curve, AUPRC=Area Under the Precision-Recall Curve.
